# Unveiling the Defenses: A Current and Comprehensive Review of Coleoptera Carabidae Strategies

**DOI:** 10.3390/biology14060709

**Published:** 2025-06-17

**Authors:** Teresa Bonacci

**Affiliations:** 1 Department of Biology, Ecology and Earth Sciences, University of Calabria, 87036 Rende, CS, Italy; teresa.bonacci@unical.it; 2 Sistema Museale Universitario, SiMU, sezione di Zoologia, University of Calabria, 87036 Rende, CS, Italy

**Keywords:** antipredator strategies, carabids, aposematism, chemicals, predators

## Abstract

The Carabidae family, or ground beetles, is a wide and ecologically significant group within the Coleoptera order, known for its role as natural predators of agricultural pests and as bioindicators of ecosystem health. These beetles employ a variety of behavioral, morphological, and chemical defense strategies to protect themselves from predators. These mechanisms include gregariousness, stridulation, regurgitation, and chemical defenses, such as the secretion of irritating compounds from specialized glands. The defensive strategies of carabids are classified into passive and active systems, each with varying energetic costs. Chemical substances (e.g., Formic acid, Methacrylic acid, Tiglic acid, Ethacrilic acid, Isovaleric acid, Salicylaldehyde, 1,4-Benzoquinone, Toluquinone, 13-2Kt tridecan-2-one, Undecane, Tridecane, Pentadecane, M-cresol) are particularly important, as they serve to deter predators and combat pathogens like bacteria and fungi. Ground beetles utilize both polar and non-polar compounds in their defense, all contributing to their ecological success. This review explores the array of defensive mechanisms in the Carabidae family, highlighting experimental studies, field observations, and reviews published over the last five decades. The aim is to provide a comprehensive understanding of how these strategies enhance the survival and fitness of carabid beetles in their natural environments.

## Simple Summary

This review synthesizes the current knowledge on the defensive adaptations of Carabidae, a taxonomically and ecologically diverse beetle family. Emphasis is placed on the structural, behavioral, and chemical strategies employed to mitigate predation and microbial threats. Particular attention is given to chemical defenses mediated by exocrine secretions, which constitute a central component of their antipredator mechanisms. The analysis integrates findings from experimental studies, field observations, datasets, and comparative reviews spanning the past five decades, with the aim of elucidating the evolutionary significance and ecological function of their mechanisms in promoting their survival and their fitness in natural habitats. Among the various strategies identified are gregarious behavior, stridulation, regurgitation, and the use of chemicals from specialized glands. Carabid beetles employ both passive and active defense systems: passive defenses rely on endogenous toxins stored in body tissues, whereas active mechanisms involve the deployment of secretions or aggressive responses when threatened. Chemical defenses are particularly significant, as they not only deter predators but also provide protection against pathogens such as bacteria and fungi.

## 1. Introduction

The family Carabidae is characterized by extensive taxonomic and ecological diversity, comprising a wide array of subfamilies, tribes, and genera that differ in their ecological roles and morphological traits. Among them, groups such as Pterostichinae, Carabinae, and Harpalinae have been more extensively studied, largely due to their abundance, ease of collection, and ecological significance. Many species within these groups are recognized as key predators of agricultural and forest pests, which has drawn considerable research attention from both ecological and applied perspectives [1,2]. Additionally, their sensitivity to environmental changes has made them valuable bioindicators in conservation and habitat quality assessments. In contrast, less attention has been paid to more cryptic or taxonomically complex lineages, often due to identification difficulties or limited knowledge of their biology and distribution [3,4]. Found in various ecosystems across the globe, members of this family exhibit a range of behavioral, morphological, and chemical defensive strategies to protect themselves from predators. These mechanisms are crucial for their survival in competitive environments [5].

Adults and larvae of insects, including carabid beetles, employ a variety of strategies to fend off enemies and pathogens [6]. Insect–predator interactions, like those in other animals, have driven the evolution of behavioral mechanisms such as gregariousness, stridulation, regurgitation of oral secretions, tactile interactions, as well as chemical strategies, morphological adaptations, and color displays, including crypsis, aposematism, and mimicry [7]. All these defensive mechanisms, categorized as passive and active systems, are characterized by different energetic costs [8].

Among the Exapoda, the role of chemical substances in deterring predators [9,10] or combating pathogens (e.g., bacteria and fungi) [11] has been well documented [12,13]. Many insects possess specialized glands where repellent or irritating chemicals are produced and stored [14,15], and ready to be used against predators [16]. Additionally, exocrine glands and other chemicals stored in body fluids are effective against a variety of pathogens [17,18,19]. Within the Coleoptera, a wide array of defensive strategies and chemicals are employed against enemies through both passive and active systems. The passive system relies on toxic substances that are already present in the tissues without requiring active behavioral engagement. In contrast, the active systems involve both the use of chemicals stored in exocrine glands, which are sprayed or released when the insect is disturbed or threatened, and/or the display of aggressive behavior towards enemies. In beetles, the chemicals also serve as fungicides and bactericides, protecting them from a wide range of pathogens [20,21,22,23,24].

In the Coleoptera Adephaga, ground beetles utilize a combination of polar and non-polar compounds in their defense [14,15,23,24], which has contributed significantly to their success in terrestrial ecosystems. The Carabidae family includes species that not only use chemical defenses but also rely on morphological and behavioral mechanisms to deter predators [25,26,27], although few authors [9,13] have reported on the primary vertebrate predators of carabids. Among these predators, insectivores such as owls, rodents, reptiles, and amphibians have been observed hunting ground beetles [28,29,30]. Invertebrates, such as Diptera (Asilidae), Hymenoptera (Formicidae), and Coleoptera (Staphylinidae), have been observed preying on Cicindelinae and other carabid species [31,32]. The objective of this review is to explore the wide array of defensive adaptations employed by Carabidae, focusing on behavioral mechanisms, morphological traits, and chemical defenses, as well as how these strategies enhance fitness and survival in natural habitats. Compared to the last review on this topic [27], this contribution is based on an extensive search of relevant studies published in academic journals. The studies were selected according to the defensive strategies, with a particular emphasis on experimental studies, field observations, and reviews from the past five decades. Both primary research and comprehensive reviews were included, and the studies were grouped based on the primary defensive mechanisms they investigated.

## 2. Behavioral and Morphological Defenses

**Figure 1:**
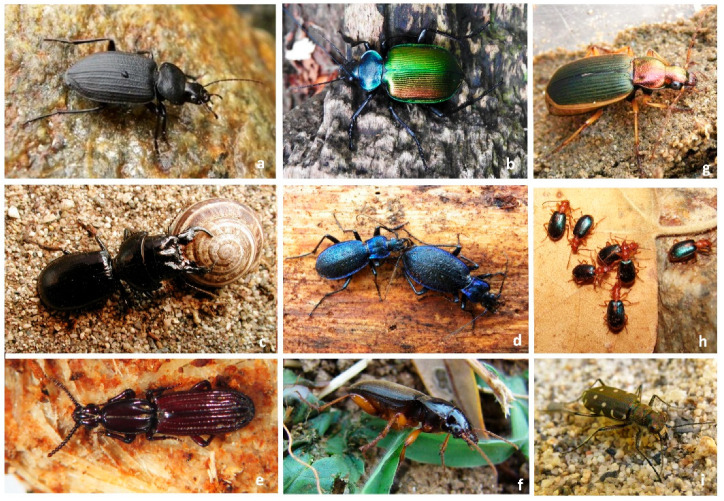
Defensive strategies in ground beetles (Carabidae). The figure illustrates the main types of defense mechanisms observed in carabid beetles, including flight, observed in many macropterous species (*Licinus silphoides*) (**a**); fast running or hiding (*Calosoma sycophanta*) (**b**); mechanical defense (e.g., hardened exoskeleton and large mandibles) (*Scarites buparius* and *Carabus lefebvrei*) (**c**,**d**); behavioral responses such as thanatosis (death-feigning) (*Clinidium canaliculatum*) (**e**); nocturnal activity (*Pseudophonus rufipes*) (**f**) and chemical defense (e.g., spray of noxious compounds in bombardier beetles (*Brachinus sclopeta* and *Chlaenius velutinus*) (**g**,**h**), cryptic coloration for camouflage (*Calomera littoralis* (**i**). These adaptations help carabids avoid predation and increase survival in diverse habitats.

Behavioral strategies vary widely across Carabidae species. One of the most common behavioral defenses is flight, observed in many macropterous species of Carabidae when threatened by predators (Figure 1a). Flight provides an immediate escape from danger, although some species exhibit poor flight capability, relying instead on fast running, rapid burrowing, or hiding (Figure 1b). Other species utilize aggressive posturing to deter predators, such as lunging or displaying intimidating body structures like sharp mandibles [4], or a hard exoskeleton (Figure 1c–e). An interesting behavioral defense found in some species is playing dead (thanatosis) when disturbed, a mechanism that can confuse predators and reduce predation risk [5]. Many Carabidae are also known for nocturnal activity, which helps avoid diurnal predators [5] (Figure 1f).

Insects use chemicals (allomones and pheromones) for social interactions, mating, alarm, defense, camouflage, and food-seeking [33]. In the field of chemical ecology, the avoidance mechanisms of insects involving chemical mimicry consist of the production of substances via biochemical systems or by sequestering or absorbing chemicals from the environment [33,34]. The chemical role of olfaction and gustation in arthropods and how these have favored the evolution of chemical mimicry systems in numerous groups was extensively discussed [35]. Chemical mimicry in carabids is poorly documented. Some species living in or near the nests of social insects imitate the odor of the model to avoid detection and attack by the colony members. Two described cases involve the larvae of *Thermophilum* (=*Anthia*) Weber, 1801 [36] and the adults of *Siagona europaea* Dejean, 1826, both of which use ants as models. In fact, both carabids prey on ant workers and offspring that move near or within the host colony [36,37] without incurring attacks. In the Paussini, which displays myrmecophilous or myrmecophagous habits, morphological, behavioral, chemical, and acoustical strategies have been reported [38,39,40]. Among insects, a common strategy to avoid attacks by visual predators is cryptic coloration, which makes prey difficult to detect [41,42]. This mechanism is known in several preimaginal and adult stages (eggs, larvae, pupae, and adults) of many insect species. Carabids display morphological and color patterns that help them blend in with their surroundings and avoid detection by predators. Some species have cryptic forms that increase their chances of not being spotted by predators, while others have evolved the opposite strategy, becoming conspicuous to predators [43]. This last strategy, known as aposematism, involves color patterns that are common among many insect species [44]. Carabids are less likely than other insects to evolve mimicry of leaves or twigs (cryptic mimicry); however, due to their dark or brown coloration, many of them blend into the ground and are, therefore, overlooked by predators [3]. Some Cicindelinae are nearly indistinguishable from the sandy soils in which they live (Figure 1i), while other species use warning colors to signal their dangerousness to potential predators [45,46]. As is known, conspicuousness in insects reduces the number of attacks from naïve predators, either because of novelty or aversive colors [47,48,49,50]. Aposematism in carabids was first reported by Lindroth [51] in the genus *Lebia* Latreille, 1802. Some Lebistina species from South Africa have evolved a similar color pattern to the poisonous flea beetles *Diamphidia* Obraztsov, 1961, and *Polyclada* Blanchard, 1845. Among the ground beetles, it was also found that *Anchomenus dorsalis* mimics the cuticular profile of *Brachinus sclopeta* as an effective antipredator strategy [52]. More recently, in the genus *Ceroglossus*, which displays conspicuous patterns [52], a Müllerian mimicry strategy has been hypothesized. A black-and-yellow pattern was observed in *Eurycoleus* Chaudoir, 1848 larvae, which prey on the pupae of an endomychid beetle [53]. Mimicry in Cicindelini and Graphipterini was reported in *Elliptica flavovestita* Fairmaire, 1884, *Lophyra wajirensis* Miskell, 1978, and *Neolaphyra leucosticte* (Fairmaire, 1859), species that show similar patterns to the sympatric *Graphipterus* Latreille, 1802 species, which are unpalatable to vertebrate predators [54].

## 3. Protective Group Behavior

In addition to morphological and physical traits, carabids have other behavioral mechanisms that reduce the likelihood of being preyed upon. Gregarious carabids are able to employ multiple strategies simultaneously to defend against enemies. Unlike most carabid beetles, which are typically brown or brown-black, some chemically protected *Brachinus* Weber species are bright orange-red with blue or green elytra; this coloration is likely an aposematic signal [29]. *Anchomenus* dorsalis Pontoppidan, 1736, which is often found aggregating with *Brachinus* species, displays a similar color pattern to bombardier beetles (green-blue and red-brown) and produces defensive chemicals [52] (Figure 1h). The effectiveness of the gregarious behavior of conspicuous and chemically protected species against different predators was evaluated through laboratory tests. The experiments demonstrated a significant predator preference for non-chemically protected and non-visually conspicuous prey [55].

Many advantages can be shared by animals that group together, and of course, grouping is an adaptive strategy for both vertebrates and invertebrates in avoiding predation [56] (Figure 1g). Some authors have suggested that in insects, gregariousness enhances the effect of aposematic signals [6,49,57]. These features, observed in several taxa, appear to increase the signal’s efficiency, influencing both the initial unconditioned aversion of naïve predators and the speed and memorability of avoidance learning [58]. Among carabids, these adaptive relationships are well-documented, and often individuals associate with others of the same species or different species, at least during certain periods of their life cycle [3]. Aggregations have been described for adults of *Anchomenus dorsalis*, *Nebria brevicollis* (Fabricius, 1792), and *Brachinus crepitans* (Linnaeus, 1758) [59]. *Brachinus* (*Brachynidius*) *sclopeta* (Fabricius, 1792) and *Brachinus explodens* Duftschmid, 1812 [60], *Brachinus variventris* L. Schaufuss, 1862, and *Colliuris batesi* (Chaudoir, 1863) [61]. *A. dorsalis* is usually found in small groups within aggregations of protected *Brachinus* and *Chlaenius* Bonelli, 1810 species [52,60,62,63]. Lindroth [62] also observed interspecific interactions between *A. dorsalis* and *Brachinus* species members, which were described in detail as “rubbing behavior” by Zetto Brandmayr et al. [64]. Conspicuous interspecific aggregations, consisting of chemically protected *Chlaenius chrysocephalus* (Rossi, 1790) (60% of total carabid specimens in the aggregation), *Brachinus brevicollis* Motschulsky, 1844 (14.84%), *B. crepitans* (8.63%), *Anchomenus dorsalis* (5.52%), *B. psophia* Audinet-Serville, 1821 (4.66%), and *B. sclopeta* (2.015%) [63] were reported in southern Italy.

*Metrius contractus* Eschscholtz, 1829, a non-aposematic but chemically protected carabid, discharges its defensive secretion as a froth, and this has been observed in group behavior in laboratory settings [65]. The adults of *Calomera plumigera* (Horn, 1892) and *C. chloris* (Hope, 1831) exhibit gregarious diurnal roosting (communal roosting) [66]; the individuals expose their abdomens outward to increase the effectiveness of their chemical defenses (benzaldehyde and benzoyl cyanide) against predators. In general, aggregation occurs in a limited number of carabid species, and its evolutionary significance may primarily be related to protection from water loss and the keeping together of the sexes [3]. In these aggregations, greater success is linked to Müllerian mimicry, where two or more protected species share a similar warning pattern [52]. In this form of mimicry, mimics armed with different defensive strategies are better protected than those sharing a single defensive chemical [67,68]. The ability to repel predators in these intraspecific aggregations is more prolonged. Indeed, bombardier beetles can discharge their defensive spray multiple times, after which they are temporarily unprotected from vertebrate predators [28,29].

## 4. Other Behavioral Strategies

Other particular mechanisms evolved in the larvae of ground beetles are aimed at avoiding intra-specific predation (e.g., cannibalism). Typically, all carabid larvae with predatory habits are very aggressive toward other larvae, including conspecifics [4]. Aggressive and cannibalistic behaviors in the larvae of ground beetles have been reported by many authors [3,69,70,71,72,73,74]. In *Chlaenius velutinus* (Duftschmid, 1812) and *C. spoliatus* (Rossi, 1792) larvae, a typical behavior was identified for the first time, displayed by the larvae through their long cerci during encounters [75,76,77]. In natural environments, these two species live in dense populations, and contacts between conspecifics are very frequent. Unlike other species, larvae of the two *Chlaenius* species avoid cannibalism, possibly as a consequence of a behavioral display involving cerci interactions. This “cerci interaction,” never recorded in other carabid species, inhibits cannibalism through intra-specific recognition. It likely evolved under the pressure of ecological factors such as temporary habitats, ephemeral food availability, and high larval density. Similar behavior was detected in the Paussinae group. *Pachyteles* larvae adopt multiple defensive strategies depending on the context. When exposed, they initially attempt to escape, but if disturbed, they assume a scorpion-like posture, raising the terminal disk of the abdomen over the head and displaying open mandibles to protect vulnerable body parts. They may also secrete dark fluids from the mouth, likely containing digestive enzymes, as a chemical deterrent. Additionally, before molting or pupation, the larva seals its burrow with a soil plug, molded with its mouthparts, to protect the metamorphic process. Once development is complete, it removes the plug using its mandibles [78]. *Chlaenius cordicollis* larvae utilize their metathoracic gland and its associated odor as a defensive response to aggressive stimuli [79].

## 5. Stridulation

Many insects use stridulation to deter predators [80]. Defense sound production is reported in several insect orders. These mechanisms include stridulation, percussion, tymbalation, tremulation and forced air [81]. The sound production in carabids has been less investigated compared to other arthropods. Stridulation or chirping is known in *Cicindela* Linnaeus, 1758 [82,83,84], *Elaphrus* Fabricius, 1775 [85,86,87], *Cychrus* Fabricius, 1794 [3,88,89], *Scaphinotus* Dejean, 1826 [27], *Carabus* Linnaeus, 1758 [86], in ground sand beetles *Omophron* Latreille, 1802 spp. [81], and in *Agonum marginatum* (Linnaeus, 1758) and *Amara familiaris* (Duftschmid, 1812) [90]. Although the most accepted adaptive interpretation of stridulation is that it serves as an additional defense against larger predators, recent studies by [91] have shown that in Paussini, stridulation plays a key role in mate recognition and courtship. Bauer [87] demonstrated that in *Elaphrus cupreus* Duftschmid, 1812, the production of stridulation is an effective mechanism to limit attacks by the bird *Actitis hypoleucos* (Linnaeus, 1758).

## 6. Physical and Behavioral Characteristics

Ground beetles have developed several physical characteristics to enhance their survival (e.g., *Carabus* spp.) (Figure 1d). Among these, their hard, often thick exoskeletons provide protection against physical attacks. This armor serves as a defense against predation by larger animals [4]. Carabids are typically fast runners (e.g., tiger beetles) (Figure 1i), capable of quickly escaping predators by running into vegetation, crevices, or under rocks [3], or by burrowing into the soil (Figure 1c) or under barks. They often remain motionless, relying on their camouflage to avoid detection [5] (Figure 1e). Recent studies report a reduction in the activity of some spermophagous carabid species (e.g., *Harpalus pensylvanicus* DeGeer, *Pterostichus melanarius* Illiger) when exposed to predation cues from mice or from carnivorous carabids [92,93,94,95].

Some species can become aggressive when disturbed, attempting to bite or using their powerful mandibles to fend off attackers [3] (Figure 1c). Although ground beetles are primarily ground-dwelling, many species are capable of flight and may fly to a new location to escape danger (Figure 1a,b). Ground beetles are also at risk of parasitic infections, such as parasitic wasps that lay their eggs on or inside the beetles. Some species engage in cleaning behaviors to rid themselves of parasites, such as grooming or laying eggs in areas less prone to parasitism [3].

## 7. Chemical Defenses

Chemical defense is one of the most widespread and evolutionarily successful defensive adaptations among ground beetles, in addition to morphological and behavioral traits [96]. The ability to synthesize, store, and deploy over 250 chemical compounds provides a highly effective and direct deterrent against predators, surpassing many physical defenses. The anatomical specialization of the pygidial glands and the widespread use of glandular secretions across carabid tribes underscore the central role of chemical defense in their survival strategies [97].

**Figure 2:**
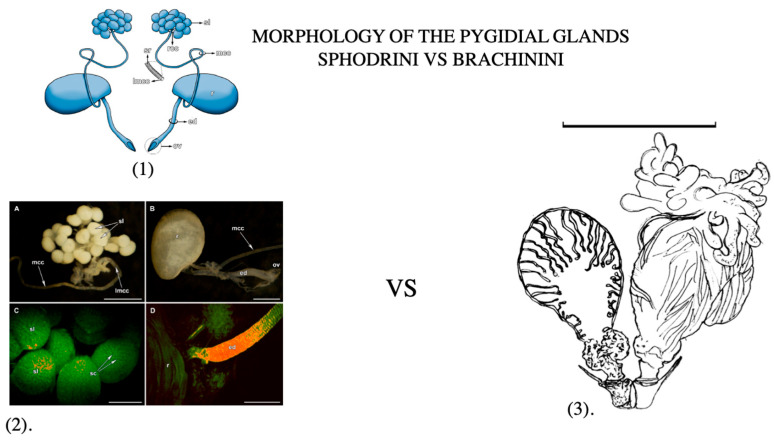
(**1**). General appearance of the pygidial glands of Sphodrini ground beetles. Abbreviations: ed—efferent duct; lmcc—lumen of main collecting canal; mcc –main collecting canal; ov—opening valve; r—reservoir; rcc—radial collecting canal; sl—secretory lobe; sr—spiral ridge [110]. (**2**). Pygidial glands of *Calathus fuscipes*: (**A**) a cluster of the secretory lobes; (**B**) the reservoir and the efferent duct; (**C**) a close-up view of the secretory lobes; (**D**) a close-up view of the junction of the reservoir and the efferent duct (ed—efferent duct; lmcc—lumen of main collecting canal; mcc—main collecting canal; ov—opening valve; r—reservoir; sc—secretory cells; sl—secretory lobes. Scales: (**A**,**B**) 0.5 mm; (**C**) 100 μm; (**D**) 50 μm [110]. (**3**). Pygidial gland and accessory components of the bombardier beetle defense system (after Forsyth). Scale bar = 1.0 mm [111].

**Figure 3:**
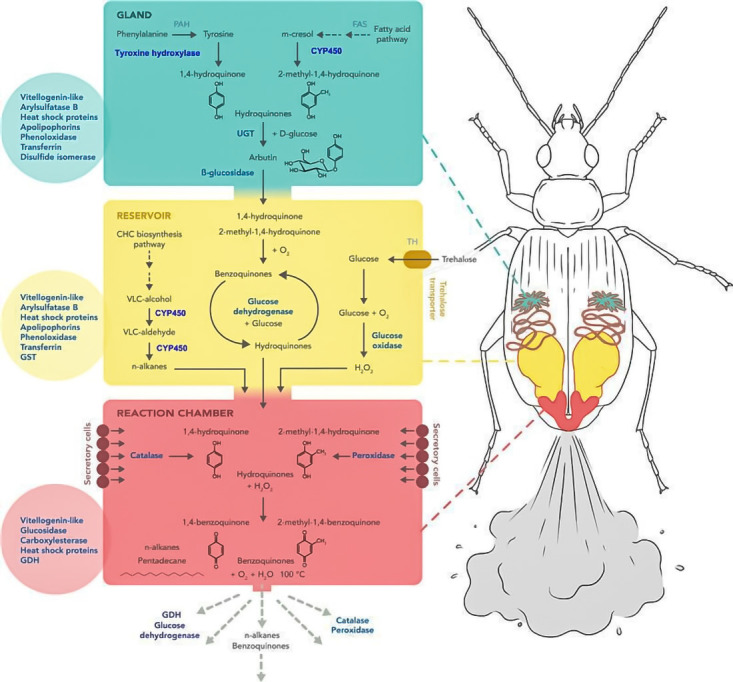
Proposed molecular basis of defensive gland secretions in the bombardier beetle *B. crepitans* based on combined transcriptomic and proteomic analysis. The principal reactions in the MGS with gland (green), RC (yellow) and RXC (red) are shown in black, with candidate genes identified in the transcriptome and the corresponding proteins identified by proteomic analysis shown in blue. The circles show additional abundant proteins identified in the corresponding parts of the defensive gland [109].

Many adults and larvae of carabids produce noxious substances [79], stored in their tissues or specialized glandular apparatus (Figure 2) [20,90,91]. In adults, the pygidial glands are the primary structures responsible for producing and accumulating a wide variety of defensive secretions [24,79,98,99], and all references on the glandular defensive chemicals are in Supplementary S1. When a carabid is disturbed, the pygidial glands are used to discharge chemicals from the tip of the abdomen, deterring potential enemies [100]. In the subfamilies Brachininae and Paussinae, a sclerotized, conical reaction chamber enables precise ejection of allomones [101,102,103]. This chamber includes a one-way valve separating it from the reservoir, with the accessory glands likely producing oxidative enzymes [104,105]. When the valve opens, hydroquinones and hydrogen peroxide mix and react to form p-benzoquinones. The exothermic reaction generates high pressure and heat (up to 100 °C), forcibly ejecting the hot mixture at high velocity and precision [106,107,108]. This highly specialized mechanism, known as crepitation, is unique compared to the oozing or light spraying observed in other carabid species [79]. Recent transcriptomic and proteomic analyses of the defensive glands in the bombardier beetle *Brachinus crepitans* have identified genes and proteins potentially involved in recharging the glands after each defensive explosion (Figure 3) [109]. These findings suggest molecular-level adaptations supporting the efficiency and repeatability of chemical defense in bombardier beetles.

Building on the initial comparative investigations of glandular products by Eisner et al. [97], subsequent studies have focused on the identification of defensive chemicals and their role in chemical ecology [23,24,25,26,98,110], and all the listed references can be found in Supplementary S1. Compounds in carabid glandular secretions include hydrocarbons, cyanide-based molecules (e.g., Formic acid), ketones, quinones, esters, phenols, carboxylic acids, alcohols, monoterpenes, and polypropene [23,24] and other related references are included in Supplementary S1. Using the Pherobase platform (https://pherobase.com/database/family/family-Carabidae.php, accessed on 25 May 2025), common compounds across carabid tribes were identified: Formic acid, Methacrylic acid, Tiglic acid, Ethacrylic acid, Isovaleric acid, Salicylaldehyde, 1,4-benzoquinone, Toluquinone, 13-2Kt (tridecan-2-one), Undecane, Tridecane, Pentadecane, and M-cresol. While some subfamilies produce phenolics like Salicylaldehyde, organic acids are more commonly discovered. Additional compounds such as aromatic esters, aldehydes, ketones, terpenes, and Hydrogen cyanide have also been identified as characteristic glandular chemicals in carabids. Hydroquinones and their oxidized forms, quinones, are prevalent and particularly significant in the Brachininae and Paussinae due to their role in explosive defense. Compared to many other beetle families, the Carabidae are especially rich in glandular substances used for defense against generalist predators and pathogens. In fact, some carabid glandular secretions have demonstrated antimicrobial activity against medically relevant bacteria such as *Bacillus* and *Staphylococcus* species [11,19,112,113]. Additionally, pygidial gland morphology and secretion patterns have been used in taxonomy, phylogenetic analysis, and chemical ecology studies [23,24,26,99]. Geographical and ecological factors also appear to influence secretion composition. For example, tropical carabids tend to produce Formic acid-based secretions, while temperate species often rely on saturated and unsaturated carboxylic acids [23,80,112,114,115]. The paired abdominal exocrine glands in adult carabids show significant diversity across some tribes, and in certain species, the chemicals they produce are sometimes also sexually dimorphic [116,117]. Each gland consists of a collecting canal, a reservoir for storing secretions, and a secretory body composed of multiple lobes [105,112]. The lumen of each secretory lobe is connected to the collecting canal, which leads to the reservoir. From the reservoir, an efferent duct extends, ending in a valve opening and an accessory gland. Among the pygidial glands of ground beetles, those of bombardier beetles are particularly effective against predators [69]. Discharge mechanisms vary among species and include oozing, spraying [16,24], and crepitation, the latter being unique to bombardier beetles (Brachininae) and some Paussidae [65,98,100]. Species of *Brachinus* Weber, 1801, are capable of spraying irritating and hot quinones, produced by rapid hydroquinone oxidation [107,108] (Figure 1h). This exothermic reaction produces both heat and free oxygen, creating a potent deterrent effect [106].

A comparative analysis using Pherobase reveals that ground beetle defensive secretions predominantly include carboxylic acids (e.g., Formic, Methacrylic, and Tiglic acids), aromatic acids (e.g., Benzoic acid), and quinones. These substances are highly effective as a deterrent against predators. Notably, chemical classes commonly found in other beetles, such as pyrazines (frequent in Tenebrionidae) and terpenoids (used by various Cerambycidae) (Supplementary S2 and related references) (https://pherobase.com/database/order/order-Coleoptera.php, accessed on 25 May 2025), are either absent or rarely reported in carabids. In nine beetle families (Boridae, Bostrichidae, Chrysomelidae, Curculionidae, Helodidae, Hydrophilidae, Scarabaeidae, Silphidae, and Staphylinidae), Β-necrodol [((1R,3R)-2,2,3-trimethyl-4-methylenecyclopentyl)methanol] has been identified as a component of their defensive secretions. Cantharidin [2,3-dimethyl-7-oxabicyclo[2.2.1]heptane-2,3-dicarboxylic anhydride] was detected in eight families (Cantharidae, Cerambycidae, Chrysomelidae, Cleridae, Meloidae, Melyridae, Pyrochroidae, and Staphylinidae). The compound 2-sec-butyl-3-methoxypyrazine was identified in six families (Cantharidae, Coccinellidae, Endomychidae, Lycidae, Meloidae, and Pyrochroidae), while Caprylic acid [Octanoic acid] was found in five families (Chrysomelidae, Silphidae, Staphylinidae, Tenebrionidae, and Trachypachidae). Among the coleopteran families known, Chrysomelidae, Coccinellidae, Staphylinidae, Tenebrionidae and Carabidae are the taxa with the highest number of identified defensive chemicals. In addition to some molecules shared across several families, other taxa exhibit exclusive glandular substances, such as Pederin and Pederone in Staphylinidae, Cantharidinimide in Meloidae, Lycidic acid [(E,E)-octadeca-5,7-dien-9-ynoic acid] in Lycidae, and Gyrinidone and Gyrinidione in Gyrinidae.

The difference in the chemical composition of glandular secretions may reflect evolutionary specialization and ecological divergence in chemical defense strategies among beetle families. The chemical classification provided by Pherobase was used as a reference framework to identify and group these compounds. These patterns offer valuable insight into the selective use of defensive chemicals among beetle families and suggest possible evolutionary divergence in chemical defense strategies (see references listed in Supplementary S1 and S2).

## 8. Discussion

Ground beetles employ a diverse array of defensive strategies to protect themselves from predators and pathogens. These include chemical, morphological, and behavioral adaptations, which often function in synergy to enhance survival. Among these, chemical defenses are particularly prominent. The pygidial glands play a key role by producing a variety of noxious compounds, such as carboxylic acids (e.g., Formic, Methacrylic, and Tiglic acids), quinones, phenols, aldehydes, esters, and hydrocarbons. These substances deter predators and, in some cases, exhibit antimicrobial properties. The “chemicals arsenal” of ground beetles varies across tribes, species, sexes, and even development stages. Most studies have focused on adult carabids, while fewer have examined immature stages. For instance, in *Chlaenius cordicollis*, larvae produce defensive compounds distinct from those found in adults, highlighting ontogenetic variation in chemical defense. This variation may influence intraspecific interactions and reflects evolutionary divergence driven by differing ecological niches and predator communities. Carabids, being largely epigean and thus highly exposed to predation, have evolved potent chemical defenses as a primary survival mechanism. The efficiency of these secretions is often enhanced by synergistic interactions among glandular components. For example, in some species, non-polar lipophilic substances facilitate the penetration of polar irritants, such as formic acid, through the predator’s mucosa, thereby amplifying the deterrent effect. However, chemical defenses are energetically expensive to produce and maintain, representing a significant ecological trade-off.

In addition to chemical protection, morphological and behavioral adaptations significantly contribute to survival. Morphological traits such as flattened bodies, large mandibles, and long legs enhance mobility and escape. Behavioral strategies also play a key role in avoiding predation. Many carabids adopt passive strategies, such as hiding in vegetation or soil and seeking sheltered microhabitats (e.g., under stones, in crevices, or beneath leaves). Others exhibit chemical mimicry, aposematism, camouflage, or engage in nocturnal activity and gregarious behavior. Some species also demonstrate alarm–attack signaling systems, where warning colors and chemical emissions create a dilution effect, reducing individual mortality risk. The diversity of defensive mechanisms in Carabidae is shaped by ecological context. Species inhabiting environments with high predation pressure (e.g., tropical forests) tend to rely more heavily on chemical and morphological defenses. In contrast, those in low-predation environments (e.g., temperate regions) may emphasize behavioral strategies such as burrowing or flight. These adaptations contribute significantly to the ecological success of carabids. Despite the wealth of knowledge on adult carabids, larvae and pupae have received less attention. Yet, evidence suggests they possess unique adaptations worthy of further studies. More research is needed on interspecific variation in defensive traits across life stages and how these correlate with ecological niches and predator types (e.g., visual or olfactory predators). Additionally, interspecific and inter-tribe variation in chemical defenses, particularly in comparison to other beetle families, highlights the role of ecological niches and predator diversity in shaping defensive strategies. Understanding the trade-offs between defense types, including resource allocation and fitness costs, remains a crucial area for future investigation. This knowledge is essential not only in the field of evolutionary biology but also for potential applications in pest management and biodiversity conservation.

## 9. Conclusions

Ground beetles exhibit a remarkable array of defensive strategies that underscore their evolutionary success in diverse habitats. Central among these are their sophisticated chemical defenses, which are mediated primarily through the pygidial glands and encompass a complex mix of bioactive compounds. These chemicals, often used synergistically, not only deter a wide range of predators but also provide antimicrobial benefits, revealing a dual function in predator deterrence and pathogen resistance. Morphological and behavioral adaptations further reinforce their defensive repertoire, allowing for flexible responses to varying environmental threats. Ontogenetic and interspecific variation in these defenses, especially between larval and adult stages, highlights the dynamic and context-dependent nature of carabid survival strategies. However, the energetic costs associated with maintaining such diversified defenses suggest important ecological trade-offs that warrant deeper investigation. Despite significant advances, major gaps remain, particularly regarding immature stages and underexplored taxa. Future research focusing on these areas will enhance our understanding of the ecological roles and evolutionary trajectories of ground beetles and may inform conservation and pest management strategies rooted in the principles of chemical ecology.

## Supplementary Materials

The following supporting information can be downloaded at: https://www.mdpi.com/article/10.3390/biology14060709/s1.
