# Supplementary material for: Unveiling the Defenses: A Current and Comprehensive Review of Coleoptera Carabidae Strategies
Source: Biology (Basel). 2025 Jun 17;14(6):709. doi: 10.3390/biology14060709 (PMC12189720; doi:10.3390/biology14060709)
Supplement: Supplementary file 1 [file biology-14-00709-s001.zip › biology-3617803-supplementary.pdf]

Compound	Structure / CAS	Tribe	Carabid species	References
Formic acid	CH2O2/64-18-6	Pterostichini	*Abacomorphus asperulus*	[24]
		Galeritini	*Galerita lecontei*	[334]
		Harpalini	*Acinopus sp*	[268]
		Platynini	*Agonum chalcomum*	[100]
		Platynini	*Agonum chalcomus*	[353]
		Platynini	*Agonum daimio*	[100]
		Platynini	*Agonum duftschiidi*	[315]
		Platynini	*Agonum marginatum*	[315]
		Platynini	*Agonum moestum*	[315]
		Platynini	*Agonum sexpunctatum*	[315]
		Platynini	*Agonum viduum*	[315]
		Amblyterini	*Amblytelus curtus*	[332]
		Sphodrini	*Anchus leucopus*	[100]
		Harpalini	*Anisodactylus binotatus*	[315]
		Harpalini	*Anisodactylus signatus*	[353]
		Harpalini	*Anisodactylus tricuspidatus*	[100]
			Anthia thoracica	[316]
		Lebiini	*Apristus grandis*	[353]
		Lebiini	*Apristus grandis*	[100]
		Odacanthini	*Archiocollius bimaculate nipponica*	[353]
		Licinini	*Badister bipustulatus*	[315]
		Licinini	*Badister bullatus*	[315]
		Harpalini	*Bradycellus inornatus*	[100]
		Sphodrini	*Calathus fuscipes*	[315]
		Sphodrini	*Calathus melanocephalus*	[315]
		Sphodrini	*Calathus ruficollis*	[23]
		Sphodrini	*Calathus sp*	[268]
		Lebiini	*Callida lepida*	[100]
		Harpalini	*Carterus sp*	[268]
		Catapieseini	*Catapiensis attenuata*	[320]
		Odacanthini	*Colliuris pensylvanica*	[320]
		Agonini	*Colpodes atricomes*	[100]
		Agonini	*Colpodes japonicus*	[100]
		Lebiini	*Coptoderina japonica*	[100]

		Lebiini	*Coptoderina subapicalis*	[100]
		Lebiini	*Cymindis daimio*	[353]
		Lebiini	*Demetrida suturalis*	[332]
		Harpalini	*Diachromus germanus*	[315]
		Harpalini	*Diaphoromerus edwardsi*	[325]
		Lebiini	*Dolichoctis luctuosus*	[100]
		Lebiini	*Dolichoctis striata*	[100]
		Lebiini	*Dolichoctis striatus*	[353]
		Lebiini	*Dolichus halensis*	[353]
		Lebiini	*Dolichus halensis*	[100]
		Dryptini	*Drypta australis*	[332]
		Dryptini	*Drypta dentata*	[315]
		Dryptini	*Drypta japonica*	[100-353]
			Eudalia macleayi	[332]
		Galeritini	*Galerita janus*	[334]
		Galeritini	*Galerita japonica*	[100-477]
		Galeritini	*Galerita lecontei*	[334]
		Harpalini	*Harpalus atratus*	[315]
		Harpalini	*Harpalus capito*	[100-353]
		Harpalini	*Harpalus dimidiatus*	[268]
		Harpalini	*Harpalus distinguendus*	[315]
		Harpalini	*Harpalus griseus*	[342-100]
		Harpalini	*Harpalus luteicornis*	[315]
		Harpalini	*Harpalus pensylvanicus*	[341]
		Harpalini	*Harpalus platynotus*	[100]
		Harpalini	*Harpalus pubescens*	[342]
		Harpalini	*Harpalus sinicus*	[100]
		Harpalini	*Harpalus tardus*	[315]
			Helluo costatus	[325]
		Helluonini	*Helluomorphoides clairvillei*	[366]
		Helluonini	*Helluomorphoides ferrugincus*	[343]
		Helluonini	*Helluomorphoides latitarsis*	[343]
		Lebiini	*Lebia chlorocephala*	[315]
		Lebiini	*Lebia retrofasciata*	[100]
		Lebiini	*Lebidia octoguttata*	[100-353]
		Licinini	*Licinus nitidior*	[315]
		Agonini	*Lorostemma ogurae*	[100]
		Loxandrini	*Loxandrus icarus*	[320]
		Loxandrini	*Loxandrus sp*	[320]
		Loxandrini	*Loxandrus velocipes*	[320]
		Agonini	*Loxocrepis rubriola*	[100]

		Pterostichini	*Loxodactylus carinulatus*	[325]
			Mecyclothorax ambiguus	[332]
			Mecyclothorax cordicollis	[332]
		Morionini	*Morion sp*	[320]
			Notagonum submetallicum	[332]
		Odacanthini	*Odacantha melanura*	[315]
		Harpalini	*Ophonus azureus*	[315]
		Loxandrini	*Oxycrepis sp*	[320]
		Lebiini	*Philophloeus australis*	[325]
		Lebiini	*Philophloeus eucalypti*	[332]
		Zuphiini	*Planetes puncticeps*	[100-353]
		Harpalini	*Platymetopus flavilabris*	[100]
		Platynini	*Platynus assimilis*	[315]
		Platynini	*Platynus brunneomarginatus*	[320]
		Platynini	*Platynus dorsalis*	[315]
		Platynini	*Platynus magnus*	[100]
		Platynini	*Platynus ovipennis*	[320]
		Platynini	*Platynus protensus*	[353]
		Zuphiini	*Polysticus connexus*	[315]
		Cyclosomini	*Sarothrocrepis civica*	[332]
		Cyclosomini	*Sarothrocrepis corticallis*	[332]
		Pterostichini	*Sarticus cyaneocinctus*	[325]
		Licinini	*Siagonyx blackburni*	[325]
			Sphallomorpha colymbeioides	[325]
		Pterostichini	*Sphodrosomus saisseti*	[325]
		Harpalini	*Stenolophus difficilis*	[100]
		Harpalini	*Stenolophus iridicolor*	[100]
		Harpalini	*Stenolophus mixtus*	[315]
		Sphodrini	*Synchus callitheres*	[100]
		Sphodrini	*Synchus cycloderus*	[100]
		Sphodrini	*Synchus dulcigradus*	[100-353]
			Thermophilum burchelli	[316]
			Thermophilum homoplatum	[316]
		Harpalini	*Trichocellus tenuimanus*	[100]
		Harpalini	*Trichotichnus longitarsis*	[100-353]
Methacrylic acid	C4H6O2/79-41-4	Pterostichini	*Abacomorphus asperulus*	[24]

		Pterostichini	*Abaris aenea*	[23]
		Pterostichini	*Abax ater*	[205]
		Pterostichini	*Abax ovalis*	[205]
		Carabini, Carabini	*Carabus ullrichii, Calosoma sycophanta*	[320]
		Pterostichini	*Abax parallelepipedus*	[205]
		Amarini	*Amara chalcites*	[100]
		Amarini	*Amara chalcophaea*	[100]
		Amarini	*Amara familiaris*	[205]
		Amarini	*Amara similata*	[205]
		Carabini	*Apotomopterus albr. Esakii*	[268]
		Carabini	*Apotomopterus dehaanii*	[100]
		Carabini	*Apotomopterus insulicula*	[268]
		Carabini	*Apotomopterus japonicus*	[100]
		Carabini	*Apotomopterus yaconinus*	[100]
		Bembidiini	*Bembidion lissonotum*	[100]
		Bembidiini	*Bembidion morawitzi*	[100]
		Bembidiini	*Bembidion stenoderum*	[100]
		Pterostichini	*Blennidus liodes*	[320]
		Bembidiini	*Bembidion semilunium*	[100]
		Amarini	*Bradytus ampliatus*	[100]
		Amarini	*Bradytus simplicidens*	[100]
		Carabini	*Calosoma oceanicum*	[24]
		Carabini	*Calosoma peregrinator*	[326]
		Carabini	*Calosoma schayeri*	[325]
		Carabini	*Calosoma sycophanta*	[323]
		Carabini	*Campalita chinense*	[100]
		Carabini	*Carabus auratus*	[327]
		Carabini	*Carabus autonitens*	[268]
		Carabini	*Carabus caelatus*	[328]
		Carabini	*Carabus cancellatus*	[205]
		Carabini	*Carabus cancellatus*	[205]
		Carabini	*Carabus coriaceus*	[268]
		Carabini	*Carabus cyaneus*	[268]
		Carabini	*Carabus granulatus*	[205]
		Carabini	*Carabus intricatus*	[331]
		Carabini	*Carabus irregularis*	[268]
		Carabini	*Carabus montivagus*	[328]

		Carabini	*Carabus porrecticollis*	[100]
		Carabini	*Carabus problematicus*	[205]
		Carabini	*Carabus procerulus*	[342-100]
		Carabini	*Carabus scheidleri*	[331]
		Carabini	*Carabus taedatus*	[330]
		Carabini	*Carabus ullrichii*	[342-100]
		Carabini	*Carabus violaceus*	[342-100]
		Scaritini	*Carenum bonellii*	[325]
		Scaritini	*Carenum interruptum*	[325]
		Scaritini	*Carenum tinctillatum*	[325]
		Pterostichini	*Castelnaudia superba*	[24]
		Pterostichini	*Catadromus lacordairei*	[332]
		Ceroglossini	*Ceroglossus buqueti*	[59]
		Ceroglossini	*Ceroglossus chilensis*	[59]
		Ceroglossini	*Ceroglossus magellanicus*	[59]
		Oodini	*Coptocarpus modestus*	[332]
		Lebiini	*Coptodera subapicalis*	[353]
		Pterostichini	*Cratoferonia phylarchus*	[325]
		Pterostichini	*Cratogaster melas*	[332]
		Amarini	*Curtonotus giganteus*	[100]
		Cychrini	*Cychrus caraboides*	[89]
		Cychrini	*Cychrus rostaratus*	[268]
		Pterostichini	*Cyclotrachelus sigillatus*	[320]
		Carabini	*Damaster blaptoides*	[100]
		Carabini	*Damaster oxuroides*	[268]
		Dercylini	*Dercylus sp*	[320]
		Patrobini	*Diplous caligatus*	[100]
		Patrobini	*Diplous depressus*	[100]
		Broscini	*Eurylychnus blagravei*	[325]
		Broscini	*Eurylychnus olliffi*	[325]
		Pterostichini	*Gasterllarius honestus*	[320]
		Carabini	*Hemicarabus tuberculosus*	[100]
		Scaritini	*Laccopterum foveigerum*	[325]
		Nebriini	*Leistus ferrugineus*	[205]
		Pterostichini	*Lesticus magnus*	[100]
		Pterostichini	*Molops elatus*	[205]
		Morionini	*Morion sp*	[320]

		Morionini	*Moriosomus seticollis*	[320]
		Pterostichini	*Myas coracinus*	[320]
		Nebriini	*Nebria chinensis*	[100]
		Nebriini	*Nebria lewisi*	[100]
		Nebriini	*Nebria livida*	[205]
		Nebriini	*Nebria macrogona*	[100]
		Nebriini	*Nebria psammodes*	[109]
		Pterostichini	*Nurus nox*	[332]
		Oodini	*Oodes americanus*	[355]
		Oodini	*Oodes modetus*	[332]
		Cychrini	*Pamborus alternans*	[325]
		Cychrini	*Pamborus guerini*	[325]
		Cychrini	*Pamborus pradieri*	[325]
		Cychrini	*Pamborus viridis*	[325]
		Scaritini	*Pasimachus californicus*	[349]
		Scaritini	*Pasimachus duplicatus*	[349]
		Scaritini	*Pasimachus elongatus*	[349]
		Scaritini	*Pasimachus subsulcatus*	[345]
		Patrobini	*Patrobus flavipes*	[100]
		Patrobini	*Patrobus longicornis*	[320]
		Trechini	*Perileptus sp*	[332]
		Scaritini	*Philoscaphus tuberculatus*	[325]
		Pterostichini	*Poecilus coerulescens*	[100]
		Pterostichini	*Poecilus cupreus*	[205]
		Pterostichini	*Poecilus fortipes*	[100]
		Pterostichini	*Prosopogmus harpaloides*	[325]
		Pterostichini	*Pseudabarys sp*	[320]
		Pterostichini	*Pseudoceneus iridescens*	[332]
		Pterostichini	*Pterostichus daisenicus*	[100]
		Pterostichini	*Pterostichus diligendus*	[320]
		Pterostichini	*Pterostichus externepunctatus roccai*	[109]
		Pterostichini	*Pterostichus fortis*	[100]
		Pterostichini	*Pterostichus fujimurai*	[100]
		Pterostichini	*Pterostichus longinquus*	[100]
		Pterostichini	*Pterostichus luctuosus*	[320]
		Pterostichini	*Pterostichus macer*	[205]
		Pterostichini	*Pterostichus masidai*	[100]
		Pterostichini	*Pterostichus melas*	[205]

		Pterostichini	*Pterostichus metallicus*	[205]
		Pterostichini	*Pterostichus microcephalus*	[100]
		Pterostichini	*Pterostichus niger*	[205]
		Pterostichini	*Pterostichus prolangatus*	[100]
		Pterostichini	*Pterostichus vulgaris*	[205]
		Pterostichini	*Rhytisternus laevilaterus*	[325]
		Pterostichini	*Rhytisternus miser*	[332]
		Cychrini	*Scaphinotus andrewsi germari*	[115]
		Cychrini	*Scaphinotus andrewsi montana*	[115]
		Cychrini	*Scaphinotus viduus*	[115]
		Cychrini	*Scaphinotus webbi*	[115]
		Scaritini	*Scarites acutidens*	[100]
		Scaritini	*Scarites aterrimus*	[100]
		Scaritini	*Scarites subterraneus*	[115]
		Scaritini	*Scarites sulcatus*	[100]
		Scaritini	*Scarites terricola*	[100]
		Pterostichini	*Teropha sturti*	[332]
		Pterostichini	*Trichosternus nudipes*	[325]
		Pterostichini	*Trigonognatha cuprescens*	[100]
		Pterostichini	*Trigonotoma lewsii*	[100]
Tiglic acid	C5H8O2/80-59-1	Pterostichini	*Abacomorphus asperulus*	[24]
		Pterostichini	*Abaris aenea*	[23]
		Pterostichini	*Abax ater*	[205]
		Pterostichini	*Abax ovalis*	[205]
		Pterostichini	*Abax parallelepipedus*	[323]
		Pterostichini	*Abax parallelus*	[205]
		Amarini	*Amara chalcites*	[100]
		Carabini	*Carabus ullrichii, Calosoma sycophanta*	[320]
		Amarini	*Amara chalcophaea*	[100]
		Amarini	*Amara familiaris*	[205]
		Amarini	*Amara similata*	[205]
			Anthia thoracica	[316]
		Carabini	*Apotomopterus albr. Esakii*	[268]
		Carabini	*Apotomopterus dehaanii*	[100]
		Carabini	*Apotomopterus japonicus*	[100]
		Carabini	*Apotomopterus insulicula*	[268]
		Carabini	*Apotomopterus yaconinus*	[100]

		Bembidiini	*Bembidion lissonotum*	[100]
		Bembidiini	*Bembidion morawitzi*	[100]
		Bembidiini	*Bembidion semilunium*	[100]
		Bembidiini	*Bembidion stenoderum*	[100]
		Pterostichini	*Blennidus liodes*	[320]
		Amarini	*Bradytus ampliatus*	[100]
		Amarini	*Bradytus simplicidens*	[100]
		Carabini	*Calosoma sycophanta*	[323]
		Carabini	*Campalita chinense*	[100]
		Carabini	*Carabus auratus*	[205]
		Carabini	*Carabus autonitens*	[268]
		Carabini	*Carabus caelatus*	[328]
		Carabini	*Carabus cancellatus*	[205]
		Carabini	*Carabus cancellatus*	[205]
		Carabini	*Carabus coriaceus*	[268]
		Carabini	*Carabus cyaneus*	[268]
		Carabini	*Carabus granulatus*	[205]
		Carabini	*Carabus intricatus*	[331]
		Carabini	*Carabus irregularis*	[268]
		Carabini	*Carabus montivagus*	[328]
		Carabini	*Carabus porrecticollis*	[100]
		Carabini	*Carabus problematicus*	[205]
		Carabini	*Carabus procerulus*	[100]
		Carabini	*Carabus scheidleri*	[331]
		Carabini	*Carabus ullrichii*	[342-100]
		Carabini	*Carabus violaceus*	[342-100]
		Scaritini	*Carenum tinctillatum*	[325]
		Pterostichini	*Castelnaudia superba*	[24]
		Pterostichini	*Catadromus lacordairei*	[332]
		Ceroglossini	*Ceroglossus buqueti*	[59]
		Ceroglossini	*Ceroglossus chilensis*	[59]
		Oodini	*Coptocarpus modestus*	[332]
		Pterostichini	*Cratoferonia phylarchus*	[325]
		Pterostichini	*Cratogaster melas*	[332]
		Amarini	*Curtonotus giganteus*	[100]
		Carabini	*Damaster blaptoides*	[100]
		Dercylini	*Dercylus sp*	[320]
		Patrobini	*Diplous caligatus*	[100]
		Patrobini	*Diplous depressus*	[100]

		Broscini	*Eurylychnus blagravei*	[325]
		Broscini	*Eurylychnus olliffi*	[325]
		Pterostichini	*Incastichus aequidianus*	[320]
		Scaritini	*Laccopterum foveigerum*	[325]
		Nebriini	*Leistus ferrugineus*	[205]
		Nebriini	*Lesticus magnus*	[100]
		Pterostichini	*Molops elatus*	[205]
		Pterostichini	*Myas coracinus*	[320]
		Nebriini	*Nebria chinensis*	[100]
		Nebriini	*Nebria lewisi*	[100]
		Nebriini	*Nebria livida*	[205]
		Nebriini	*Nebria macrogona*	[100]
		Nebriini	*Nebria psammodes*	[108]
		Pterostichini	*Nurus nox*	[332]
		Oodini	*Oodes americanus*	[355]
		Oodini	*Oodes modetus*	[332]
		Scaritini	*Pasimachus subsulcatus*	[345]
		Patrobini	*Patrobus longicornis*	[320]
		Trechini	*Perileptus sp*	[332]
		Scaritini	*Philoscaphus tuberculatus*	[325]
		Pterostichini	*Poecilus coerulescens*	[100]
		Pterostichini	*Poecilus cupreus*	[205]
		Pterostichini	*Poecilus fortipes*	[100]
		Pterostichini	*Pseudabarys sp*	[320]
		Pterostichini	*Pseudoceneus iridescens*	[332]
		Pterostichini	*Pterostichus daisenicus*	[100]
		Pterostichini	*Pterostichus diligendus*	[320]
		Pterostichini	*Pterostichus externepunctatus roccai*	[108]
		Pterostichini	*Pterostichus fortis*	[100]
		Pterostichini	*Pterostichus fujimurai*	[100]
		Pterostichini	*Pterostichus longinquus*	[100]
		Pterostichini	*Pterostichus luctuosus*	[320]
		Pterostichini	*Pterostichus macer*	[205]
		Pterostichini	*Pterostichus masidai*	[100]
		Pterostichini	*Pterostichus melas*	[205]
		Pterostichini	*Pterostichus metallicus*	[205]
		Pterostichini	*Pterostichus microcephalus*	[100]
		Pterostichini	*Pterostichus niger*	[205]

		Pterostichini	*Pterostichus prolangatus*	[100]
		Pterostichini	*Pterostichus rotundangulus*	[100]
		Pterostichini	*Pterostichus vulgaris*	[205]
		Pterostichini	*Rhytisternus laevilaterus*	[325]
		Pterostichini	*Rhytisternus miser*	[332]
		Cychrini	*Scaphinotus andrewsi germari*	[115]
		Cychrini	*Scaphinotus andrewsi montana*	[115]
		Cychrini	*Scaphinotus viduus*	[115]
		Cychrini	*Scaphinotus webbi*	[115]
			Scarites acutidens	[100]
		Scaritini	*Scarites aterrimus*	[100]
		Scaritini	*Scarites subterraneus*	[115]
		Scaritini	*Scarites terricola*	[100]
		Pterostichini	*Teropha sturti*	[332]
			Thermophilum burchelli	[316]
			Thermophilum homoplatum	[316]
		Pterostichini	*Trichosternus nudipes*	[325]
		Pterostichini	*Trigonognatha cuprescens*	[100]
		Pterostichini	*Trigonotoma lewsii*	[100]
Acetic acid	C2H4O2/64-19-7	Amarini	*Amara chalcites*	[100]
		Amarini	*Amara chalcophaea*	[100]
		Amarini	*Anthia thoracica*	[316]
		Amarini	*Bradytus ampliatus*	[100]
		Amarini	*Bradytus simplicidens*	[100]
		Carabini	*Carabus caelatus*	[328]
		Pterostichini	*Castelnaudia superba*	[24]
		Odacanthini	*Colliuris pensylvanica*	[320]
		Amarini	*Curtonotus giganteus*	[100]
		Pterostichini	*Cyclotrachelus sigillatus*	[320]
		Helluonini	*Helluomorphoides clairvillei*	[ND]
		Loxandrini	*Loxandrus icarus*	[320]
		Loxandrini	*Loxandrus sp*	[320]
		Loxandrini	*Loxandrus sp*	[320]
		Morionini	*Morion sp*	[320]
		Morionini	*Moriosomus seticollis*	[320]
		Loxandrini	*Oxycrepis sp*	[320]
		Galeritini	*Galerita lecontei*	[334]

		Ceroglossini	*Ceroglossus buqueti*	[59]
		Oodini	*Oodes amaroides*	[320]
		Oodini	*Oodes americanus*	[355]
		Patrobini	*Patrobus longicornis*	[320]
			Thermophilum homoplatum	[316]
			Thermophilum burchelli	[316]
Angelic acid	C5H8O2/565-63-9	Pterostichini	*Abacomorphus asperulus*	[24]
		Carabini	*Carabus caelatus*	[328]
		Carabini	*Carabus ullrichii*	[323]
		Carabini	*Carabus violaceus*	[328]
		Scaritini	*Carenum bonellii*	[325]
		Scaritini	*Carenum interruptum*	[325]
		Scaritini	*Carenum tinctillatum*	[325]
		Broscini	*Craspedonotus tibialis*	[100]
		Scaritini	*Pasimachus subsulcatus*	[345]
		Patrobini	*Patrobus flavipes*	[100]
		Scaritini	*Scarites acutidens*	[100]
		Scaritini	*Scarites aterrimus*	[100]
		Scaritini	*Scarites subterraneus*	[115]
		Scaritini	*Scarites sulcatus*	[100]
		Scaritini	*Scarites sulcatus*	[100]
		Scaritini	*Scarites terricola*	[100]
Ethacrilic acid	C5H8O2/3586-58-1	Amarini	*Amara chalcites*	[100]
		Amarini	*Amara chalcophaea*	[100]
		Carabini	*Apotomopterus dehaanii*	[100]
		Carabini	*Apotomopterus japonicus*	[100]
		Carabini	*Apotomopterus yaconinus*	[100]
		Amarini	*Bradytus ampliatus*	[100]
		Amarini	*Bradytus simplicidens*	[100]
		Carabini	*Carabus porrecticollis*	[100]
		Carabini	*Carabus procerulus*	[100]
		Carabini	*Carabus taedatus*	[330]
		Ceroglossini	*Ceroglossus buqueti*	[59]
		Ceroglossini	*Ceroglossus chilensis*	[59]
		Ceroglossini	*Ceroglossus magellanicus*	[59]
		Broscini	*Craspedonotus tibialis*	[100]
		Amarini	*Curtonotus giganteus*	[100]

		Carabini	*Damaster blaptoides*	[100]
		Carabini	*Hemicarabus tuberculosus*	[100]
		Scaritini	*Laccopterum foveigerum*	[325]
		Nebriini	*Nebria chinensis*	[100]
		Nebriini	*Nebria lewisi*	[100]
		Nebriini	*Nebria macrogona*	[100]
		Cychrini	*Pamborus alternans*	[325]
		Cychrini	*Pamborus guerini*	[325]
		Cychrini	*Pamborus pradieri*	[325]
		Cychrini	*Pamborus viridis*	[325]
		Patrobini	*Patrobus flavipes*	[100]
		Pterostichini	*Pterostichus daisenicus*	[100]
		Pterostichini	*Pterostichus fortis*	[100]
		Pterostichini	*Pterostichus fujimurai*	[100]
		Pterostichini	*Pterostichus longinquus*	[100]
		Pterostichini	*Pterostichus masidai*	[100]
		Pterostichini	*Pterostichus microcephalus*	[100]
		Pterostichini	*Pterostichus prolangatus*	[100]
		Pterostichini	*Pterostichus rotundangulus*	[100]
Benzoic acid	C7H6O2/65-85-0	Carabini	*Calosoma sycophanta*	[323]
		Carabini	*Carabus caelatus*	[328]
		Carabini	*Carabus ullrichii*	[323]
		Carabini	*Carabus violaceus*	[328]
		Ceroglossini	*Ceroglossus buqueti*	[59]
		Ceroglossini	*Ceroglossus chilensis*	[59]
		Ceroglossini	*Ceroglossus magellanicus*	[59]
		Morionini	*Moriosomus seticollis*	[320]
		Oodini	*Oodes americanus*	[355]
Nitrous acid	HNO2/7782-77-6	Brachinini	*Pheropsophus africanus*	[352]
Acid [Propanoic acid]	C3H6O2/79-09-4	Ceroglossini	*Ceroglossus buqueti*	[59]
		Oodini	*Oodes americanus*	[355]
4Acid [Butyric acid]	C4H8O2/107-92-6	Bembidiini	*Asaphidion semilucidum*	[100]
		Broscini	*Broscosoma doenitzi*	[100]
		Carabini	*Calosoma sycophanta*	[323]
		Carabini	*Carabus caelatus*	[328]
		Carabini	*Carabus ullrichii*	[323]
		Ceroglossini	*Ceroglossus buqueti*	[59]
		Ceroglossini	*Ceroglossus chilensis*	[59]

		Ceroglossini	*Ceroglossus magellanicus*	[59]
		Oodini	*Oodes americanus*	[355]
		Broscini	*Promecoderus mastersi*	[332]
		Broscini	*Promecoderus sp*	[325]
Isovaleric acid	C5H10O2/503-74-2	Bembidiini	*Asaphidion semilucidum*	[100]
		Omophronini	*Omophron limbatum*	[315]
		Bembidiini	*Bembidion andreae*	[315]
		Bembidiini	*Bembidion lampros*	[315]
		Broscini	*Broscosoma doenitzi*	[100]
		Broscini	*Broscus cephalotes*	[315]
		Ceroglossini	*Ceroglossus buqueti*	[59]
		Ceroglossini	*Ceroglossus chilensis*	[59]
		Broscini	*Craspedonotus tibialis*	[100]
		Elaphrini	*Elaphrus ripareus*	[315]
		Broscini	*Eurylychnus olliffi*	[325]
		Lorocerini	*Lorocera pilicornis*	[315]
		Oodini	*Oodes amaroides*	[320]
		Oodini	*Oodes americanus*	[355]
		Scaritini	*Pasimachus subsulcatus*	[345]
		Broscini	*Promecoderus mastersi*	[332]
		Broscini	*Promecoderus sp*	[325]
		Pentagonicini	*Scopodes boops*	[332]
		Trechini	*Tachys sericans*	[100]
		Trechini	*Trechoblemus postilenatus*	[100]
Isobutyric acid [2-Methylpropanoic acid]	C4H8=2/79-31-2	Bembidiini	*Bembidion andreae*	[315]
		Bembidiini	*Bembidion lampros*	[315]
		Broscini	*Broscus cephalotes*	[315]
		Carabini	*Calosoma sycophanta*	[323]
		Pterostichini	*Abax parallelepipedus*	[323]
		Oodini	*Oodes americanus*	[355]
		Scaritini	*Scarites subterraneus*	[115]
		Carabini	*Carabus caelatus*	[328]
		Carabini	*Carabus ullrichii*	[323]
		Carabini	*Carabus violaceus*	[328]
		Ceroglossini	*Ceroglossus buqueti*	[59]
		Ceroglossini	*Ceroglossus chilensis*	[59]
		Ceroglossini	*Ceroglossus magellanicus*	[59]
		Broscini	*Craspedonotus tibialis*	[100]
		Elaphrini	*Elaphrus ripareus*	[315]

		Lorocerini	*Lorocera pilicornis*	[315]
		Omophronini	*Omophron limbatum*	[315]
		Oodini	*Oodes amaroides*	[320]
		Scaritini	*Pasimachus subsulcatus*	[345]
		Broscini	*Promecoderus mastersi*	[332]
		Pterostichini	*Pterostichus externepunctatus roccai*	[109]
		Trechini	*Tachys sericans*	[100]
		Trechini	*Trechoblemus postilenatus*	[100]
Caproic acid [Hexanoic acid]	C6H12O2/14262-1	Carabini	*Calosoma oceanicum*	[24]
		Carabini	*Calosoma schayeri*	[325]
		Scaritini	*Laccopterum foveigerum*	[325]
		Oodini	*Oodes americanus*	[355]
		Broscini	*Promecoderus mastersi*	[332]
		Broscini	*Promecoderus sp*	[325]
Isohexanoic acid [4-Methylpentanoic acid]	C6H12O2/646-07-1	Scaritini	*Philoscaphus tuberculatus*	[325]
2me-4Acid [2-Methylbutanoic acid]	C5H10O2/116-53-0	Carabini	*Calosoma sycophanta*	[323]
		Carabini	*Carabus caelatus*	[328]
		Carabini	*Carabus ullrichii*	[323]
		Carabini	*Carabus violaceus*	[328]
		Broscini	*Eurylychnus olliffi*	[325]
		Oodini	*Oodes americanus*	[355]
		Scaritini	*Pasimachus subsulcatus*	[345]
		Broscini	*Promecoderus sp*	[325]
E2-4Acid [(E)-2-Butenoic acid]	C4H6O2/107-93-7	Pterostichini	*Abax parallelepipedus*	[323]
		Carabini	*Calosoma sycophanta*	[323]
		Scaritini	*Laccopterum foveigerum*	[325]
		Oodini	*Oodes americanus*	[355]
		Patrobini	*Patrobus flavipes*	[100]
		Scaritini	*Philoscaphus tuberculatus*	[325]
		Scaritini	*Scarites subterraneus*	[115]
		Pterostichini	*Teropha sturti*	[332]
E2-6Acid [(E)-2-Hexenoic acid]	C6H10O2/13419-69-7	Scaritini	*Carenum bonellii*	[325]
		Scaritini	*Laccopterum foveigerum*	[325]
		Oodini	*Oodes americanus*	[355]
E2-8Acid [(E)-2-Octenoic acid]	C8H14O2/1871-67-6	Oodini	*Oodes americanus*	[355]
Delta2-6Acid [2-Hexenoic acid]	C6H10O2/1191-04-4	Carabini	*Carabus caelatus*	[328]

		Clivinini	*Schizogenius lineolatus*	[320]
Delta3-6Acid [3-Hexenoic acid]	C6H10O2/1577-18-0	Clivinini	*Schizogenius lineolatus*	[320]
Delta3,5-6Acid [3,5-Hexadienoic acid]	C6H8O2/32775-95-4	Clivinini	*Schizogenius lineolatus*	[320]
Z2-4Acid [(Z)-2-Butenoic acid]	C4H6O2/503-64-0	Scaritini	*Carenum bonellii*	[325]
		Scaritini	*Carenum interruptum*	[325]
		Scaritini	*Carenum tinctillatum*	[325]
		Scaritini	*Laccopterum foveigerum*	[325]
		Patrobini	*Patrobus flavipes*	[100]
		Scaritini	*Philoscaphus tuberculatus*	[325]
		Scaritini	*Scarites acutidens*	[100]
		Scaritini	*Scarites aterrimus*	[100]
		Scaritini	*Scarites subterraneus*	[115]
		Scaritini	*Scarites sulcatus*	[100]
		Scaritini	*Scarites terricola*	[100]
Isovaleraldehyde [3-Methylbutanal]	C5H10O/590-86-3		*Anthia thoracica*	[316]
			Thermophilum homoplatum	[316]
Salicylaldehyde [2-Hydroxybenzaldehyde]	C7H6O2 / 90-02-8	Bembidiini	*Asaphidion flavipes*	[315]
		Bembidiini	*Bembidion quadriguttatum*	[315]
		Carabini	*Calosoma affine*	[326]
		Carabini	*Calosoma alternans sayi*	[326]
		Carabini	*Calosoma macrum*	[326]
		Carabini	*Calosoma oceanicum*	[24]
		Carabini	*Calosoma parvicollis*	[326]
		Carabini	*Calosoma peregrinator*	[326]
		Carabini	*Calosoma prominens*	[368]
		Carabini	*Calosoma schayeri*	[325]
		Carabini	*Calosoma sycophanta*	[323]
		Carabini	*Campalita chinense*	[100]
		Loxandrini	*Loxandrus longisformis*	[332]
		Loxandrini	*Loxandrus sp*	[325]
Methyl salicylate	C8H8O3/119-36-8	Platynini	*Platynus dorsalis*	[315]
1,4-Benzoquinone	C6H4O2 / 106-51-4	Clivinini	*Ardistomis sp*	[367]
		Brachinini	*Brachinus spp.*	[1]
		Brachinini	*Brachinus chuji*	[100]
		Brachinini	*Brachinus crepitans*	[322]
		Brachinini	*Brachinus explodens*	[351]
		Brachinini	*Brachinus quadripennis*	[318]

		Brachinini	*Brachinus sclopeta*	[351]
		Brachinini	*Brachinus scotomedes*	[100]
		Brachinini	*Brachinus stenoderus*	[100]
		Brachinini	*Brachinus sublaevis*	[318]
		Chlaeniini	*Callistus lunatus*	[315]
		Chlaeniini	*Chlaenius inops*	[100]
		Chlaeniini	*Chlaenius prostenus*	[100]
		Chlaeniini	*Chlaenius vestitus*	[109]
		Clivinini	*Clivina basalis*	[325]
		Clivinini	*Clivina sp*	[100]
		Crepidogastrini	*Crepidogaster ambreana*	[104]
		Crepidogastrini	*Crepidogaster atrata*	[104]
		Ozaenini	*Goniotropis nicaraguensis*	[318]
		Metriini	*Metrius contractus*	[318]
		Mystropomini	*Mystropomus regularis*	[338]
		Ozaenini	*Ozaena magna*	[318]
		Ozaenini	*Pachyteles sp*	[344]
		Paussini	*Paussus favieri*	[350]
		Brachinini	*Pheropsophus catoieri*	[351]
		Brachinini	*Pheropsophus jessoensis*	[100]
		Brachinini	*Pheropsophus verticalis*	[325]
		Ozaenini	*Physea hirta*	[344]
		Ozaenini	*Platycerozaena panamensis*	[344]
		Ozaenini	*Pseudozaena orinetalis opaca*	[346]
		Clivinini	*Semiardistomis puncticollis*	[367]
		Metriini	*Stenaptinus insignis*	[318]
Toluquinone [2-Methyl-1,4-benzochinone]	C7H6O2 / 553-97-9	Brachinini	*Aptinus pyrenaeus*	[315]
		Clivinini	*Ardistomis schaumii*	[367]
		Clivinini	*Ardistomis sp*	[367]
		Paussini	*Arthropterus sp*	[325]
		Brachinini	*Brachinus chuji*	[100]
		Brachinini	*Brachinus crepitans*	[322]
		Brachinini	*Brachinus explodens*	[351]
		Brachinini	*Brachinus quadripennis*	[318]
		Brachinini	*Brachinus sclopeta*	[351]
		Brachinini	*Brachinus scotomedes*	[100]
		Brachinini	*Brachinus stenoderus*	[100]
		Brachinini	*Brachinus sublaevis*	[318]
		Chlaeniini	*Chlaenius inops*	[100]

		Chlaeniini	*Chlaenius prostenus*	[100]
		Chlaeniini	*Chlaenius vestitus*	[109]
		Clivinini	*Clivina basalis*	[325]
		Clivinini	*Clivina sp*	[100]
		Crepidogastrini	*Crepidogaster ambreana*	[104]
		Crepidogastrini	*Crepidogaster atrata*	[104]
		Ozaenini	*Goniotropis nicaraguensis*	[318]
		Paussini	*Homopterus arrowi*	[318]
		Mystropomini	*Mystropomus regularis*	[338]
		Ozaenini	*Ozaena magna*	[318]
		Ozaenini	*Pachyteles sp*	[344]
		Paussini	*Paussus favieri*	[350]
		Brachinini	*Pheropsophus catoieri*	[351]
		Brachinini	*Pheropsophus jessoensis*	[100]
		Brachinini	*Pheropsophus verticalis*	[325]
		Ozaenini	*Physea hirta*	[344]
		Ozaenini	*Platycerozaena panamensis*	[344]
		Ozaenini	*Pseudozaena orinetalis opaca*	[346]
		Clivinini	*Semiardistomis puncticollis*	[367]
		Metriini	*Stenaptinus insignis*	[318]
2-Methoxy-3-methyl-1,4-benzoquinone	C8H8O3 / 2207-57-0	Brachinini	*Brachinus*	[322]
		Brachinini	*Brachinus quadripennis*	[318]
		Chlaeniini	*Chlaenius vestitus*	[109]
		Clivinini	*Clivina fossor*	[315]
		Ozaenini	*Goniotropis nicaraguensis*	[318]
		Ozaenini	*Ozaena magna*	[318]
		Ozaenini	*Pachyteles sp*	[344]
		Ozaenini	*Physea hirta*	[344]
		Ozaenini	*Platycerozaena panamensis*	[344]
2-Ethyl-1,4-benzoquinone	C8H8O2/4754-26-1	Paussini	*Arthropterus sp*	[325]
		Mystropomini	*Mystropomus regularis*	[338]
		Ozaenini	*Platycerozaena panamensis*	[344]
2-Methoxy-3methyl-1,4-benzoquinone	C8H8O3/2207-57-0	Paussini	*Homopterus arrowi*	[318]
Senecioic acid [3-Methyl-2-buteoic acid]	C5H8O2/541-47-9	Pterostichini	*Abax parallelepipedus*	[323]
		Pterostichini	*Incastichus aequidianus*	[320]
		Carabini	*Calosoma sycophanta*	[323]
		Carabini	*Carabus caelatus*	[328]

		Carabini	*Carabus violaceus*	[328]
		Pterostichini	*Gasterllarius honestus*	[320]
		Morionini	*Morion sp*	[320]
		Pterostichini	*Teropha sturti*	[332]
		Pterostichini	*Myas coracinus*	[320]
		Scaritini	*Pasimachus subsulcatus*	[345]
13-2Kt [Tridecan-2-one]	C13H26O/593-08-8	Platynini	*Agonum chalcomus*	[353]
		Amblytelini	*Amblytelus curtus*	[332]
		Harpalini	*Anisodactylus signatus*	[353]
		Lebiini	*Apristus grandis*	[353]
		Odacanthini	*Archiocollius bimaculate nipponica*	[353]
		Lebiini	*Coptodera subapicalis*	[353]
		Lebiini	*Cymindis daimio*	[353]
		Lebiini	*Dolichoctis striatus*	[353]
		Platynini	*Dolichus halensis*	[353]
		Harpalini	*Harpalus capito*	[353]
		Harpalini	*Harpalus pensylvanicus*	[341]
		Lebiini	*Lebidia octoguttata*	[353]
			Notagonum submetallicum	[332]
		Cyclosomini	*Sarothrocrepis civica*	[332]
		Cyclosomini	*Sarothrocrepis corticallis*	[332]
		Pentagonicini	*Scopodes boops*	[332]
		Harpalini	*Stenolophus agonoides*	[353]
		Sphodrini	*Synchus dulcigradus*	[353]
		Harpalini	*Trichotichnus longitarsis*	[353]
9Hy [Nonane]	C9H2O/11-84-2	Catapieseini	*Catapiensis attenuata*	[320]
		Galeritini	*Galerita lecontei*	[334]
		Cyclosomini	*Sarothrocrepis corticallis*	[332]
10Hy [Decane]	C10H22/124-18-5	Amarini	*Amara familiaris*	[205]
		Amarini	*Amara similata*	[205]
		Catapieseini	*Catapiensis attenuata*	[320]
		Helluonini	*Helluomorphoides clairvillei*	[366]
		Loxandrini	*Loxandrus icarus*	[320]
		Loxandrini	*Loxandrus sp*	[320]
		Loxandrini	*Oxycrepis sp*	[320]
		Pterostichini	*Poecilus cupreus*	[205]
		Pterostichini	*Pseudabarys sp*	[320]
		Pterostichini	*Pterostichus macer*	[205]

		Pterostichini	*Pterostichus melas*	[205]
		Pterostichini	*Pterostichus metallicus*	[205]
		Pterostichini	*Pterostichus niger*	[205]
		Pterostichini	*Pterostichus vulgaris*	[205]
11Hy [Undecane]	C11H24/1120-21-4	Amarini	*Amara familiaris*	[205]
		Amarini	*Amara similata*	[205]
		Amblytelini	*Amblytelus curtus*	[332]
		Platynini	*Anchomenus dorsalis*	[56]
		Catapieseini	*Catapiensis attenuata*	[320]
		Odacanthini	*Colliuris pensylvanica*	[320]
		Dryptini	*Drypta australis*	[332]
		Galeritini	*Galerita lecontei*	[334]
		Harpalini	*Harpalus pensylvanicus*	[341]
		Pterostichini	*Incastichus aequidianus*	[320]
		Loxandrini	*Loxandrus icarus*	[320]
		Loxandrini	*Loxandrus longisformis*	[332]
		Loxandrini	*Loxandrus sp*	[320]
		Loxandrini	*Loxandrus sp*	[320]
		Morionini	*Morion sp*	[320]
		Morionini	*Moriosomus seticollis*	[320]
			Notagonum submetallicum	[332]
		Loxandrini	*Oxycrepis sp*	[320]
		Pterostichini	*Poecilus cupreus*	[205]
		Pterostichini	*Pseudabarys sp*	[320]
		Pterostichini	*Pterostichus externepunctatus roccai*	[108]
		Pterostichini	*Pterostichus macer*	[205]
		Pterostichini	*Pterostichus melas*	[205]
		Pterostichini	*Pterostichus metallicus*	[205]
		Pterostichini	*Pterostichus niger*	[205]
		Pterostichini	*Pterostichus vulgaris*	[205]
		Cyclosomini	*Sarothrocrepis civica*	[332]
		Pentagonicini	*Scopodes boops*	[332]
12Hy [Dodecane]	C12H26/112-40-3	Pterostichini	*Abaris aenea*	[23]
		Morionini	*Morion sp*	[320]
		Pterostichini	*Pseudabarys sp*	[320]
13Hy [Tridecane]	C13H29/629-50-5	Pterostichini	*Abaris aenea*	[23]
		Amarini	*Amara familiaris*	[205]
		Amarini	*Amara similata*	[205]
		Panagaeini	*Craspedophorus sp*	[325]

			Mecyclothorax ambiguus	[332]
			Mecyclothorax cordicollis	[332]
		Metriini	*Metrius contractus*	[318]
		Morionini	*Morion sp*	[320]
		Morionini	*Moriosomus seticollis*	[320]
		Ozaenini	*Pachyteles sp*	[344]
		Trechini	*Perileptus sp*	[332]
		Pterostichini	*Poecilus cupreus*	[205]
		Pterostichini	*Pseudabarys sp*	[320]
		Pterostichini	*Pseudoceneus iridescens*	[332]
		Pterostichini	*Pterostichus externepunctatus roccai*	[108]
		Pterostichini	*Pterostichus macer*	[205]
		Pterostichini	*Pterostichus melas*	[205]
		Pterostichini	*Pterostichus metallicus*	[205]
		Pterostichini	*Pterostichus niger*	[205]
		Pterostichini	*Pterostichus vulgaris*	[205]
		Pterostichini	*Rhytisternus miser*	[332]
14Hy [Tetradecane]	C14H30/629-59-4	Ozaenini	*Goniotropis nicaraguensis*	[318]
		Metriini	*Metrius contractus*	[318]
		Ozaenini	*Pachyteles sp*	[344]
15Hy [Pentadecane]	C15H32/629-62-9	Brachinini	*Brachinus quadripennis*	[318]
		Chlaeniini	*Chlaenius greyanus*	[332]
		Pterostichini	*Cratogaster melas*	[332]
		Crepidogastrini	*Crepidogaster ambreana*	[104]
		Crepidogastrini	*Crepidogaster atrata*	[104]
			Eudalia macleayi	[332]
		Ozaenini	*Goniotropis nicaraguensis*	[337]
		Ozaenini	*Goniotropis nicaraguensis*	[318]
		Metriini	*Metrius contractus*	[318]
		Morionini	*Morion sp*	[320]
		Morionini	*Moriosomus seticollis*	[320]
		Mystropomini	*Mystropomus regularis*	[338]
		Ozaenini	*Pseudozaena orinetalis opaca*	[346]
16Hy [Hexadecane]	C16H34/544-76-3	Metriini	*Metrius contractus*	[102]
17Hy [Heptadecane]	C17H36/629-78-7	Chlaeniini	*Chlaenius velutinus*	[108]
		Pterostichini	*Cratogaster melas*	[332]
			Eudalia macleayi	[332]
		Metriini	*Metrius contractus*	[102]

		Morionini	*Moriosomus seticollis*	[320]
		Pterostichini	*Pseudabarys sp*	[320]
19Hy [Nonadecane]	C19H40/629-92-5	Pterostichini	*Pseudabarys sp*	[320]
9Ac [Nonyl acetate]	C11H22O2/143-13-5	Dryptini	*Drypta japonica*	[353]
		Galeritini	*Galerita japonica*	[353]
		Galeritini	*Galerita lecontei*	[334]
			Helluo costatus	[325]
		Helluonini	*Helluomorphoides clairvillei*	[366]
		Helluonini	*Helluomorphoides ferrugincus*	[343]
		Helluonini	*Helluomorphoides latitarsis*	[343]
		Zuphiini	*Planetes puncticeps*	[353]
10Ac [Decyl acetate]	C12H24O2/112-17-4	Catapieseini	*Catapiensis attenuata*	[320]
		Dryptini	*Drypta australis*	[332]
		Dryptini	*Drypta japonica*	[353]
		Galeritini	*Galerita japonica*	[353]
		Galeritini	*Galerita lecontei*	[334]
		Zuphiini	*Planetes puncticeps*	[353]
11Ac [Undecyl acetate]	C13H26O2/1731-81-3	Morionini	*Moriosomus seticollis*	[320]
12Ac [Dodecyl acetate]	C14H28O2/112-66-3	Amblytelini	*Amblytelus curtus*	[332]
		Morionini	*Moriosomus seticollis*	[320]
13Ac [Tridecyl acetate]	C15H30O2/1072-33-9	Morionini	*Moriosomus seticollis*	[320]
14Ac [Tetradecyl acetate]	C16H32O2/638-59-5		*Mecyclothorax cordicollis*	[332]
		Morionini	*Moriosomus seticollis*	[320]
21Hy [Heneicosane]	C21H44/629-94-7	Platynini	*Anchomenus dorsalis*	[56]
Z6Z9-17Hy [(Z,Z)-6,9-Heptadecadiene]	C17H32/unvailable	Metriini	*Metrius contractus*	[102]
Z7Z9-17Hy [(Z,Z)-7,9-Heptadecadiene]	C17H32/unvailable	Metriini	*Metrius contractus*	[102]
Z8-17hy [(Z)-8-Heptadecene]	C17H34/16369-12-3	Metriini	*Metrius contractus*	[102]
Z9-23Hy [(Z)-9-Tricosene]	C23H46/27519-02-4	Platynini	*Anchomenus dorsalis*	[56]
Delta5,7-15Hy [Pentadeca-5,7-diene]	C15H28/unvailable	Metriini	*Metrius contractus*	[102]
Delta5,7-17Hy [Heptadeca-5,7-diene]	C17H32/unvailable	Metriini	*Metrius contractus*	[102]
Delta7,9-17Hy [Heptadeca-7,9-diene]	C17H32/unvailable	Metriini	*Metrius contractus*	[102]
Delta7-15Hy [7-Pentadecene]	C15H30/unvailable	Chlaeniini	*Chlaenius australis*	[332]
		Chlaeniini	*Chlaenius greyanus*	[332]
Delta6,9-17Hy [Heptadeca-6,9-diene]	C17H32/81265-03-4	Chlaeniini	*Chlaenius greyanus*	[332]
Delta8-17Hy [8-Heptadecene]	C17H34/2579-04-6	Chlaeniini	*Chlaenius australis*	[332]
		Chlaeniini	*Chlaenius greyanus*	[332]
		Pterostichini	*Pseudabarys sp*	[320]

Delta9-19Hy [9-Nonadecene]	C19H38/31035-07-1	Pterostichini	*Pseudabarys sp*	[320]
Delta11-23Hy [11-Tricosene]	C23H46/52078-56-5	Ceroglossini	*Ceroglossus buqueti*	[59]
		Ceroglossini	*Cerglossus chilensis*	[59]
3me-15Hy [3-Methylpentadecane]	C16H34/2882-96-4	Metriini	*Metrius contractus*	[102]
5me9me-15Hy [5,9-Dimethylpentadecane]	C17H36/unvailable	Chlaeniini	*Chlaenius velutinus*	[108]
Beta-necrodol [(1R,3R)-2,2,3-Trmethyl-4-methylenecyclopentyl)-methanol]	C10H18O/unvailable		*Anatrichus minutus*	[188]
		Clivinini	*Clivina sp*	[188]
		Carabini	*Pentagonica picticornis*	[188]
		Harpalini	*Stenolophus sp*	[188]
Toluene	C7H8/108-88-3	Platynini	*Anchomenus dorsalis*	[56]
O-cresol [2-Methylphenol]	C7H80/95-48-7	Platynini	*Anchomenus dorsalis*	[56]
M-cresol [3-Methylphenol]	C7H80/108-39-4	Chlaeniini	*Callistoides delciolus*	[100]
		Chlaeniini	*Chlaenius australis*	[332]
		Chlaeniini	*Chlaenius chrysocephalus*	[315]
		Chlaeniini	*Chlaenius circumdatus*	[100]
		Chlaeniini	*Chlaenius cordicollis*	[293-369]
		Chlaeniini	*Chlaenius darlingensis*	[332]
		Chlaeniini	*Chlaenius festivus*	[315]
		Chlaeniini	*Chlaenius greyanus*	[332]
		Chlaeniini	*Chlaenius noguchii*	[100]
		Chlaeniini	*Chlaenius pallipes*	[100]
		Chlaeniini	*Chlaenius spoliatus*	[100]
		Chlaeniini	*Chlaenius tristis*	[315]
		Chlaeniini	*Chlaenius velutinus*	[108]
		Chlaeniini	*Chlaenius virgulifer*	[100]
		Panagaeini	*Craspedophorus australasiae*	[332]
		Panagaeini	*Craspedophorus insignis*	[332]
		Panagaeini	*Craspedophorus mastersi*	[332]
		Panagaeini	*Craspedophorus rockhamptonensis*	[332]
		Panagaeini	*Craspedophorus sp*	[325]
		Panagaeini	*Dischissus japonicus*	[100]
		Panagaeini	*Dischissus mirandus*	[100]
		Chlaeniini	*Epomis nigricans*	[100]
		Chlaeniini	*Macrochlaenites costiger*	[100]
			Panagaeus japonicus	[100]
		Panagaeini	*Peronomerus auripillis*	[100]
		Panagaeini	*Peronomerus nigrinus*	[100]

		Panagaeini	*Trichisia azurea*	[332]
p-cresol [4-Methylphenol]	C7H80/106-44-5	Chlaeniini	*Chlaenius cordicollis*	[293-369]
Isocresol [2-Methoxy-5-methylphenol]	C8H10O2/1195-09-1	Chlaeniini	*Chlaenius cordicollis*	[293]
Ipsdienol [2-Methyl-6-methylne-2,7-octadien-4-ol]	C10H160/54809-53-9	Pterostichini	*Pterostichus diligendus*	[358]
		Pterostichini	*Pterostichus melanarius*	[358]
1S5S-cis-verbenol [(1S,5S)-4,6,6-Trimethylbicyclo[3.1.1]hept-3-en-2-ol]	C10H160/ unvailable	Pterostichini	*Pterostichus diligendus*	[358]
		Pterostichini	*Pterostichus melanarius*	[358]
Formaldehyde	CH20/50-00-0	Platynini	*Anchomenus dorsalis*	[319]
		Carabini	*Autocarabus cancellatus*	[319]
		Carabini	*Carabus problematicus*	[329]
		Pterostichini	*Poecilus cupreus*	[319]
		Pterostichini	*Pterostichus melanarius*	[319]
Eucalyptol [1,3,3-Trimethyl-2-oxabicyclo[2.2.2]octane]	C10H180/470-82-6	Clivinini	*Ardistomis schaumii*	[367]
p-cymene [1-Methyl-4-(1-methylethyl)-benzene]	C10H14/99-87-6	Clivinini	*Semiardistomis puncticollis*	[367]
		Clivinini	*Ardistomis sp*	[367]
me-3me-2-butenyl-sulfide [Methyl(3-methylbut-2-enyl)-sulfide]	C6H12S/5897-45-0	Ceroglossini	*Ceroglossus buqueti*	[59]
		Ceroglossini	*Ceroglossus magellanicus*	[59]
2me5me-phenol [2,5-Dimethylphenol]	C8H10O/95-87-4	Chlaeniini	*Chlaenius cordicollis*	[293]
		Chlaeniini	*Chlaenius velutinus*	[108]
		Panagaeini	*Craspedophorus sp*	[325]
Heptacedadiene	C17H32/unvailable	Crepidogastrini	*Crepidogaster ambreana*	[104]
		Crepidogastrini	*Crepidogaster atrata*	[104]
3-Ethylphenol	C8H10O/620-17-7	Chlaeniini	*Chlaenius cordicollis*	[293]
		Panagaeini	*Craspedophorus australasiae*	[332]
		Panagaeini	*Craspedophorus mastersi*	[332]
		Panagaeini	*Craspedophorus rockhamptonensis*	[332]
2me3me-phenol [2,3-Dimethylphenol]	C8H10O/526-75-0	Chlaeniini	*Chlaenius cordicollis*	[293]
		Chlaeniini	*Chlaenius velutinus*	[109]
3m4me-phenol [3,4-Dimethylphenol]	C8H10O/95-65-8	Chlaeniini	*Chlaenius cordicollis*	[293]
2me-3-buten-2-ol [2_Methyl-3-buten-2-ol]	C5H10O/115-18-4	Pterostichini	*Pterostichus diligendus*	[358]
		Pterostichini	*Pterostichus melanarius*	[358]
5-2Kt [Pentan-2-one]	C5H10O/107-87-9	Clivinini	*Dyschirius wilsoni*	[357]
7-2Kt [Heptan-2-one]	C7H14O/110-43-0	Clivinini	*Dyschirius wilsoni*	[357]

15-2Kt [Pentadecan-2-one]	C15H30O/2345-28-0	Odacanthini	*Colliuris pensylvanica*	[320]
		Loxandrini	*Loxandrus longisformis*	[332]
		Loxandrini	*Loxandrus sp*	[320]
			Notagonum submetallicum	[332]
		Loxandrini	*Oxycrepis sp*	[320]
		Cyclosomini	*Sarothrocrepis civica*	[332]
		Cyclosomini	*Sarothrocrepis corticallis*	[332]
Decyl formate	C11H22O2/5451-52-5	Dryptini	*Drypta australis*	[332]
		Dryptini	*Drypta japonica*	[353]
		Galeritini	*Galerita japonica*	[353]
		Galeritini	*Galerita lecontei*	[334]
		Zuphiini	*Planetes puncticeps*	[353]
Me-2-hydroxy-6me-benzoate [Methyl 2-hydroxy-6-methylbenzoate]	C9H10O3/33528-09-5	Clivinini	*Dyschirius wilsoni*	[357]
Iridodial [2-Methyl-5-(1-oxopropan-2-yl)-cyclopentanecarbaldehyde]	C10H16O2/550-45-8	Clivinini	*Dyschirius wilsoni*	[357]
2me-benzene-1,4-diol [2_methylbenzene-1,4-diol]	C7H8O2/unvailable	Ozaenini	*Goniotropis nicaraguensis*	[337]
Hydrogen peroxide	H2O2/7722-84-1	Ozaenini	*Goniotropis nicaraguensis*	[337]
		Metriini	*Metrius contractus*	[337]
Hydrquinone [1,4-Dihydroxybenzene]	C6H6O2/123-31-9	Ozaenini	*Goniotropis nicaraguensis*	[337]
		Metriini	*Metrius contractus*	[337]
Nonyl formate	C10H20O2/5451-92-3		*Helluo costatus*	[325]
S-limonene [(S)-1-Methyl-4-(1-methylethenyl)-cyclohexene]	C10H16/5989-54-8	Clivinini	*Ardistomis schaumii*	[367]
		Clivinini	*Semiardistomis puncticollis*	[367]
R-limonene/(R)-1-Methyl-4-1-(1-methylethenyl)-cyclohexene]	C10H16/5989-27-5	Clivinini	*Ardistomis schaumii*	[367]
		Clivinini	*Ardistomis sp*	[367]
		Clivinini	*Semiardistomis puncticollis*	[367]
Sabinene[1-Isopropyl-4-methylenebicyclo[3.1.0]hexane]	C10H16/3387-41-5	Clivinini	*Semiardistomis puncticollis*	[367]
Beta-phellandrene [4-Isopropyl-1-methylene-2-cyclohexene]	C10H16/555-10-2	Clivinini	*Semiardistomis puncticollis*	[367]
Beta-pinene [6,6-Dimethyl-2-methylenebicyclo[3.1.1]heptane]	C10H16/127-91-3	Clivinini	*Semiardistomis puncticollis*	[367]

Appendix A

Family	compound/CAS	Formula/CAS	References
ALLECULINAE	toluquinone [2-Methyl-1,4-benzoquinone]	C7H6O2/ 553-97-9	[234-268]
	2-ethyl-1,4-benzoquinone [2-Ethyl-1,4-benzoquinone]	C8H8O2/ 4754-26-1	[268]
	delta1-15Hy [1-Pentadecene]	C15H30/ 13360-61-7	[234]
	delta1-21Hy [1-Heneicosene]	C21H42/ 27400-79-9	[234]
ANOBIIDAE	S-perillaldehyde [(S)-4-(Prop-1-en-2-yl)-cyclohex-1-enecarbaldehyde]	C10H14O/ 18031-40-8	[270]
	S-limonene [(S)-1-Methyl-4-(1-methylethenyl)-cyclohexene]	C10H16/ 5989-54-8	[270]
	E-beta-farnesene [(E)-7,11-Dimethyl-3-methylene-1,6,10-dodecatriene]	C15H24/ 18794-84-8	[270]
	beta-caryophyllene [1R-(1R,4E,9S)-4,11,11-Trimethyl-8-methylenebicyclo[7.2.0]undec-4-ene]	C15H24/ 87-44-5	[270]
	beta-thujaplicine [2-Hydroxy-4-isopropyl-2,4,6-cycloheptatrien-1-one]	C10H12O2/ 499-44-5	[271]
BORIDAE	beta-necrodol [((1R,3R)-2,2,3-Trimethyl-4-methylenecyclopentyl)-methanol]	C10H18O/unvailable	[188]
BOSTRICHIDAE	E-cinnamaldehyde [(E)-3-Phenyl-2-propenal]	C9H8O/ 14371-10-9	[272]
	beta-necrodol [((1R,3R)-2,2,3-Trimethyl-4-methylenecyclopentyl)-methanol]	C10H18O/unvailable	[188]
	S-heptan-2-ol [(S)-Heptan-2-ol]	C7H16O/ 6033-23-4	[274]
BUPRESTIDAE	buprestin A [beta-D-Glucopyranose 1,2,6-tris(pyrrole-2-carboxylate)]	C21H22N3O9/unvailable	[276]
	buprestin B [beta-D-Glucopyranose 6-(4-hydroxybenzoate) 1,2-bis(pyrrole-2-carboxylate)]	C23H22N2O10/unvailable	[276]
	buprestin D [Buprestin D]	C25H24N2O10/unvailable	[276]
	buprestin E [Buprestin E]	C26H26N2O11/	[277]
	buprestin F [Buprestin F]	C25H24N2O11/	[361]

	buprestin G [Buprestin G]	C24H24N2O11/	[362]
	buprestin H [Buprestin H]	C24H24N2O10/	[277]
CANTHARIDAE	cantharidin [2,3-Dimethyl-7-oxabicyclo[1,2,2]heptane-2,3-dicarboxylic anhydride]	C10H12O4/ 56-25-7	[184]
	senecionine [Senecionine]	C18H25NO5/ 130-01-8	[273]
	integerrimine [Integerrimine]	C18H25NO5/ 480-79-5	[266-267]
	retrorsine [Retrorsine]	C18H25NO6/ 480-54-6	[359]
	usaramine [Usaramine]	C18H25NO6/ 15503-87-4	[220]
	Y4Y6-Z8-10Acid [(Z)-Deca-8-en-4,6-diynoic acid]	C10H10O2/115605-26-0	[278]
	precoccinelline [Precoccinelline]	C13H23N/38211-56-2	[261]
	hippodamine [Hippodamine]	C13H23N/ unvailable	[184]
	propyleine [Propyleine]	C13H21N/unvailable	[264]
	2-sec-butyl-3-methoxypyrazine [2-sec-Butyl-3-methoxypyrazine]	C9H14N2O/24168-70-5	[275]
CERAMBYCIDAE	cis,trans-iridodial [(1S,2S,5R)-2-Methyl-5-((R)-1-oxopropan-2-yl)-cyclopentanecarbaldehyde]	C10H16O2/unvailable	[262]
	cis-rose oxide [(2R,4S)-Tetrahydro-4-methyl-2-(2-methylprop-1-enyl)-2H-pyran]	C10H18O/3033-23-6	[265]
	trans-rose oxide [(2R,4R)-Tetrahydro-4-methyl-2-(2-methylprop-1-enyl)-2H-pyran]	C10H18O/876-18-6	[278]
	salicylaldehyde [2-Hydroxybenzaldehyde]	C7H6O2/90-02-8	[278]
	cantharidin [2,3-Dimethyl-7-oxabicyclo[1,2,2]heptane-2,3-dicarboxylic anhydride]	C10H12O4/56-25-7	[184]
	trans,trans-iridodial [(1R,2S,5S)-2-Methyl-5-((R)-1-oxopropan-2-yl)-cyclopentanecarbaldehyde]	C10H16O2/unvailable	[264]
	2-hydroxy-6me-benzaldehyde [2-Hydroxy-6-methylbenzaldehyde]	C8H8O2/18362-36-2	[275]
	phoracanthol [5-Ethylcyclopent-1-enyl-methanol]	C8H14O/unvailable	[275]
	phoracanthal [5-Ethylcyclopent-1-ene-carbaldehyde]	C8H12O/ unvailable	[275]
	alpha-terpineol [2-(4-Methylcyclohex-3-enyl)-propan-2-ol]	C10H18O/ 98-55-5	[262]
	cis-nerolidol [(Z)-3,7,11-Trimethyl-1,6,10-dodecatrien-3-ol]	C15H26O/ 3790-78-1	[262]

	delta-cadinene [(1S,8aR)-4,7-Dimethyl-1-(propan-2-yl)-1,2,3,5,6,8a-hexahydronaphthalene]	C15H24/ 483-76-1	[262]
	alpha-terpinolene [1-Methyl-4-(1-methylethylidene)-cyclohexene]	C10H16/ 586-62-9	[262]
	cedrol [Cedrol]	C15H26O/ 77-53-2	[262]
	toluene [Toluene]	C7H8/ 108-88-3	[265]
	2-phenylacetic acid [2-Phenylacetic acid]	C8H8O2/ 103-82-2	[265]
CHRYSOMELIDAE	alpha-caryophyllene [(E,E,E)-2,6,6,9-Tetramethyl-1,4,8-cycloundecatriene]	C15H24/ 6753-98-6	[260]
	caprylic acid [Octanoic acid]	C8H16O2/ 124-07-2	[364]
	palmitic acid [Hexadecanoic acid]	C16H32O2/ 57-10-3	[363]
	ethyl palmitate [Ethyl hexadecanoate]	C18H36O2/ 628-97-7	[259]
	ethyl linoleate [Ethyl (Z,Z)-9,12-octadecadienoate]	C20H36O2/ 544-35-4	[263]
	methyl palmitate [Methyl hexadecanoate]	C17H34O2/ 112-39-0	[365]
	E-phytol [(E)-3,7,11,15-Tetramethyl-2-hexadecen-1-ol]	C20H40O/ 150-86-7	[188]
	methyl linolenate [Methyl (Z,Z,Z)-9,12,15-octadecatrienoate]	C19H32O2/ 301-00-8	[258]
	methyl stearate [Methyl octadecanoate]	C19H38O2/ 112-61-8	[256]
	sabinene [1-Isopropyl-4-methylenebicyclo[3.1.0]hexane]	C10H16/ 3387-41-5	[269]
	limonene [1-Methyl-4-(1-methylethenyl)-cyclohexene]	C10H16/ 138-86-3	[184]
	beta-phellandrene [4-Isopropyl-1-methylene-2-cyclohexene]	C10H16/ 555-10-2	[363]
	2-aminoethanol [2-Aminoethanol]	C2H7NO/ 141-43-5	[255]
	diacetyl putrescine [N-Acetyl-N-(4-aminobutyl)-acetamide]	C8H16N2O2/unvailable	[254]
	diphenylamine [Diphenylamine]	C12H11N/ 122-39-4	[254]
	l-tryptophan [(S)-2-Amino-3-(1H-indol-3-yl)-propanoic acid]	C11H12N2O2/ 73-22-3	[261]
	l-leucine [(S)-2-Amino-4-methylpentanoic acid]	C6H13NO2/ 61-90-5	[252]
	20-hydroxyecdysone 22-acetate [20-Hydroxyecdysone 22-acetate]	C29H46O8/unvailable	[251]
	sophoroside 4 [Sophoroside 4]	C41H66O16/ 40381-63-1	[248]

	sophoroside 5 [Sophoroside 5]	C41H66O16/ 140381-64-2	[248]
	sophoroside 7 [Sophoroside 7]	C41H70O16/ 140381-61-9	[247]
	sophoroside 8 [Sophoroside 8]	C41H70O16/ 140381-62-0	[248-292]
	beta-necrodol [((1R,3R)-2,2,3-Trimethyl-4-methylenecyclopentyl)-methanol]	C10H18O/unvailable	[332]
	13-2Kt [Tridecan-2-one]	C13H26O/ 593-08-8	[246]
	E2Z6-9Ald [(E,Z)-2,6-Nonadienal]	C9H14O/ 557-48-2	[244]
	E3E5-8-2Kt [(E,E)-3,5-Octadien-2-one]	C8H12O/ 30086-02-3	[258]
	E3Z7-10-2Kt [(E,Z)-3,7-Decadien-2-one]	C10H16O/unvailable	[258]
	chrysophanol [1,8-Dihydroxy-3-methyl-9,10-anthraquinone]	C15H10O4/ 481-74-3	[256]
	chrysazin [1,8-Dihydroxyanthraquinone]	C14H8O4/ 117-10-2	[256]
	Z11-20Ac [(Z)-11-Eicosenyl acetate]	C22H42O2/ 70792-86-8	[253-269]
	18Ac [Octadecyl acetate]	C20H40O2/ 822-23-1	[253]
	2-amino-Z3,5-hexadienoic acid [(Z)-2-Aminohexa-3,5-dienoic acid]	C6H9NO2/unvailable	[226]
	gamma-glutamylethanolamine [(S)-2-Amino-5-(2-hydroxyethylamino)-5-oxopentanoic acid]	C7H14N2O4/unvailable	[226]
	senecionine oxide [Senecionine N-oxide]	C19H27NO6/ 13268-67-2	[255]
	doronenine oxide [Doronenine N-oxide]	C18H25NO6/unvailable	[255]
	bulgarsenine oxide [Bulgarsenine N-oxide]	C18H27NO6/ unvailable	[255]
	seneciphylline oxide [Seneciphylline N-oxide]	C18H23NO6/ 38710-26-8	[255]
	3S8S-chrysomelidial [(3S,8S)-2-Methyl-5-(1-formylethyl)-1-cyclopentene-1-carbaldehyde]	C10H14O2/ 63808-11-7	[253]
	salicylaldehyde [2-Hydroxybenzaldehyde]	C7H6O2/ 90-02-8	[261]
	beta-ionone [(E)-4-(2,6,6-Trimethylcyclohex-1-enyl)-3-buten-2-one]	C13H20O/ 79-77-6	[252]
	epiplagiolactone [(4R)-4,7-Dimethyl-4a,5-dihydrocyclopenta[c]pyran-3(4H)-one]	C10H12O2/ 66512-83-2	[253]
	retronecine [Retronecine	C8H13NO2/ 480-85-3	[248]

	O7-2-hydroxyisovaleryl-retronecine [O7-(2-Hydroxyisovaleryl)-retronecine]	C13H21NO4/unvailable	[248]
	O9-2-hydroxyisovaleryl-retronecine [O9-(2-Hydroxyisovaleryl)-retronecine]	C13H21NO4/unvailable	[248]
	intermedine [Intermedine]	C15H25NO5/unvailable	[248]
	lycopsamine [Lycopsamine]	C15H25NO5/ 10285-07-1	[248]
	O2-acetylintermedine [O2-Acetylintermedine]	C17H27NO6/unvailable	[248]
	chrysarobin [1,8-Dihydroxy-3-methylanthrone]	C15H12O3/ 491-59-8	[247]
	dithranol [1,8,9-Trihydroxyanthracene]	C14H10O3/ 480-22-8	[256]
CICINDELIDAE	benzaldehyde [Benzaldehyde]	C7H6O/ 100-52-7	[249]
	ethyl alcohol [Ethanol]	C2H6O/ 64-17-5	[250]
	mandelonitrile [2-Hydroxy-2-phenylacetonitrile]	C8H7NO/ 532-28-5	[292]
	benzoic acid [Benzoic acid]	C7H6O2/ 65-85-0	[244]
	2-phenylacetic acid [2-Phenylacetic acid]	C8H8O2/ 103-82-2	[244]
CLERIDAE	cantharidin [2,3-Dimethyl-7-oxabicyclo[1,2,2]heptane-2,3-dicarboxylic anhydride]	C10H12O4/ 56-25-7	[180]
	R-palasonin [(R)-Palasonin]	C9H8O4	
	S-palasonin [(S)-Palasonin]	C9H8O4	
COCCINELLIDAE	adaline [Adaline]	C13H23NO	[243]
	adalinine [Adalinine]	C13H23NO2	[235]
	S-3-hydroxypiperidin-2Kt [(S)-3-Hydroxypiperidin-2-one]	C5H9NO2	[223]
	harmonine [Harmonine]	C18H40N2	[242]
	2-dehydrococcinelline [2-Dehydrococcinelline]	C13H22NO	[216]
	hippodamine [Hippodamine]	C13H23N	[369]
	calvine [Calvine]	C13H23NO2	[244]
	2-epicalvine [2-Epicalvine]	C13H23NO2	[371-372]
	propyleine [Propyleine]	C13H21N	[238]

	coccinelline [Coccinelline]	C13H23NO	[239]
	precoccinelline [Precoccinelline]	C13H23N/ 38211-56-2	[240]
	chilocorine C [Chilocorine C]	C27H38N2O2	[236]
	chilocorine B [Chilocorine B]	C26H32N2O	[118]
	chilocorine A [Chilocorine A]	C26H32N2O	[237]
	chilocorine D [Chilocorine D]	C27H34N2O2	[232]
	1-2S6S-6me-piperidin-2-yl-3-2Kt [1-((2S,6S)-6-Methylpiperidin-2-yl)-propan-2-one]	C9H17NO	[233]
	1me-9-azabicyclo-9-3Kt [1-Methyl-9-azabicyclo[3.3.1]nonan-3-one]	C9H15NO	[231]
	E,E-2,6-diacetoxy-1,4-germacradiene [(E,E)-2,6-Diacetoxygermacra-1(10),4-diene]	C19H30O4	[220]
	catalipyrones H [Catalipyrones H]	C23H30O5	[233]
	catalipyrones I [Catalipyrones I]	C23H28O6	[227]
	catalipyrones J [Catalipyrones J]	C25H32O7	[233]
	5-12-aminotridecyl-pyrrolidine [(5a,7a,b)-Hexahydro-a-methylpyrrolo[2,1-b]oxazole-5-dodecaneamine]	C19H32N2O	[229]
	1-2-hydroxyethyl-2-10-aminoundecyl-pyrrolidine [1-(2-Hydroxyethyl)-2-(10-aminoundecyl)-pyrrolidine]	C17H36N2O	[372]
	5-10-aminoundecyl-pyrrolidine [(5a,7a,b)-Hexahydro-a-methylpyrrolo[2,1-b]oxazole-5-decaneamine]	C17H28N2O	[226]
	1-2-hydroxyethyl-2-12-aminotridecyl-pyrrolidine [1-(2-Hydroxyethyl)-2-(12-aminotridecyl)-pyrrolidine]	C19H40N2O	[230]
	euphococcinine [9-Aza-1-methyl-bicyclo[3.3.1]nonan-3-one]	C9H15NO/ 45977-26-2	[224]
	1-6me-2-piperidyl-3-2Kt [1-(6-Methyl-2-piperidyl)-propan-2-one]	C9H17NO7 83285-66-9	[225]
	1-6me-2,3,4,5-tetrahydropyridin-2-yl-3-2Kt [1-(6-Methyl-2,3,4,5-tetrahydropyridin-2-yl)-propan-2-one]	C9H15NO	[219]
	choline [Choline]	C5H14NO+/ 62-49-7	[221]
	signatipennine [Signatipennine]	C20H44N2O2	[222]
	2S12R-2-12-aminotridecyl-pyrrolidine [(2S,12R)-2-(12-Aminotridecyl)-pyrrolidine]	C17H36N2	[220]

	2S12R-1-2-hydroxyethyl-2-12-aminotridecyl-pyrrolidine [(2S,12R)-1-(2-Hydroxyethyl)-2-(12-aminotridecyl)-pyrrolidine]	C19H40N2O	[218]
	2me-6-propylpiperidine [2-Methyl-6-propylpiperidin	C9H19N/ 68170-79-6	[216]
	2-phenylethanamine [2-Phenylethylamine]	C8H11N/ 64-04-0	[217]
	2-12-aminotridecyl-pyrrolidine [2-(12-Aminotridecyl)-pyrrolidine]	C17H36N2	[231]
	epilachnene [(Z)-11-Propyl-12-azacyclotetradec-5-en-14-olide]	C17H29NO2/ 147363-82-4	[230]
	epilachnadiene [(Z,Z)-11-Propyl-12-azacyclotetradec-5,8-dien-14-olide]	C17H27NO2/ 147363-83-5	[230]
	norepilachnene [(Z)-11-Ethyl-12-azacyclotetradecen-14-olide]	C16H27NO2/ 147363-86-8	[230]
	9-propyl-10-azacyclododecan-12-olide [9-Propyl-10-azacyclododecan-12-olide]	C14H27NO2	[230]
	homoepilachnene [(Z)-12-Propyl-13-azacyclopentadec-5-en-15-olide]	C18H31NO2/ 147363-84-6	[230]
	2-isobutyl-3-methoxypyrazine [2-Isobutyl-3-methoxypyrazine]	C9H14N2O/ 24683-00-9	[220]
	exochomine [Exochomine]	C26H36N2O	[226]
	isopsylloborine A [Isopsylloborine A]	C26H40N2	[224]
	DEET [N,N-Diethyl-3-methylbenzamide]	C12H17NO/ 134-62-3	[225]
	hippocasine N-oxide [Hippocasine N-oxide]	C13H22NO	[117]
	convergine [Convergine]	C13H24NO	[117]
	hippocasine [Hippocasine]	C13H21N/ 75556-13-7	[117]
	hyperaspine [Hyperaspine]	C19H30N2O3	[221]
	carminic acid [Carminic acid]	C22H20O13/ 1260-17-9	[222]
	2-sec-butyl-3-methoxypyrazine [2-sec-Butyl-3-methoxypyrazine]	C9H14N2O/ 24168-70-5	[220]
	myrrhine [Myrrhine]	C13H23N	[245]
	psylloborine A [Psylloborine A]	C26H40N2	[218]
	2-isopropyl-3-methoxypyrazine [2-Isopropyl-3-methoxypyrazine]	C8H12N2O/ 25773-40-4	[220]
	chrysarobin [1,8-Dihydroxy-3-methylanthrone]	C15H12O3/ 491-59-8	[212]

CURCULIONIDAE	Z3-6Ac [(Z)-3-Hexenyl acetate]	C8H14O2/ 3681-71-8	[215]
	beta-necrodol [((1R,3R)-2,2,3-Trimethyl-4-methylenecyclopentyl)-methanol]	C10H18O	[188]
	alpha-terpineol [2-(4-Methylcyclohex-3-enyl)-propan-2-ol]	C10H18O/ 98-55-5	[214]
	delta2-6Ald [2-Hexenal]	C6H10O/ 505-57-7	[213]
	6Ald [Hexanal]	C6H12O/ 66-25-1	[213]
	2-cyclohexen-1-one [2-Cyclohexen-1-one]	C6H8O/ 930-68-7	[213]
	alpha-caryophyllene [(E,E,E)-2,6,6,9-Tetramethyl-1,4,8-cycloundecatriene]	C15H24/ 6753-98-6	[213]
	eugenol [4-Allyl-2-methoxyphenol]	C10H12O2/ 97-53-0	[213]
	phenylmethanol [Phenylmethanol]	C7H8O/ 100-51-6	[213]
	eucalyptol [1,3,3-Trimethyl-2-oxabicyclo[2.2.2.]octane]	C10H18O/ 470-82-6	[213]
DERODONTIDAE	chrysarobin [1,8-Dihydroxy-3-methylanthrone]	C15H12O3/ 491-59-8	[217]
DYTISCIDAE	4-hydroxybenzoic acid [4-Hydroxybenzoic acid]	C7H6O3/ 99-96-7	[189]
	hydroquinone [1,4-Dihydroxybenzene]	C6H6O2/ 123-31-9	[207]
	4-hydroxybenzaldehyde [4-Hydroxybenzaldehyde]	C7H6O2/ 123-08-0	[211]
	me-4-hydroxybenzoate [Methyl 4-hydroxybenzoate]	C8H8O3/ 99-76-3	[210-176]
	cortexone [21-Hydroxypregn-4-en-3,20-dione]	C21H30O3/ 64-85-7	[108]
	20a-progerol [20a-Hydroxy-4-pregnen-3-one]	C21H32O2/ 145-14-2	[204]
	cybisterone [20a-Hydroxy-4,6-pregnadien-3-one]	C21H30O2/ 15271-87-1	[206]
	20b-4-pregnenol-acetoxy-3Kt [20b-4-Pregnenol-acetoxy-3-one]	C22H34O	[176]
	2-phenylacetic acid [2-Phenylacetic acid]	C8H8O2/ 103-82-2	[203]
	benzoic acid [Benzoic acid]	C7H6O2/ 65-85-0	[176]
	me-3,4-dihydroxybenzoate [Methyl 3,4-dihydroxybenzoate]	C8H8O4/ 2150-43-8	[176]
	me-2-2,5-dihydroxyphenyl-acetate [Methyl 2-(2,5-dihydroxyphenyl)-acetate]	C9H10O4	[201]
	marginalin [(Z)-5-Hydroxy-3-(4-hydroxybenzylidene)-benzofuran-2(3H)-one]	C15H10O4	[176-200]

	phenylpyruvic acid [Phenylpyruvic acid]	C9H8O3/ 156-06-9	[199-176]
	3a11a-dihydroxy-5b-pregnan-20Kt [3a,11a-Dihydroxy-5b-pregnan-20-one]	C21H34O3/ 600-52-2	[198]
	hydroxyphenylacetic acid [p-Hydroxyphenylacetic acid]	C8H8O3/ 156-38-7	[189]
	3-indoleacetic acid [2-(1H-Indol-3-yl)-acetic acid]	C10H9NO2/ 87-51-4	[189]
	3-phenylpropanoic acid [3-Phenylpropanoic acid]	C9H10O2/ 501-52-0	[178]
	tiglic acid [(E)-2-Methyl-2-butenoic acid]	C5H8O2/ 80-59-1	[178]
	me 8-hydroxyquinoline-2-carboxylate [Methyl-8-hydroxyquinoline-2-carboxylate]	C11H9NO3	[202]
	testosterone [17b-Hydroxy-4-androsten-3-one]	C19H28O2/ 58-22-0	[201]
	me-2-3,4-dihydroxyphenyl-acetate [Methyl 2-(3,4-dihydroxyphenyl)-acetate]	C9H10O4	[189]
	3-hydroxy-8Acid [3-Hydroxyoctanoic acid]	C8H16O3/ 14292-27-4	[189]
	3-hydroxy-9Acid [3-Hydroxynonanoic acid]	C9H18O3/ 40165-87-5	[189]
	myrmicacin [(R)-3-Hydroxydecanoic acid]	C10H20O3/ 19525-80-5	[189]
	ethyl benzoate [Ethyl benzoate]	C9H10O2/ 93-89-0	[199]
	platambin [2-Isopropyl-4a-methyl-8-methylenedecahydro-1,5-naphthalenediol]	C15H26O2/58556-80-2	[200]
	mirasorvone [Mirasorvone]	C21H28O3	[198]
ELATERIDAE	1H-indole [2,3-Benzopyrrole]	C8H7N/ 120-72-9	[197]
	1me2me-disulfide [1,2-Dimethyldisulfide]	C2H6S2/ 624-92-0	[197]
	1me3me-trisulfide [1,3-Dimethyltrisulfide]	C2H6S3/ 3658-80-8	[197]
	1me4me-tetrasulfide [1,4-Dimethyltetrasulfide]	C2H6S4/ 5756-24-1	[197]
ENDOMYCHIDAE	2-sec-butyl-3-methoxypyrazine [2-sec-Butyl-3-methoxypyrazine]	C9H14N2O/ 24168-70-5	[220]
	stenotarsol [7-(Hydroxymethyl)-5,7-dimethyl-7,8-dihydronaphtho[1,2-c]furan-3,9(1H,6H)-dione]	C15H16O4	[196]
EROTYLIDAE	benzaldehyde [Benzaldehyde]	C7H6O/ 100-52-7	[195]
	phenylmethanol [Phenylmethanol]	C7H8O/ 100-51-6	[194]

	benzothiazole [Benzothiazole]	C7H5NS/ 95-16-9	[194]
	p-anisaldehyde [4-Methoxybenzaldehyde]	C8H8O2/ 123-11-5	[194]
	benzophenone [Benzophenone]	C13H10O/ 119-61-9	[194]
	23Hy [Tricosane]	C23H48/ 638-67-5	[194]
	25Hy [Pentacosane]	C25H52/ 629-99-2	[194]
GYRINIDAE	isogyrinidal [(E,E,Z)-3,7-Dimethyl-8,11-dioxo-2,6,9-dodecatrienal]	C14H18O3	[191]
	gyrinidal [(E,E,E)-3,7-Dimethyl-8,11-dioxo-2,6,9-dodecatrienal]	C14H18O3/ 6518-11-3	[192]
	gyrinidone [Gyrinidone]	C14H20O3/ 9013-24-6	[193]
	gyrinidione [Gyrinidione]	C14H20O3	[191]
	sovaleraldehyde [3-Methylbutanal]	C5H10O/ 590-86-3	[373]
	3me-4OH [3-Methylbutan-1-ol]	C5H12O/ 123-51-3	[373]
	isovaleraldehyde [3-Methylbutanal]	C5H10O/ 590-86-3	[373]
	2-phenylacetaldehyde [2-Phenylacetaldehyde]	C8H8O/ 122-78-1	[373]
HALIPLIDAE	2-phenylacetic acid [2-Phenylacetic acid]	C8H8O2/ 103-82-2	[189]
HELODIDAE	beta-necrodol [((1R,3R)-2,2,3-Trimethyl-4-methylenecyclopentyl)-methanol]	C10H18O	[188]
HYDROPHILIDAE	beta-necrodol [((1R,3R)-2,2,3-Trimethyl-4-methylenecyclopentyl)-methanol]	C10H18O	[188]
LAMPYRIDAE	1-oxo-12-O-acetyl-3b-dihydroxy-5b11a-bufaline [1-oxo-12-O-Acetyl-3b-dihydroxy-5b,11a-bufaline]	C26H34O8	[187]
	1-oxo-11-O-acetyl-3b-dihydroxy-5b12b-bufaline [1-oxo-11-O-Acetyl-3b-dihydroxy-5b,12b-bufaline]	C26H34O7	[187]
	1-oxo-12-dihydroxy-5b11a-bufaline [1-oxo-12-Dihydroxy-5b,11a-bufaline]	C24H32O7	[187]
	1-oxo-11-dihydroxy-5b12b-bufaline [1-oxo-11-Dihydroxy-5b,12b-bufaline]	C24H32O6	[187]
	N-methylquinolinium 2-carboxylate [N-Methylquinolinium 2-carboxylate]	C11H9NO2	[186]
LYCIDAE	lycidic acid [(E,E)-Octadeca-5,7-dien-9-ynoic acid]	C18H28O2	[185]
	2-isopropyl-3-methoxypyrazine [2-Isopropyl-3-methoxypyrazine]	C8H12N2O/ 25773-40-4	[185]

	2-sec-butyl-3-methoxypyrazine [2-sec-Butyl-3-methoxypyrazine]	C9H14N2O/ 24168-70-5	[220]
MELOIDAE	l - cantharidin [2,3-Dimethyl-7-oxabicyclo[1,2,2]heptane-2,3-dicarboxylic anhydride]	C10H12O4/ 56-25-7	[184]
	palasonin [Palasonin]	C9H10O4	[183-179]
	palasoninimide [Palasoninimide]	C9H11NO3	[179]
	cantharidinimide [Cantharidinimide]	C10H13NO3	[179]
	2-sec-butyl-3-methoxypyrazine [2-sec-Butyl-3-methoxypyrazine]	C9H14N2O/ 24168-70-5	[220]
	2-isobutyl-3-methoxypyrazine [2-Isobutyl-3-methoxypyrazine]	C9H14N2O/ 24683-00-9	[185]
	2-isopropyl-3-methoxypyrazine [2-Isopropyl-3-methoxypyrazine]	C8H12N2O/ 25773-40-4	[185]
MELYRIDAE	cantharidin [2,3-Dimethyl-7-oxabicyclo[1,2,2]heptane-2,3-dicarboxylic anhydride]	C10H12O4/ 56-25-7	[184]
NOTERIDAE	2-phenylacetic acid [2-Phenylacetic acid]	C8H8O2/ 103-82-2	[176]
	hydroxyphenylacetic acid [p-Hydroxyphenylacetic acid]	C8H8O3/ 156-38-7	[176]
	3-indoleacetic acid [2-(1H-Indol-3-yl)-acetic acid]	C10H9NO2/ 87-51-4	[175]
	phenylpyruvic acid [Phenylpyruvic acid]	C9H8O3/ 156-06-9	[176]
	4-hydroxybenzoic acid [4-Hydroxybenzoic acid]	C7H6O3/99-96-7	[176]
	benzoic acid [Benzoic acid]	C7H6O2/ 65-85-0	[176]
OEDEMERIDAE	cantharidin [2,3-Dimethyl-7-oxabicyclo[1,2,2]heptane-2,3-dicarboxylic anhydride	C10H12O4/ 56-25-7	[174]
			[173]
			[169-170]
			[172]
	2-sec-butyl-3-methoxypyrazine [2-sec-Butyl-3-methoxypyrazine]	C9H14N2O/ 24168-70-5	[171]
PYROCHROIDAE	cantharidin [2,3-Dimethyl-7-oxabicyclo[1,2,2]heptane-2,3-dicarboxylic anhydride]	C10H12O4/ 56-25-7	[167]
RHAGOPHTHALMINAE	1,4-naphthoquinone [1,4-Naphthoquinone]	C10H6O2/ 130-15-4	[166]
SCARABEIDAE	beta-necrodol [((1R,3R)-2,2,3-Trimethyl-4-methylenecyclopentyl)-methanol]	C10H18O	[188]

	phenol [Phenol]	C6H6O/ 108-95-2	[164]
	guaiacol [2-Methoxyphenol]	C7H8O2/ 90-05-1	[165]
	m-cresol [3-Methylphenol]	C7H8O/ 108-39-4	[165]
	p-creosol [2-Methoxy-4-methylphenol]	C8H10O2/ 93-51-6	[163]
	1H-indole [2,3-Benzopyrrole]	C8H7N/ 120-72-9	[165]
	1,4-benzoquinone [1,4-Benzoquinone]	C6H4O2/ 106-51-4	[163]
	methyl salicylate [Methyl salicylate]	C8H8O3/ 119-36-8	[374]
SCOLYTIDAE	alpha-pinene [2,6,6-Trimethylbicyclo[3.1.1]hept-2-ene]	C10H16/ 80-56-8	[160]
	beta-necrodol [((1R,3R)-2,2,3-Trimethyl-4-methylenecyclopentyl)-methanol]	C10H18O	[188]
	delta1-octen-3-ol [1-Octen-3-ol]	C8H16O/ 3391-86-4	[160]
	seudenone [3-Methyl-2-cyclohexen-1-one]	C7H10O/ 1193-18-6	[160]
	acetophenone [1-Phenylethanone]	C8H8O/ 98-86-2	[161]
	trans-verbenol [trans-4,6,6-Trimethylbicyclo[3.1.1]hept-3-en-2-ol]	C10H16O/ 1820-09-3	[160]
	cis-verbenol [cis-4,6,6-Trimethylbicyclo[3.1.1]hept-3-en-2-ol]	C10H16O/ 18881-04-4	[160]
	beta-caryophyllene [1R-(1R,4E,9S)-4,11,11-Trimethyl-8-methylenebicyclo[7.2.0]undec-4-ene]	C15H24/ 87-44-5	[159]
	trans-sabinene hydrate [(1R,2R,5S)-5-Isopropyl-2-methylbicyclo[3.1.0]hexan-2-ol]	C10H18O/ 17699-16-0	[158]
SILPHIDAE	caprylic acid [Octanoic acid]	C8H16O2/ 124-07-2	[188]
	caprinic acid [Decanoic acid]	C10H20O2/ 334-48-5	[188]
	Z3-10Acid [(Z)-3-Decenoic acid]	C10H18O2/ 2430-93-5	[155]
	Z4-10Acid [(Z)-4-Decenoic acid]	C10H18O2/ 505-90-8	[331]
	palmitic acid [Hexadecanoic acid]	C16H32O2/ 57-10-3	[188]
	stearic acid [Octadecanoic acid]	C18H36O2/ 57-11-4	[188]
	lavandulol [2-Isopropenyl-5-methyl-4-hexen-1-ol]	C10H18O/ 58461-27-1	[188]
	alpha-necrodol [((1R,4S)-3,4,5,5-Tetramethylcyclopentenyl)-methanol]	C10H18O	[188]

	beta-necrodol [((1R,3R)-2,2,3-Trimethyl-4-methylenecyclopentyl)-methanol]	C10H18O	[188]
	15b-hydroxyprogesterone [15b-Hydroxypregn-4-en-3,20-dione]	C21H30O3/ 600-72-6	[155]
	5b-pregnan-15b-ol-3,20Kt [5b-Pregnan-15b-ol-3,20-dione]	C21H32O3	[155]
	5b-pregnan-3a7b15b-triol-20Kt [5b-Pregnan-3a,7b,15b-triol-20-one]	C21H34O4	[155]
	5b-pregnan-16a-ol-3,20Kt [5b-Pregnan-16a-ol-3,20-dione]	C21H32O3	[155]
	ammonia [Ammonia]	H3N/ 7664-41-7	[331]
	3a7b-dihydroxy-14b-pregn-4-en-15,20Kt [3a,7b-Dihydroxy-14b-pregn-4-en-15,20-dione]	C21H30O4	[375]
	3a7b20xi-trihydroxy-14b-4-pregnen-15Kt [3a,7b,20xi-Trihydroxy-14b-pregn-4-en-15-one]	C21H32O4	[375]
STAPHYLINIDAE	11Hy [Undecane]	C11H24/1120-21-4	[152]
	delta1-11Hy [1-Undecene]	C11H22/821-95-4	[156]
	12Ald [Dodecanal]	C12H24O/112-54-9	[154]
	toluquinone [2-Methyl-1,4-benzoquinone]	C7H6O2/553-97-9	[153]
	2-methoxy-3me-1,4-benzoquinone [2-Methoxy-3-methyl-1,4-benzoquinone]	C8H8O3/2207-57-0	[156]
	Z4-13Hy [(Z)-4-Tridecene]	C13H26/41446-54-2	[151]
	Z5-14Ald [(Z)-5-Tetradecenal]	C14H26O	[152]
	caprylic acid [Octanoic acid]	C8H16O2/124-07-2	[150]
	19Hy [Nonadecane]	C19H40/629-92-5	[135]
	benzonitrile [Benzonitrile]	C7H5N/100-47-0	[154]
	E2-6Ald [(E)-2-Hexenal]	C6H10O/6728-26-3	[148]
	toluhydroquinone [2-Methyl-1,4-benzenediol]	C7H8O2/95-71-6	[149]
	hydroquinone [1,4-Dihydroxybenzene]	C6H6O2/123-31-9	[147]
	ethyl octadecenoate [Ethyl octadecenoate]	C20H38O2/94874-12-1	[146]
	ethyl octadecadienoate [Ethyl octadecadienoate]	C20H36O2	[154]
	10Ald [Decanal]	C10H20O/112-31-2	[153]

	13Hy [Tridecane]	C13H28/629-50-5	[145]
	delta1-17Hy [1-Heptadecene]	C17H34/6765-39-5	[153]
	1,4-benzoquinone [1,4-Benzoquinone]	C6H4O2/106-51-4	[153]
	2-methoxy-3me-hydroquinone [2-Methoxy-3-methylhydroquinone]	C8H10O3	[143]
	caprinic acid [Decanoic acid]	C10H20O2/ 334-48-5	[358]
	octyl octanoate [Octyl octanoate]	C16H32O2/2306-88-9	[188]
	delta1-13Hy [1-Tridecene]	C13H26/ 2437-56-1	[142]
	gamma-dodecalactone [5-Octyl-dihydrofuran-2(3H)-one]	C12H22O2/ 2305-05-7	[143]
	gamma-tetradecalactone [5-Decyl-dihydrofuran-2(3H)-one]	C14H26O2	[139]
	neral [(Z)-3,7-Dimethyl-2,6-octadienal]	C10H16O/ 106-26-3	[138]
	geranial [(E)-3,7-Dimethyl-2,6-octadienal]	C10H16O/ 141-27-5	[143]
	senecioic acid [3-Methyl-2-butenoic acid]	C5H8O2/ 541-47-9	[143]
	citral [(Z,E)-3,7-Dimethyl-2,6-octadienal]	C10H16O/ 5392-40-5	[133]
	10Ac [Decyl acetate]	C12H24O2/ 112-17-4	[136]
	11Ac [Undecyl acetate]	C13H26O2/ 1731-81-3	[137]
	12Ac [Dodecyl acetate]	C14H28O2/ 112-66-3	[143]
	actinidine [6,7-Dihydro-4,7-dimethyl-5H-2-pyrindine]	C10H13N/524-03-8	[151]
	15Hy [Pentadecane]	C15H32/629-62-9	[151]
	16Ac [Hexadecyl acetate]	C18H36O2/629-70-9	[151]
	delta1-20Ac [1-Eicosenyl acetate]	C22H42O2	[151]
	2me-1,4-hydroquinone [2-Methyl-1,4-hydroquinone]	C7H8O2/95-71-6	[146]
	isopropyl dodecanoate [Isopropyl dodecanoate]	C15H30O2/10233-13-3	[150]
	isopropyl tetradecadienoate [Isopropyl tetradecadienoate]	C17H30O2	[150]
	isopropyl stearate [lsopropyl octadecanoate]	C21H42O2/112-10-7	[150]

	isopropyl palmitate [Isopropyl hexadecanoate]	C19H38O2/142-91-6	[150]
	iridodial [2-Methyl-5-(1-oxopropan-2-yl)-cyclopentanecarbaldehyde]	C10H16O2/550-45-8	[135]
	3me-4OH [3-Methylbutan-1-ol]	C5H12O/123-51-3	[148]
	dihydronepetalactone [Hexahydro-4,7-dimethylcyclopenta[c]pyran-1(3H)-one]	C10H16O2/4581-72-0	[148]
	isodihydronepetalactone [(4.alpha,4a.alpha,7.alpha,7a.alpha)-Hexahydro-4,7-dimethylcyclopenta[c]pyranone]	C10H16O2/17672-96-7	[149]
	E8-oxo-citronellyl acetate [(E)-8-oxo-3,7-Dimethyl-6-octenyl acetate]	C12H20O3	[149]
	sec-butyl-Z3-dodecenoate [sec-Butyl-(Z)-3-dodecenoate]	C16H30O2	[147]
	2-butyl decanoate [2-Butyl decanoate]	C14H28O2/55195-24-9	[147]
	sec-butylundecanoate [sec-Butylundecanoate]	C15H30O2	[147]
	sec-propyl-Z5-dodecenoate [sec-Propyl-(Z)-5-dodecenoate]	C15H28O2	[147]
	sec-propyl-Z3-dodecenoate [sec-Propyl-(Z)-3-dodecenoate]	C15H28O2	[147]
	2-butyl dodecanoate [2-Butyl dodecanoate]	C16H32O2/6937-42-4	[147]
	sec-butyl-Z5-dodecenoate [sec-Butyl-(Z)-5-dodecenoate]	C16H30O2	[147]
	14Ald [Tetradecanal]	C14H28O/124-25-4	[146]
	delta5-14Ald [5-Tetradecenal]	C14H26O	[146]
	delta5,8-14Ald [5,8-Tetradecadienal]	C14H24O	[146]
	2-hydroxy-3me-hydroquinone [2-Hydroxy-3-methylhydroquinone]	C7H8O3	[146]
	isovaleraldehyde [3-Methylbutanal]	C5H10O/ 590-86-3	[135]
	citronellal [3,7-Dimethyl-6-octenal]	C10H18O/ 106-23-0	[135]
	2me-4Acid [2-Methylbutanoic acid]	C5H10O2/ 116-53-0	[154]
	2-hexenyl-3-me-butanoate [2-Hexenyl-3-methylbutanoate]	C11H20O2	[154]
	2-hexenyl tiglate [2-Hexenyl tiglate]	C11H18O2	[154]
	E2-6OH [(E)-2-Hexen-1-ol]	C6H12O/ 928-95-0	[154]
	isovaleric acid [3-Methylbutanoic acid]	C5H10O2/ 503-74-2	[154]

	caproic acid [Hexanoic acid]	C6H12O2/ 142-62-1	[154]
	cantharidin [2,3-Dimethyl-7-oxabicyclo[1,2,2]heptane-2,3-dicarboxylic anhydride]	C10H12O4/ 56-25-7	[180]
	R-palasonin [(R)-Palasonin]	C9H8O4	[180]
	S-palasonin [(S)-Palasonin]	C9H8O4	[180]
	8Ald [Octanal]	C8H16O/124-13-0	[154]
	2-decenyl tiglate [2-Decenyl tiglate]	C15H26O2	[154]
	delta2-8Ald [2-Octenal]	C8H14O/2363-89-5	[154]
	delta2-10Ald [2-Decenal]	C10H18O/3913-71-1	[154]
	ethyl hexadecenoate [Ethyl hexadecenoate]	C18H34O2/27710-66-3	[153]
	ethyl stearate [Ethyl octadecanoate]	C20H40O2/111-61-5	[153]
	isobutyric acid [2-Methylpropanoic acid]	C4H8O2/79-31-2	[154]
	octyl isobutyrate [Octyl-2-methylpropionate]	C12H24O2/109-15-9	[154]
	octyl 2me-butanoate [Octyl-2-methylbutanoate]	C13H26O2/29811-50-5	[154]
	8OH [Octan-1-ol]	C8H18O/111-87-5	[154]
	delta2-8Acid [2-Octenoic acid]	C8H14O2/1470-50-4	[154]
	7Ald [Heptanal]	C7H14O/111-71-7	[154]
	ethyl palmitate [Ethyl hexadecanoate]	C18H36O2/ 628-97-7	[153]
	sulcatone [6-Methyl-5-hepten-2-one]	C8H14O/110-93-0	[205]
	alpha-pinene [2,6,6-Trimethylbicyclo[3.1.1]hept-2-ene]	C10H16/ 80-56-8	[136]
	4me-6-3Kt [4-Methylhexan-3-one]	C7H14O/17042-16-9	[148]
	4me-7-3Kt [4-Methylheptan-3-one]	C8H16O/6137-11-7	[148]
	dolichodial [2-(1-Formylvinyl)-5-methylcyclopentanecarbaldehyde]	C10H14O2	[148]
	octyl isovalerate [Octyl 3-methylbutanoate]	C13H26O2/7786-58-5	[154]
	beta-caryophyllene [1R-(1R,4E,9S)-4,11,11-Trimethyl-8-methylenebicyclo[7.2.0]undec-4-ene]	C15H24/ 87-44-5	[154]

	2me2me6R8Sme-1,7-dioxaspiroundecane [(6R,8S)-2,2,8-Trimethyl-1,7-dioxaspiro[5.5]undecane]	C12H22O2	[145]
	E,E-2me8me-1,7-dioxaspiroundecane [(E,E)-2,8-Dimethyl-1,7-dioxaspiro[5.5]undecane]	C11H20O2	[145]
	7-2Kt [Heptan-2-one]	C7H14O/110-43-0	[148]
	delta1-9Hy [1-Nonene]	C9H18/124-11-8	[143]
	delta1-10Hy [1-Decene]	C10H20/872-05-9	[143]
	gamma-decalactone [5-Hexyl-dihydrofuran-2(3H)-one]	C10H18O2/706-14-9	[143]
	gamma-undecalactone [5-Heptyl-dihydrofuran-2(3H)-one]	C11H20O2/104-67-6	[143]
	pederin [Pederin]	C26H47NO8/27973-72-4	[140]
	pederone [Pederone]	C25H45NO4	[140]
	beta-necrodol [((1R,3R)-2,2,3-Trimethyl-4-methylenecyclopentyl)-methanol]	C10H18O/unvailable	[188]
	pseudopederin [Pseudopederin]	C24H43NO9/10352-73-5	[142]
	nepetalactone [Tetrahydro-4,7-dimethylcyclopenta[c]pyranone]	C10H14O2/ 490-10-8	[148]
	E2-4-oxo-6Ald [(E)-4-oxo-2-Hexenal]	C6H8O2/ 2492-43-5	[154]
	E,E-2-ethyl-7me-1,6-dioxaspirodecane [(E,E)-2-Ethyl-7-methyl-1,6-dioxaspiro[4.5]decane]	C11H20O2	[148]
	Z,E-2-ethyl-7me-1,6-dioxaspirodecane [(Z,E)-2-Ethyl-7-methyl-1,6-dioxaspiro[4.5]decane]	C11H20O2	[148]
	1me-cyclopentene [1-Methylcyclopentene]	C6H10/ 693-89-0	[148]
	2R3R-stenusine [(2R,3R)-N-Ethyl-3-(2-methylbutyl)-piperidine]	C12H25N	[376]
	2S3R-stenusine [(2S,3R)-N-Ethyl-3-(2-methylbutyl)-piperidine]	C12H25N	[376]
	2S3S-stenusine [(2S,3S)-N-Ethyl-3-(2-methylbutyl)-piperidine]	C12H25N	[376]
	2R3S-stenusine [(2R,3S)-N-Ethyl-3-(2-methylbutyl)-piperidine]	C12H25N	[376]
	norstenusine [N-Ethyl-3-isobutylpiperidine]	C11H23N	[136]
	eucalyptol [1,3,3-Trimethyl-2-oxabicyclo[2.2.2.]octane]	C10H18O/ 470-82-6	[376]
	3-hydroxy-4,5-dimethoxybenzoic acid [3-Hydroxy-4,5-dimethoxybenzoic acid]	C9H10O5/1916-08-1	[376]

TENEBRIONIDAE	toluquinone [2-Methyl-1,4-benzoquinone]	C7H6O2/553-97-9	[129]
	2-ethyl-1,4-benzoquinone [2-Ethyl-1,4-benzoquinone]	C8H8O2/ 4754-26-1	[134]
	delta1-15Hy [1-Pentadecene]	C15H30/ 13360-61-7	[129]
	2me3me-1,4-benzoquinone [2,3-Dimethyl-1,4-benzoquinone]	C8H8O2/ 526-86-3	[317]
	2-ethyl-3me-1,4-benzoquinone [2-Ethyl-3-methyl-1,4-benzoquinone]	C9H10O2	[130]
	1,4-benzoquinone [1,4-Benzoquinone]	C6H4O2/106-51-4	[125]
	delta1-11Hy [1-Undecene]	C11H22/821-95-4	[234]
	delta1-13Hy [1-Tridecene]	C13H26/ 2437-56-1	[129]
	delta1,6-15Hy [Pentadeca-1,6-diene]	C15H28/58045-15-1	[132]
	delta1,8-15Hy [Pentadeca-1,8-diene]	C15H28	[131]
	delta1,6-17Hy [Heptadeca-1,6-diene]	C17H32	[128]
	limonene [1-Methyl-4-(1-methylethenyl)-cyclohexene]	C10H16/138-86-3	[129]
	m-cresol [3-Methylphenol]	C7H8O/ 108-39-4	[126]
	3-ethylphenol [3-Ethylphenol]	C8H10O/620-17-7	[134]
	delta1-17Hy [1-Heptadecene]	C17H34/6765-39-5	[123]
	4me-6-3Kt [4-Methylhexan-3-one]	C7H14O/17042-16-9	[120]
	4me-delta1-6-3Kt [4-Methyl-1-hexen-3-one]	C7H12O	[121]
	camphene [2,2-Dimethyl-3-methylenebicyclo[2.2.1]heptane]	C10H16/79-92-5	[124]
	beta-pinene [6,6-Dimethyl-2-methylenebicyclo[3.1.1]heptane]	C10H16/127-91-3	[127]
	8-hydroxyisocoumarin [8-Hydroxyisocoumarin]	C9H6O3	[132]
	3,4-dihydro-8-hydroxyisocoumarin [3,4-Dihydro-8-hydroxyisocoumarin]	C9H8O3	[132]
	o-cresol [2-Methylphenol]	C7H8O/95-48-7	[132]
	6me-1,4-naphthoquinone [6-Methyl-1,4-naphthoquinone]	C11H8O2/605-93-6	[131]
	5me-1,4-naphthoquinone [5-Methyl-1,4-naphthoquinone]	C11H8O2	[131]

	6-ethyl-1,4-naphthoquinone [6-Ethyl-1,4-naphthoquinone]	C12H10O2	[131]
	5-ethyl-1,4-naphthoquinone [5-Ethyl-1,4-naphthoquinone]	C12H10O2	[131]
	6-propyl-1,4-naphthoquinone [6-Propyl-1,4-naphthoquinone]	C13H12O2	[129]
	6-butyl-1,4-naphthoquinone [6-Butyl-1,4-naphthoquinone]	C14H14O2	[129]
	alpha-pinene [2,6,6-Trimethylbicyclo[3.1.1]hept-2-ene]	C10H16/ 80-56-8	[234]
	heptadecadiene [Heptadecadiene]	C17H32	[234]
	me-2-hydroxy-6me-benzoate [Methyl 2-hydroxy-6-methylbenzoate]	C9H10O3/33528-09-5	[125]
	me-6-ethylsalicylate [Methyl 2-ethyl-6-hydroxybenzoate]	C10H12O3/ 55836-64-1	[125]
	propyl-1,4-benzoquinone [Propyl-1,4-benzoquinone]	C9H10O2	[129]
	delta1-19Hy [1-Nonadecene]	C19H38/18435-45-5	[129]
	E2-6Ald [(E)-2-Hexenal]	C6H10O/6728-26-3	[129]
	E2-7Ald [(E)-2-Heptenal]	C7H12O/18829-55-5	[129]
	E2-10Ald [(E)-2-Decenal]	C10H18O/3913-81-3	[129]
	9-3Kt [Nonan-3-one]	C9H18O/925-78-0	[129]
	delta1-9-3Kt [1-Nonen-3-one]	C9H16O	[129]
	6OH [Hexan-1-ol]	C6H14O/111-27-3	[129]
	2,3-me-methoxy-1,4-benzoquinone [2,3-Methylmethoxy-1,4-benzoquinone]	C8H8O3	[129]
	caprylic acid [Octanoic acid]	C8H16O2/124-07-2	[129]
	nonadecadiene [Nonadecadiene]	C19H36	[234]
	2-methoxy-3me-1,4-benzoquinone [2-Methoxy-3-methyl-1,4-benzoquinone]	C8H8O3/2207-57-0	[234]
	methyl oleate [Methyl (Z)-9-octadecenoate]	C19H36O2/112-62-9	[122]
	ethyl stearate [Ethyl octadecanoate]	C20H40O2/111-61-5	[122]
	1S-alpha-pinene [(1S)-2,6,6-Trimethylbicyclo[3.1.1]hept-2-ene]	C10H16/7785-26-4	[120]
	1R-camphene [(1R)-2,2-Dimethyl-3-methylenebicyclo[2.2.1]heptane]	C10H16/5794-03-6	[120]

	1S-beta-pinene [(1S)-6,6-Dimethyl-2-methylenebicyclo[3.1.1]heptane]	C10H16/18172-67-3	[120]
	vinyl-1,4-benzoquinone [Vinyl-1,4-benzoquinone]	C8H6O2	[120]
	alpha-terpinene [1-Isopropyl-4-methylcyclohexa-1,3-diene]	C10H16/99-86-5	[134]
TRACHYPACHIDAE	isopropyl methacrylate [Isopropyl methacrylate]	C7H12O2/4655-34-9	[116]
	ethacrylic acid [2-Methylenebutanoic acid]	C5H8O2/3586-58-1	[118]
	methacrylic acid [Methacrylic acid]	C4H6O2/79-41-4	[118]
	phenethyl methacrylate [Phenethyl methacrylate]	C12H14O2	[118]
	Z9-25Hy [(Z)-9-Pentacosene]	C25H50/69188-47-2	[118]
	6-2Kt [Hexan-2-one]	C6H12O/591-78-6	[118]
	isobutyric acid [2-Methylpropanoic acid]	C4H8O2/79-31-2	[118]
	1 - 2me-4Acid [2-Methylbutanoic acid]	C5H10O2/116-53-0	[118]
	2-phenylethanol [2-Phenylethanol]	C8H10O/60-12-8	[118]
	Z7-23Hy [(Z)-7-Tricosene]	C23H46/52078-42-9	[118]
	isovaleric acid [3-Methylbutanoic acid]	C5H10O2/ 503-74-2	[118]
	tiglic acid [(E)-2-Methyl-2-butenoic acid]	C5H8O2/ 80-59-1	[118]
	caprylic acid [Octanoic acid]	C8H16O2/124-07-2	[118]
	pelargonic acid [Nonanoic acid]	C9H18O2/112-05-0	[118]
	Z9-23Hy [(Z)-9-Tricosene]	C23H46/27519-02-4	[118]

Appendix B

Appendix 1 and 2 legends

Appendix A and B legends

Appendix A lists all chemical compounds identified in Carabidae that function as chemical defenses and repellents, including their structural formulas, CAS numbers, tribes, species, and references (https://pherobase.com/database/family/family-Carabidae.php). Appendix B provides the same information for all other beetle families (https://pherobase.com/database/order/order-Coleoptera.php). The reference list in Appendix A includes some of the works cited in the main document, the other references not cited in main document start in the appendices from number [117] to [376].

References

117. Ayer, W.A.; Bennett, M.J.; Browne, L.M.; Purdham, J.T. Defensive substances of *Coccinella transversoguttata* and *Hippodamia caseyi*, ladybugs indigenous to western Canada. *Can. J. Chem. Ecol.* **1976**, *54*, 1807–1813.

118. Attygalle, A.B.; Wu, X.; Ruzicka, J.; Rao, S.; Garcia, S.; Herath, K.; Meinwald, J.; Maddison, D.R.; Will, K.W. Defensive chemicals of two species of *Trachypachus Motschulski*. *J. Chem. Ecol.* **2004**, *30*, 577–588.

119. Attygalle, A.B.; Blankespoor, C.L.; Meinwald, J.; Eisner, T. Defensive secretion of *Tenebrio molitor* (Coleoptera: Tenebrionidae). *J. Chem. Ecol.* **1991a**, *17*, 805–809.

120. Geiselhardt, S.; Szepat, T.; Rasa, O.A.E.; Peschke, K. Defensive secretion components of the host *Parastizopus armaticeps* as kairomones for the cleptoparasite *Eremostibes opacus*. *J. Chem. Ecol.* **2006**, *32*, 767–778.

121. Howard, R.W. Chemosystematic studies of the Triboliini (Coleoptera: Tenebrionidae): phylogenetic inferences from the defensive chemicals of eight *Tribolium* spp., *Palorus ratzeburgi* (Wissmann), and *Latheticus oryzae* Waterhouse. *Ann. Entomol. Soc. Am.* **1987**, *80*, 398–405.

122. Wahrendorf, M.S.; Wink, M. Pharmacologically active natural products in the defence secretion of *Palembus ocularis* (Tenebrionidae, Coleoptera). *J. Ethnopharmacol* **2006**, *106*, 51–56.

123. Meinwald, Y.C.; Eisner, T. Defense mechanisms of arthropods. XIV. Caprylic acid: an accessory component of the secretion of *Eleodes longicollis*. *Ann. Entomol. Soc. Am.* **1964**, *57*, 513–514.

124. Roth, L.M.; Stay, B. The occurrence of para-quinones in some arthropods, with emphasis on the quinone-secreting tracheal glands of *Diploptera punctata* (Blattaria). *J. Insect Physiol.* **1958**, *1*, 305–318.

125. Gnanasunderam, C.; Young, H.; Benn, M.H. Defensive secretions of New Zealand tenebrionids - III. The identification of methyl esters of 6-methyl and 6-ethylsalicylic acid in *Chrysopeplus expolitus* (Coleoptera: Tenebrionidae). *Insect Biochem* .**1984b**, *14*, 159–161.

126. Ikan, R.; Cohen, E.; Shulov, A. Benzo- and hudroquinones in the defence secretions of *Blaps sulcata* and *Blaps wiedemanni*. *J. Insect Physiol.* **1970**, *16*, 2201–2206.

127. Holliday, A.E.; Walker, F.M.; Brodie E.D.; Formica, V.A. Differences in defensive volatiles of the forked fungus beetle, *Bolitotherus cornutus*, living on two species of fungus. *J. Chem. Ecol.* **2009**, *35*, 1302–1308.

128. Schildknecht, H.; Weiss, K.H. Mitteilung ber insektenabwehrstoffe. Ber die Tenebrioniden-chinone bei lebendem und totem untersuchungsmaterial. *Z. Naturforsch. B.* **1960b**, *15*, 757.

129. Tschinkel, W.R. A comparative study of the chemical defensive system of tenebrionid beetles: chemistry of the secretions. *J. Insect Physiol.* **1975a**, *21*, 753–783.

130. Gnanasunderam, C.; Young, H.; Hutchins, R.F.N. Defensive secretions of New Zealand tenebrionids: I. Presence of monoterpene hydrocabons in the genus *Artystona* (Coleoptera: Tenebrionidae). *J. Chem. Ecol* **1981a**, *7*, 889–894.

131. Tschinkel, W.R. 6-alkyl-1,4-naphthoquinones from the defensive secretion of the tenebrionid beetle, *Argoporis alutacea*. *J. Insect Physiol.* **1972**, *18*, 711–722.

132. Lloyd, H.A.; Evans, S.L.; Khan, A.H.; Tschinkel, W.R.; Blum, M.S. 8-hydroxyisocoumarin and 3,4-dihydro-8-hydroxyisocoumarin in the defensive secretion of the tenebrionid beetle, *Apsena pubescens*. *Insect Biochem.* **1978**, *8*, 333–336.

133. Gnanasunderam, C.; Butcher, C.F.; Hutchins, R.F.N.; Young, H. Ethyl decanoate as a major component in the defensive secretion of two New Zealand Aleocharine (Staphylinidae) beetles - *Tramiathaea cornigera* (Broun) and *Thamiaraea fuscicornis* (Broun). *J. Chem. Ecol* .**1981c,** *7*, 197–202.

134. Geiselhardt, S.; Schmitt, T.; Peschke, K. Chemical composition and pheromonal function of the defensive secretions in the subtribe Stizopina (Coleptera, Tenebrionidae, Opatrini). *Chemoecology* **2009b**, *19*,1–6.

135. Bellas, T.E.; Brown, W.V.; Moore, B.P. The alkaloid actinidine and plausible precursors in defensive secretions of rove beetles. *J. Insect Physiol.* **1974b**, 20, 277–280.

136. Lang, C.; Seifert, K.; Dettner, K. Skimming behaviour and spreading potential of Stenus species and *Dianous coerulescens* (Coleoptera: Staphylinidae). *Naturwissenschaften*. **2012**, *99*, 937–947.

137. Bitzer, C.; Brasse, G.; Dettner, K.; Schulz, S. Benzoic acid derivatives in a Hypogastrurid collembolan: temperature-dependent formation and biological significance as deterrents. *J. Chem. Ecol* .**2004**, *30*, 1591–1602.

138. Abou-Donia, S.A.; Fish, L.J.; Pattenden, G. Iridodial from the odoriferousglands of *Staphylinus olens* (Coleoptera: Staphylinidae). *Tetrahedron Lett* .**1971**, *12*, 4037–4038.

139. Fish, L.J.; Pattenden, G. Iridodial, and a new alkanone, 4-methylhexan-3-one, in the defensive secretion of the beetle, *Staphylinus olens*. *J. Insect Physiol.* **1975**, *2*, 741–744.

140. Cardani, C.; Ghiringhelli, D.; Quilico, A.; Selva, A. The structure of pederone. A novel substance from *Paederus* (Coleoptera: Staphylinidae). *Tetrahedron Lett* **1967**, *8*, 4023–4025.

141. Cardani, C.; Ghiringhelli, D.; Mondelli, R.; Pavan, M.; Quilico, A. Proprits biologiques et composition chimique delapdrine. *Ann. Soc. Entomol. Fr.* **1965b**, *1*, 813–816.

142. Cardani, C.; Ghiringhelli, D.; Yondelli, R.; Quilico, A. The structure of pederin. *Tetrahedron Lett.* **1965a**, *6*, 2537–2545.

143. Dettner, K.; Schwinger, G. Defensive secretions of three oxytelinae rove beetles (Coleoptera: Staphylinidae). *J. Chem. Ecol.* **1982**, *8*, 1411–1420.

144. Holldobler, B.; Mglich, M.; Maschwitz, U. Myrmecophilic relationship of Pella (Coleoptera: Staphylinidae) to *Lasius fuliginosus* (Hymenoptera: Formicidae). *Psyche* **1981**, *88*, 347–374.

145. Zhang, H.; Fletcher, M.T.; Dettner, K.; Francke, W.; Kitching, W. Synthesis and absolute stereochemistry of spiroacetals in rove beetles (Coleoptera: Staphylinidae). *Tetrahedron Lett.* **1999**, *40*, 7851–7854.

146. Brand, J.M.; Blum, M.S.; Fales, H.M.; Pasteels, J.M. The chemistry of the defensive secretion of the beetle, *Drusilla canaliculata*. *J. Insect Physiol.* **1973b,** *19*, 369–382.

147. Dettner, K.; Schwinger, G.; Wunderle, P. Sticky secretion from two pairs of defensive glands of rove beetle *Deleaster dichrous* (Grav.) (Coleoptera: Staphylinidae). Gland morphology, chemical constituents, defensive functions, and chemotaxonomy. *J. Chem. Ecol.* **1985a**, *11*, 859–883.

148. Huth, A.; Dettner, K. Defense chemicals from abdominal glands of 13 rove beetle species of subtribe Staphylinina (Coleoptera: Staphylinidae, Staphylininae). *J. Chem. Ecol.* **1990**, *16*, 2691–2711.

149. Jefson, M.; Meinwald, J.; Nowicki, S.; Hicks, K.; Eisner, T. Chemical defense of a rove beetle (*Creophilus maxillosus*). *J. Chem. Ecol*. **1983**, *9*, 159–180.

150. Dettner, K. Isopropylesters as wetting agents from the defensive secretion of the rove beetle *Coprophilus striatulus* F. (Coleoptera, Staphylinidae). *Insect Biochem.* **1984**, *14*, 383–390.

151. Dettner, K.Vergleichende untersuchungen zur wehrchemie und dr senmorphologie abdominaler abwehrdr sen von kurzfl glern aus dem subtribus Philonthina (Coleoptera, Staphylinidae). *Z. Naturforsch.* **1983**, *38*, 319–328.

152. Gnanasunderam, C.; Butcher, C.F.; Hutchins, R.F.N. Chemistry of the defensive secretions of some New Zealand rove beetles (Coleoptera: Staphylinidae). *Insect Biochem.* **1981b**, *11*, 411–416.

153. Steidle, J.L.M.; Dettner, K. Chemistry and morphology of the tergal gland of freeliving adult Aleocharinae (Coleoptera: Staphylinidae) and its phylogenetic significance. *Syst. Entomol.* **1993**, *18*,149–168.

154. Dettner, K.; Reissenweber, F. The defensive secretion of Omaliinae and Proteininae (Coleoptera: Staphylinidae): its chemistry, biological and taxonomic significance. *Biochem. Syst. Ecol.* **1991a**, *19*, 291–303.

155. Meinwald, J.; Roach, B.; Hicks, K.; Alsop, D.; Eisner, T. Defensive steroids from a carrion beetle (*Silpha americana*). *Experientia* **1985**, *41*, 516–519.

156. Peschke, K. Defensive and pheromonal secretion of the tergal gland of *Aleochara curtula* II. Release and inhibition of male copulatory behavior. *J. Chem. Ecol* **1983**, *9*, 13–31.

157. Peschke, K.; Metzler, M. Defensive and pheromonal secretion of the tergal gland of *Aleochara curtula.* I. The chemical composition. *J. Chem. Ecol.* **1982**, *8*, 773–783.

158. Blayte-Cerekiene, L.; Apegaite, V.; Radiute, S.; Raimondas Mozuraitis, R.; Buda, V.; Peciulyte, D. Electrophysiological and behavioural responses of *Ips typographus* (L.) to trans-4-thujanol a host tree volatile compound. *Ann. For. Sci.* **2016**, *73*, 247–256.

159. Gngora, C.E.; Tapias, J.; Jaramillo, J.; Medina, R.; Gonzalez, S.; Casanova, H.; Ortiz, A.; Benavides, P. Evaluation of terpene-volatile compounds repellent to the coffee berry borer, *Hypothenemus hampei* (Ferrari) (Coleoptera: Curculionidae). *.J Chem. Ecol.* **2020**, *46*, 881–890.

160. Pureswaran, D.S.; Borden, J.H. New repellent semiochemicals for three species of *Dendroctonus* (Coleoptera: Scolytidae). *Chemoecology* **2004a**, *14*, 67–75.

161. Yan, Z.L.; Fang, Y.L.; Sun, J.H.; Zhang, Z.N. Identification and electroantennal olfactory and behavioral tests of hindgut-produced volatiles of the red turpentine beetle, *Dendroctonus valens* LeConte (Coleoptera: Scolytidae). *Acta Entomol. Sin.* **2004**, *47*, 695–700.

162. Schrder, L.M.; Lindelw, A. Attraction of scolytids and associated beetles by different absolute amounts and proportions of a-pinene and ethanol. *J. Chem. Eco.l* **1989**, *15*, 807–817.

163. Ruther, J.; Reinecke, A.; Tolasch, T.; Hilker, M. Make love not war: a common arthropod defence compound as sex pheromone in the forest cockchafer *Melolontha hippocastani. Oecologia* **2001a**, *128*, 44–47.

164. Cortez, V.; Favila, M.E.; Verd, J.R.; and Ortiz, A.J. Behavioral and antennal electrophysiological responses of a predator ant to the pygidial gland secretions of two species of Neotropical dung roller beetles. *Chemoecology* **2012**, *22*, 29–38.

165. Aliabadi, A.; Bartelt, R.J.; Whitman, D.W. Identification of chemical defense secretion of the dung beetle *Canthon imitator*. *Int Congr Entomol* **2000**, Foz do Iguac u. *46*.

166. Hosoe, T.; Saito, K.; Ichikawa, M.; Ohba, N. Chemical defense in the firefly, *Rhagophthalmus ohbai* (Coleoptera: Rhagophthalmidae). *Appl. Entomol. Zool.* **2014**, *49*, 331–335.

167. Norton, S.A. Candy's dandy but cantharidin's quicker. *Arch. Dermatol.* **2002**, *138*,1378.

168. Eisner, T.; Smedley, S.R.; Yound, D.K.; Eisner, M.; Roach, B.; Meinwald, J. Chemical basis of courtship in a beetle (*Neopyrochroa flabellata*): cantharidin as "nuptial gift. *Proc. Natl. Acad. Sci. USA,* **1996**, *93*, 6499–6503.

169. Feng, Y.; Jianqi, M.; Zhongren, L.; Tianpeng, G. A preliminary investigation on the cantharidin resources of Shaanxi-province. *J. Northwest Sci. Tech. Univer. Agric. Forest* **1988**, *1988*–03.

170. Kurosa, K.; Watanabe, H. On the toxic substance of *Xanthochroa waterhousei*, (Coleoptera: Oedemeridae). *Jpn. J. Sanit. Zool.* **1958**, *9*, 200–201.

171. Frenzel, M.; Dettner, K. Quantification of cantharidin in canthariphilous Ceratopogonidae (Diptera), Anthomyiidae (Diptera) and cantharidin-producing Oedemeridae (Coleoptera). *J. Chem. Ecol.* **1994**, *20*, 1795–1812.

172. Holz, C.; Streil, G.; Dettner, K.; D Temeyer, J.; Boland, W. Intersexual transfer of a toxic terpenoid during copulation and its paternal allocation to developmental stages - quantification of cantharidin in cantharidin-producing oedemerids (Coleoptera, Oedemeridae) and canthariphilous pyrochroid. *Z. Naturforsch. C* **1994**, 49, 856–864.

173. Zhang, J.J.; Chen, J.J.; Li, X.X. Research progress on medicinal resources of Mylabris and close origin species. J. Chin. *Materia Medica* **2009**, *34*, 647–650.

174. Carrel, J.E.; Doom, J.P.; McCormick, J.P. Identification of cantharidin in false blister beetles (Coleoptera, Oedemeridae) from Florida. *J. Chem. Ecol.* **1986**, *12*, 741–747.

175. Dettner, K.; Schwinger, G. High 3-indoleacetic acid- and phenylacetic acid-concentrations in the pygidial glands of water beetles (Dytiscidae). *Z. Naturforsch. C* **1977b**, *32*, 453–455.

176. Dettner, K. Chemotaxonomy of water beetles based on their pygidial gland constituents. Biochem. *Syst. Ecol* **1979**, *7*, 129–140.

177. Pavan, M. Defensive secretions of Arthropoda. Final Technical Report. *Ist .Entomol. Agr. Univ. Pavia* **1968**, 1-235.

178. Mebs, D.; Pogoda, W.; Schneider, M.; Kauert, G. Cantharidin and demethylcantharidin (palasonin) content of blister beetles (Coleoptera: Meloidae) from southern Africa. *Toxicon* **2009**, *53*, 466–468.

179. Dettner, K.; Schramm, S.; Seidl, V.; Klemm, K.; Gade, G.; Fietz, O.; Boland, W. *Occurrence of terpene anhydride palasonin and palasoninimide in blister beetle Hycleus lunata* (Coleoptera: Meloidae). *Biochem. Syst Ecol* **2003**, *31*, 203–205.

180. Fietz, O.; Dettner, K.; Grls, H.; Klemm, K.; Boland, W. (R)- (+)-palasonin, a cantharidin-related plant toxin, also occurs in insect hemolymph and tissues. *J. Chem. Ecol.* **2002**, *28*,1315–1327.

181. Blodgett, S.L.; Carrel, J.E.; Higgins, R.A. Cantharidin content of blister beetles (Coleoptera: Meloidae) collected from Kansas alfalfa and implications for inducing cantharidiasis. *Environ. Entomol.* **1991**, *20*, 776–780.

182. Edwards, W.C.; Edwards, R.M.; Ogden, L.; Whaley, M. Cantharidin content of two species of Oklahoma blister beetles associated with toxicosis in horses. *Vet. Human Toxicol.* **1989**, *31*, 442–444.

183. Dixon, A.F.G.; Martin-Smith, M.; Smith, S.J. Isolation of cantharidin from *Meloe proscarabeus*. *Can. Pharm. J.* **1963**, *96*, 501–503.

184. Islami, I.; Nikbakhtzadeh, M.R. New records of canthariphily among beetles (Coleoptera) from Iran. *Turk. Entomol. Derg.* **2009**, *33*, 243–251.

185. Eisner, T.; Schroeder, F.C.; Snyder, N.; Grant, J.B.; Aneshansley, D.J.; Utterback, D.; Meinwald, J.; Eisner, M. Defensive chemistry of lycid beetles and of mimetic cerambycid beetles that feed on them. *Chemoecology* **2008**, *18*, 109–119.

186. Gonzalez, A.; Schroder, F.; Meinwald, J.; Eisner, T. N-methylquinolinium 2-carboxylate, a defensive betaine from *Photuris versicolor* fireflies. *J. Nat. Prod* **1999**, *62*, 378 –380.

187. Goetz, M.; Wiemer, D.F.; Haynes, L.R.W.; Meinwald, J.; Eisner, T. Lucibufagines. Partie I. Oxo-11-et oxo-12-bufalines, strodes d fensifs des lampyres *Photinus ignitus* et *P. marginellus* (Coleoptera: Lampyridae). *Helvet. Chim. Acta* **1979**, *62*, 1396–1400.

188. Eisner, T.; Deyrup, M.; Jacobs, R.; Meinwald, J. Necrodols: anti-insectan terpenes from defensive secretion of carrion beetle (*Necrodes surinamensis*). *J. Chem. Ecol.* **1986**, *12*, 1407–1415.

189. Dettner, K. Ecological and phylogenetic significance of defensive compounds from pygidial glands of Hydradephaga (Coleoptera). *Proc. Acad. Nat. Sci. Phil.* **1985b**, *137*, 156–171.

190. Eisner, T.; Aneshansley, D.J. Chemical defense: aquatic beetle (*Dineutus hornii*) vs. fish (*Micropterus salmoides*). *Proc. Natl. Acad. Sci. USA* **2000b**, *97*, 11313–11318.

191. Newhart, A.T.; Mumma, R.O. High-pressure liquid chromatographic techniques for the separation and quantification of norsesquiterpenes from gyrinids. *J. Chem. Ecol.* **1978**, *4*, 503–510.

192. Miller, J.R.; Hendry, L.B.; Mumma, R.O. Norsesquiterpenes as defensive toxins of whirligig beetles (Coleoptera: Gyrinidae). *J. Chem. Ecol.* **1975**, *1*, 59–82.

193. Wheeler, J.W.; Oh, S.K.; Benfield, E.F.; Neff, S.E. Cyclopentanoid norsesquiterpenes from gyrinid beetles. *J. Am. Chem. Soc.* **1972a**, *94*, 7589–7590.

194. Meinwald, J.; Opheim, K.; Eisner, T. Gyrinidal: A sesquiterpenoid aldehyde from the defensive glands of gyrinid beetles. *Proc. Natl. Acad. Sc.i USA* **1972**, *69*,1208–1210.

195. Drilling, K.; Dettner, K. First insights into the chemical defensive system of the erotylid beetle, *Tritoma bipustulata*. *Chemoecology* **2010**, *20*, 243–253.

196. Laurent, P.; Dalze, D.; Braekman, J.C.; Pasteels, J.M. Stenotarsol, a new terpenoid from *Stenotarsus subtilis* (Coleoptera: Endomychidae). *Tetrahedron Lett.* **2005a**, *46,* 931–932.

197. Dettner, K.; Beran, A. Chemical defense of the fetid smelling click beetle *Agrypnus murinus (*Coleoptera: Elateridae). *Entomol. Gen.* **2000**, *25*, 27–32.

198. Meinwald, J.; Huang, Q.; Vrkoc, J.; Herath, K.B.; Yang, Z.C.; Schroder, F.; Attygalle, A.B.; Iyengar, V.K.; Morgan, R.C.; Eisner, T. Mirasorvone: a masked 20-ketopregnane from the defensive secretion of a diving beetle (*Thermonectus marmoratus*). *Proc. Natl. Acad. Sci. USA* **1998**, *95*, 2733–2737.

199. Dettner, K. Chemical ecology and biochemistry of Dytiscidae. In Ecology, Systematics, and the Natural History of predaceous diving beetles (Coleoptera: Dytiscidae), Yee, D. (ed) *Springer, Dordrecht* **2014**, 235–306.

200. Schildknecht, H.; Holtkotte, D.; Krauss, D.; Tacheci, H. ber arthropodenabwehrstoffe, LIX. Platambin, ein wehrstoff des schwimmk fers *Platambus maculatus* (Coleoptera: Dytiscidae). *Liebig's Ann. Chem.* **1975**, 1850–1862.

201. Schildknecht, H.; Birringer, H.; Maschwitz, U. Testosteron als abwehrstoff des schlammschwimmers Ilybius. *Angew. Chem.* **1967b**, *79*, 579–580.

202. Schildknecht, H.; Tacheci, H. Colymbetin, a new defensive substance of the water beetle, *Colymbetes fuscus*, that lowers blood pressure. *J. Insect Physiol.* **1971b**, *17*, 1889–1896.

203. Schaaf, O.; Baumgarten, J.; Dettner, K. Identification and function of prothoracic exocrine gland steroids of the dytiscid beetles *Graphoderus cinereus* and *Laccophilus minutus*. *J. Chem. Ecol*. **2000a**, *26*, 2291–2305.

204. Schildknecht, H.; and Krnig, W. Protective materiaIs in the secretion from the prothoracic protective gland of a Mexican Cybister species. *Angew. Chem. Int. Ed.* **1968a**, *7*, 62–63.

205. Schildknecht, H. The defensive chemistry of land and water beetles. *Angew. Chem. Int. Ed.* **1970a**, *9*, 1–9.

206. Miller, J.R.; Mumma, R.O. Defensive agents of the American water beetles *Agabus seriatus* and *Graphoderus liberus*. *J. Insect Physiol.* **1973**, *19*, 917–925.

207. Chadha, M.S.; Joshi, N.K.; Mamdapur, V.R.; Sipahimalani, A.T. C-21 steroids in the defensive secretions of some Indian water beetles. II. *Tetrahedron* **1970**, *26*, 2061–2064.

208. Schildknecht, H.; Siewerdt, R.; Maschwitz, U. ber arthropodenabwehrstoffe, XXIII. Cybisteron, ein neues arthropoden-steroid. *Liebig's Ann. Chem.* **1967c**, *703*, 182–189.

209. Schildknecht, H.; Krnig, W. Wehrstoffe des prothorakalwehrdr sensekrets einer mexikanischen Cybister-art. *Angew. Chem.* **1968b**, *80*, 45–46.

210. Chapman, J.C.; Lockley, W.J.; Rees, H.H.; Goodwin, T.W. Stereochemistry of olefinic bond formation in defensive steroids of *Acilius sulcatus* (Dytiscidae). *Eur. J. Biochem.* **1977**, *81*, 293–298.

211. Newhart, A.T.; Mumma, R.O. Defensive secretions of three species of Acilius (Coleoptera: Dytiscidae) and their seasonal variations as determined by high-pressure liquid chromatographic. *J. Chem. Ecol.* **1979**, *5*, 643–652.

212. Jones, A.C.; Mullins, D.E.; Jones, T.H.; Salom, S.M. Characterization of physical and chemical defenses in the hemlock woolly adelgid. *J. Chem. Ecol.* **2014**, *40*, 560–568.

213. Gao, G.; Dai, L.; Gao, J.; Wang, J.; Chen, H. Volatile organic compound analysis of host and non-host poplars for *Trypophloeus klimeschi* (Coleoptera: Curculionidae: Ipinae). Russian *J. Plant Physiol.* **2018**, *65*, 916–925.

214. Ndungu, M.; Lwande, W.; Hassanali, A.; Moreka, L.; Chhabra, S.C. Cleome monophylla essential oil and its constituents as tick (*Rhipicephalus appendiculatus*) and maize weevil (*Sitophilus zeamais*) repellents. *Entomol. Exp. Appl.* **1995**, *76*, 217–222.

215. Parra, L.; Mutis, A.; Ceballos, R.; Lizama, M.; Pardo, F.; Perich, F.; Quiroz, A. *Environ. Entomol.* **2009**, *38*, 781–789.

216. King, G.A.; Meinwald, J. Review of the defensive chemistry of coccinellids. *Chem. Rev.* **1996**, *96*, 1105–1122.

217. Jones, A.C.; Mullins, D.E.; Jones, T.H.; Salom, S.M. Characterization of physical and chemical defenses in the hemlock woolly adelgid. *J. Chem. Ecol.* **2014**, *40*, 560–568.

218. Schrder, F.C.; Tolasch, T. Psylloborine A, a new dimeric alkaloid from a ladybird beetle. *Tetrahedron* **1998**, *54,* 12243-12248.

219. Tursch, B.; Dalze, D.; Braekman, J.C.; Hootele, C.; Cravador, A.; Losman D.; Karlsson R. Chemical ecology of arthropods. IX. Structure and absolute configuration of hippodamine and convergine, two novel alkaloids from the American ladybug *Hippodamia convergens* (Coleoptera - Coccinellidae). *Tetrahedron Lett.* **1974a**, *15*, 409–412.

220. Moore, B.P.; Brown, W.V.; Rothschild, M. Methylalkylpyrazines in aposematic insects, their hostplants and mimics. *Chemoecology* **1990**, *1*, 43–51.

221. Lebrun, B.; Braekman, J.C.; Dalze, D.; Kalushkov, P.; Pasteels, J.M. Hyperaspine, a new 3-oxaquinolizidine alkaloid from *Hyperaspis campestris* (Coleoptera: Coccinellidae). *Tetrahedron Lett.* **2001**, *42*, 4621–4623.

222. Eisner, T.; Ziegler, R.; McCormick, J.L.; Eisner, M.; Hoebeke, E.R.; Meinwald, J. Defensive use of an acquired substance (carminic acid) by predaceous insect larvae. *Experientia* **1994**, *50*, 610–615.

223. Alam, N.; Choi, I.S.; Song, K.S.; Hong, J.; Lee, C.O.; Jung, J.H. A new alkaloid from two coccinellid beetles *Harmonia axyridis* and *Aiolocaria hexaspilota*. *Bull. Korean Chem. Soc* **2002a**, *23*, 497–499.

224. Lebrun, B.; Braekman, J.C.; Dalze, D.; Kalushkov, P.; Pasteels, J.M. Isopsylloborine A, a new dimeric azaphenalene alkaloid from ladybird beetles (Coleoptera: Coccinellidae). *Tetrahedron Lett*. **1999**, *40*, 8115–8116.

225. Riddick, E.W.; Aldrich, J.R.; Davis, J.C. DEET repels *Harmonia axyridis* (Pallas) (Coleoptera: Coccinellidae) adults in laboratory bioassays. *J. Entomol. Sci*. **2004**, *39*, 373–386.

226. Timmermans, M.; Braekman, J.C.; Dalze, D.; Pasteels, J.M.; Merlin, J.; Declercq, J.-P. Exochomine, a dimeric ladybird alkaloid, isolated from *Exochomus quadripustulatus* (Coleoptera: Coccinellidae). *Tetrahedron Lett.* **1992b**, *33*, 1281–1284.

227. Wang, S.F.; Braekman, J.C.; Dalze, D.; Pasteels, J. Signatipennine: A new alkaloid from the New Guinean ladybird *Epilachna signatipennis* (Coccinellidae). *Bull. Soc. Chim. Belg* **1996b**, *105*, 483–487.

228. Shi, X.; Attygalle, A.B.; Xu, S.C.; Ahmad, V.U.; Meinwald, J. Synthesis and absolute configuration of 2-(12-aminotridecyl)-pyrrolidine, a defensive alkaloid from the Mexican bean beetle, *Epilachna varivestis*. *Tetrahedron* **1996**, *52*, 6859–6868.

229. Attygalle, A.B.; Xu, S.C.; McCormick, K.D.; Meinwald, J.; Blankespoor, C.L.; Eisner, T. Alkaloids of the Mexican bean beetle, *Epilachna varivestis* (Coccinellidae). *Tetrahedron* **1993c**, *49*, 9333–9342.

230. Attygalle, A.B.; McCormick, K.D.; Blankespoor, C.L.; Eisner, T.; Meinwald, J. Azamacrolides: A family of alkaloids from the pupal defensive secretion of a ladybird beetle (*Epilachna varivestis*). *Proc. Natl. Acac. Sci. USA* **1993a**, *90*, 5204–5208.

231. Radford, P.; Attygalle, A.B.; Meinwald, J.; Smedley, S.R.; Eisner, T. Pyrrolidino xazolidine alkaloids from two species of ladybird beetles. *J. Nat. Prod.* **1997**, *60*, 755 –759.

232. Brown, W.V.; Moore, B.P. Defensive alkaloids of *Cryptolaemus montrouzieri* (Coleoptera: Coccinellidae). *Aust. J. Chem.* **1982**, *35*, 1255–1261.

233. Deyrup, S.T.; Eckman, L.E.; Lucadamo, E.E.; McCarthy, P.H.; Knapp, J.C.; Smedley, S.R. Antipredator activity and endogenous biosynthesis of defensive secretion in larval and pupal *Delphastus catalinae* (Horn) (Coleoptera: Coccinellidae). *Chemoecology* **2014**, *24*, 145–157.

234. Brown, W.V.; Doyen, J.T.; Moore, B.P.; Lawrence, J.F. Chemical composition and taxonomic significance of defensive secretions of some Australian Tenebrionidae (Coleoptera). *J. Aust. Entomol. Soc.* **1992**, 31, 79–89.

235. Tursch, B.; Dalze, D.; Dupont, M.; Pasteels, J.M.; Tricot, M.C. A defense alkaloid in a carnivorous beetle. *Experientia* **1971**, *27*, 1380–1381.

236. Laurent, P.; Braekman, J.C.; Dalze, D.; Pasteels, J.M. Chilocorine D, a novel heptacyclic alkaloid from a coccinellid beetle (*Chilocorus renipustulatus*). *Tetrahedron Lett.* **2002**, *43*,7465–7467.

237. Henson, R.D.; Thompson, A.C.; Hedin, P.A.; Nichols, P.R.; Neel, W.W. Identification of precoccinellin in the ladybird beetle, *Coleomegilla maculata*. *Experientia* **1975**, *31*, 145.

238. Huang, Q.; Attygalle, A.B.; Meinwald, J.; Houck, M.A.; Eisner, T. Chilocorine C: a new "dimeric" alkaloid from a coccinellid beetle, *Chilocorus cacti*. *J. Nat. Prod.* **1998**, *61*, 598 –601.

239. Shi, X.; Attygalle, A.B.; Meinwald, J.; Houck, M.A.; Eisner, T. Spirocyclic defensive alkaloid from a coccinellid beetle. *Tetrahedron* **1995**, *51*, 8711–8718.

240. McCormick, K.D.; Attygalle, A.B.; Xu, S.C.; Svatos, A.; Meinwald, J. Chilocorine: heptacyclic alkaloid from a coccinellid beetle. *Tetrahedron* **1994**, *50*, 2365–2372.

241. Pasteels, J.M.; Deroe, B.; Tursch, B.; Braekman, J.C.; Dalze, D.; Hootele, C. Distribution et activitis des alcaloides d fensifs des Coccinellidae. *J. Insect Physiol.* **1973**, *19*, 1771–1784.

242. Braconnier, M.F.; Braekman, J.C.; Dalze, D.; Pasteels, J.M. (Z)-1,17-diaminooctadec-9-ene, a novel aliphatic diamine from Coccinellidae. *Experientia* **1985**, *41*, 519–520.

243. Lognay, G., Hemptinne, J.L.; Chan, F.Y.; Gaspar, C.H.; Marlier, M.; Braekman, J.C.; Dalze, D.; Pasteels, J.M. Adalinine, a new piperidine alkaloid from the ladybird beetles *Adalia bipunctata* and *Adalia decempunctata*. *J. Nat. Prod.* **1996**, *59*, 510–511.

244. Schultz, T.D.; Puchalski, J. Chemical defenses in the tiger beetle *Pseudoxycheila tarsalis* Bates (Carabidae: Cicindelinae). *Coleopt. Bull.* **2001**, *55*, 164–166.

245. Tursch, B.; Dalze, D.; Braekman, J.C.; Hootele, C.; Pasteels, J.M. Chemical ecology of arthropods. X. The structure of myrrhine and the biosynthesis of coccinelline. *Tetrahedron* **1975a**, *31*, 1541–1543.

246. Blum, M.S.; Jones, T.H.; House, G.J.; Tschinkel, W.R. Defensive secretions of tiger beetles: cyanogenetic basis. *Comp. Biochem. Physio.l B* **1981a**, *69*, 903–904.

247. Howard, D.F.; Phillips, D.W.; Jones, T.H.; Blum, M.S. Anthraquinones and anthrones: occurrence and defensive function in a chrysomelid beetle. *Naturwissenschaften* **1982a**, *69*, 91–92.

248. Pasteels, J.M.; Termonia, A.; Windsor, D.M.; Witte, L.; Theuring, C.; Hartmann, T. Pyrrolizidine alkaloids and pentacyclic triterpene saponins in the defensive secretions of *Platyphora* leaf beetles. *Chemoecology* **2001**, *11*, 113–120.

249. Kelley, K.C.; Schilling, A.B. Quantitative variation in chemical defense within and among subgenera of *Cicindela*. *J. Chem. Eco.l* **1998**, *24*, 451–472.

250. Pearson, D.L.; Blum, M.S.; Jones, T.H.; Fales, H.M.; Gonda, E.; White, B.R. Historical perspective and the interpretation of ecological patterns: defensive compounds of tiger beetles (Coleoptera: Cicindelidae). *Am. Naturalist* **1988**, *132*, 404–416.

251. Sears, A.L.W.; Smiley, J.T.; Hilker, M.; Muller, F.; Rank, N.E. Nesting behavior and prey use in two geographically separated populations of the specialist wasp *Symmorphus cristatus* (Vespidae: Eumeninae). *Am. Midl. Nat.* **2001**, *145*, 233–246.

252. Cceres, L.A.; Lakshminarayan, S.; Yeung, K.K.; McGarvey, B.D.; Hannoufa, A.; Sumarah, M.W.; Benitez, X.; Scott, I.M. Repellent and Attractive Effects of α-, β-, and Dihydro-β- Ionone to Generalist and Specialist Herbivores. *J. Chem. Ecol.* **2016**, *42*,107–117.

253. Sugawara, F.; Matsuda, K.; Kobayashi, A.; Yamashita, K. Defensive secretion of chrysomelid larvae *Gastrophysa atrocyanea* Motschulsky and *Phaedon brassicae* Baly. *J. Chem. Ecol.* **1979b**, *5*, 635–641.

254. Pasteels, J.M.; Rowell-Rahier, M.; Randoux, T.; Braekman, J.C.; Dal ze, D. Pyrrolizidine alkaloids of probable host-plant origin in the pronotal and elytral secretion of the leaf beetle *Oreina cacaliae*. *Entomol. Exp. Appl.* **1988**, *49*, 55–58.

255. Ehmke, A.; Rahier, M.; Pasteels, J.; Theuring, C.; Hartmann, T. Sequestration, maintenance, and tissue distribution of pyrrolizidine alkaloid N-oxides in larvae of two Oreina species. *J. Chem. Ecol-* **1999**, *25*, 2385–2395.

256. Hilker, M.; Schulz, S. Anthraquinones in different developmental stages of *Galeruca tanaceti* (Coleoptera, Chrysomelidae). *J. Chem. Ecol.* **1991**, *17*, 2323–2332.

257. Dalze, D.; Pasteels, J.M. Production of cardiac glycosides by chrysomelid beetles and larvae. *J. Chem. Ecol.* **1979**, *5*, 63–77.

258. Hammack, L.; Hibbard, B.E.; Holyoke, C.W.; Kline, M.; Leva, D.M. Behavioral response of corn rootworm adults to host plant volatiles perceived by western corn rootworm (Coleoptera: Chrysomelidae). *Environ. Entomol.* **1999**, *28*, 961–967.

259. Dobler, S.; Dalze, D.; Pasteels, J.M. Sequestration of plant compounds in a leaf beetle s defensive secretion: cardenolides in Chrysochus. *Chemoecology* **1998**, *8*, 111–118.

260. Evans, P.H.; Becerra, J.X.; Venable, D.L.; Bowers, W.S. Chemical analysis of squirt-gun defense in *Bursera* and counter defense by Chrysomelid beetles. *J. Chem. Ecol.* **2000**, *26*, 745–754.

261. Hollande, M.AC. Sur la fonction d'excretionchez les insectes salicicoles et en particulier sur l'existence des derives salicyles. *Ann. Univ. Crenoble* **1909**, *21*, 459–517.

262. Yatagai, M.; Makihara, H.; Oba, K. Volatile components of Japanese cedar cultivars as repellents related to resistance to Cryptomeria bark borer. *J. Wood. Sci* .**2002**, *48*, 51–55.

263. Laurent, P.; Braekman, J.C.; Dalze, D.; Pasteels, J.M. An ecdysteroid (22-acetyl-20-hydroxyecdysone) from the defense gland secretion of an insect: *Chrysolina carnifex* (Coleoptera: Chrysomelidae). *Chemoecology* **2003a**, *13*, 109–111.

264. Ohmura, W.; Hishiyama, S.; Nakashima, T.; Kato, A.; Makihara, H.; Ohira, T.; Irei, H. Chemical composition of the defensive secretion of the longhorned beetle, *Chloridolum loochooanum*. *J. Chem. Ecol.* **2009**, *35*, 250–255.

265. Moore, B.P.; Brown, W.V. Chemical defence in longhorn beetles of the genera *Stenocentrus* and *Syllitus* (Coleoptera: Cerambycidae). *J. Aust. Entomol. Soc.* **1971a**, *10*, 230–232.

266. Eisner, T.; Hill, D.; Goetz, M.; Jain, S.; Alsop, D.; Camazine, S.; Meinwald, J. Antifeedant action of Z-dihydromatricaria acid from soldier beetles (*Chauliognathus* spp.). *J. Chem. Ecol.* **1981**, *7*, 1149–1158.

267. Meinwald, J.; Meinwald, Y.C.; Chalmers, A.M.; Eisner, T. Dihydromatricaria acid: acetylenic acid secreted by soldier beetle. *Science* **1968b**, *160*, 890–892.

268. Schildknecht, H.; Holoubek, K.; Weis, K.H.; Kramer, H. Defensive substances of the arthropods, their isolation and identification. *Angew. Chem. In. Ed.* **1964**, *3*, 73–82.

269. Sugawara, F.; Kobayashi, A.; Yamashita, K.; Matsuda, K. Identification of octadecyl acetate and (Z)-11-eicosenyl acetate, major components of the defensive secretion of *Gastrophysa atrocyanea* Motschulsky. *Agric. Biol. Chem.* **1978**, *42*, 687–688.

270. Hori, M. Repellency of shiso oil components against the cigarette beetle, *Lasioderma serricorne* (Fabricius) (Coleoptera: Anobiidae). *Appl. Entomol. Zool.* **2004b**, *39*, 357–362.

271. Hori, M. Repellency of hinokitiol against the cigarette beetle, *Lasioderma serricorne* (Fabricius) (Coleoptera: Anobiidae). *Appl. Entomo. Zool* .**2004a**, *39*, 521–526.

272. Ojimelukwe, P.C.; Adler, C. Toxicity and repellent effects of eugenol, thymol, linalool, menthol and other pure compounds on Dinoderus bifloveatus (Coleoptera: Bostrichidae). *J. Sustain. Agric. Environ.* **2000**, *2*, 47–54.

273. Klitzke, C.F.; Trigo, J.R. New records of pyrrolizidine alkaloid-feeding insects. Hemiptera and Coleoptera on Senecio brasiliensis. *Biochem. Syst Ecol* **2000a**, *28*, 313–318.

274. Ukeh, D.A.; Umoetok, S.B.A. Repellent effects of five monoterpenoid odours against *Tribolium castaneum* (Herbst) and *Rhyzopertha dominica* (F.) in Calabar, Nigeria. *Crop. Prot.* **2011**, *30*, 1351–1355.

275. Moore, B.P.; Brown, W.V. The chemistry of the metasternal gland secretion of the common eucalypt longicorn, *Phoracantha semipuntata* (Coleoptera: Cerambycidae). *Aust. J. Chem.* **1972**, *25*, 591–598.

276. Moore, B.P.; Brown, W.V. The buprestins: bitter principles of jewel beetles (Coleoptera: Buprestidae). *J. Aust. Entomol. Soc.* **1985**, *24*, 81–85.

277. Ryczek, S.; Dettner, K.; Unverzagt, C. Synthesis of buprestins D, E, F, G and H; structural confirmation and biological testing of acyl glucoses from jewel beetles (Coleoptera: Buprestidae). *Bioorg. Med. Chem.* **2009**, *17*, 1187–1192.

278. Vidari, G.; De Bernardi, M.; Pavan, M.; Ragozzino, L. Rose oxide and iridodial from *Aromia moschata* L. (Coleoptera: Cerambycidae). *Tetrahedron Lett.* **1973**, 14, 4065–4068

279. Young, D.K. Field records and observations of insects associated with cantharidin. *Great Lakes Entomol.* **1984b**, *17*, 195–199.

280. Chandler, D.S. Use of cantharidin and meloid beetles to attract Anthicidae (Coleoptera). *Pan-Pac. Entomol.* **1976**, *52*, 179–180.

281. Kolb, A. Nahrung und Nahrungsaufnahme bei Fledermäusen. Z. *Säugetierkd* **1958**, *23*, 84–95.

282. Zimka, J. The predacity of the field frog (Rana arvalis Nilsson) and food levels in communities of soil macro-fauna of forest habitats. *Ekol. Polska* **1966**, *14*, 549–605.

283. Holliday, A. E.; Mattingly T.M.; Toro A.A.; Donald L.J.; Holliday N.J. Age- and sex-related variation in defensive secretions of adult *Chlaenius cordicollis* and evidence for their role in sexual communication. *Chemoecology* **2016**, *26*, 107–119.

284. Evans, D.L.; Schmidt, J.O. Insect defenses. Adaptative mechanisms and strategies of prey and predators. State Univ of New York Press, New York, US, **1990**; pp. 482.

285. Rork, A.M.; Renner, T. Carabidae semiochemistry: current and future directions. J. *Chem. Ecol.* **2018**, *44*, 1069–1083.

286. Lečić, S.; Ćurčić, S.; Vujisić, L.; Ćurčić, B.; Ćurčić, N.; Nikolić, Z.; Anđelković, B.; Milosavljević, S.; Tešević, V.; Makarov, S. Defensive secretions in three ground-beetle species (Insecta: Coleoptera: Carabidae). *Ann. Zool. Fennici* **2014**, *51*, 285–300.

287. Vranić, S.; Vujisić, L.; Vesović, N.; Todosijević, M.; Pavićević, M.; Radović, D.; Ćurčić S. On the diversity of semiochemicals of the pygidial gland secretions of subterranean ground beetles (Coleoptera: Carabidae). *Diversity* **2023**, *15*, 136.

288. Kanehisa, K.; Kawazu, K. Fatty acid components of the defensive substances in acid-secreting carabid beetles. *Appl. Entomol. Zool.* **1982**, *17*, 460–466.

289. Attygalle, A.B.; Wu, X.; Will, K.W. Biosynthesis of tiglic, ethacrylic, and 2-methylbutyric acids in a carabid beetle, *Pterostichus* (Hypherpes) *californicus*. *J. Chem. Ecol.* **2007**, *33*, 963–970.

290. Xu, S.; Errabeli, R.; Will, K.; Arias, E.; Attygalle, A.B. 3-Methyl-1-(methylthio)-2-butene: a component in the foul-smelling defensive secretion of two Ceroglossus species (Coleoptera: Carabidae). *Chemoecology* **2019**, *29*, 171–178.

291. Vranić, S.; Vujisić, L.; Vesović, N.; Todosijević, M.; Pantelić, D.; Pavlović, D.; Ivanović, S.; Vasovic, M.; Ćurčić, S. The morphology of the pygidial glands and the

chemical composition of their secretions of four sphodrine ground beetle species (Carabidae: Platyninae). *J. Insect Physiol.* **2024**, *158*, 104685.

292. Pearson, D.L.; Blum, M.S.; Jones, T.H.; Fales, H.M.; Gonda, E.; Witte, B.R. Historical perspective and the interpretation of ecological patterns: defensive compounds of tiger beetles (Coleoptera: Cicindelidae). *Am. Nat* .**1988**, *132*, 404–416.

293. Holliday, A.E.; Holliday, N.J.; Mattingly, T.M.; Naccarato, K.M. Defensive secretions of the carabid beetle *Chlaenius cordicollis*: chemical components and their geographic patterns of variation. *J. Chem. Ecol.* **2012**, *38*, 278–286.

294. Forsyth, D.J. The structure of the defence glands in the Dytiscidae, Noteridae, Haliplidae and Gyrinidae (Coleoptera). *Trans. Zool. Soc. London* **1968**, *120*, 159–181.

295. Vranić, S.; Ćurčić, S.; Vesović, N.; Mandić, B.; Pantelić, D.; Vasović, M.; Lazović, V.; Zhang, W.; Vujisić, L. Chemistry and morphology of the pygidial glands in four Pterostichini ground beetle taxa (Coleoptera: Carabidae: Pterostichinae). *Zoology* **2020**, *142*, 125772.

296. Vranić, S.; Vesović, N.; Vujisić, L.; Pavlović, D.; Pantelić, D.; Todosijević, M.; Ćurčić, S. Pygidial glands of three ground beetle taxa (Insecta, Coleoptera, Carabidae): a study on their morphology and chemical composition of their secretions. *Zoology* **2021**, *148*, 125948.

297. Schildknecht, H.U. Die Bombardierkäfer und ihre Explosionschemie. *Angew. Chem.* **1961**, *73*, 1–7.

298. Geiselhardt, S.F.; Peschke, K.; Nagel, P. A review of myrmecophily in ant nest beetles (Coleoptera: Carabidae: Paussinae): linking early observations with recent findings. *Naturwissenschaften* **2007**, *94*, 871–894.

299. Juliano, S.A. Habitat associations, resources, and predators of an assemblage of Brachinus (Coleoptera: Carabidae) from southeastern Arizona. *Can. J. Zoo.l* **1985**, *63*, 1683–1691.

300. Muzzi, M.; Moore, W.; Di Giulio, A. Morpho-functional analysis of the explosive defensive system of basal bombardier beetles (Carabidae: Paussinae: Metriini). *Micron* **2019**, *119*, 24–38.

301. Eisner, T.; Dean, J. Ploy and courterploy in predator-prey interactions: orb-weaving spiders versus bombardier beetles. *Proc. Nat. Acad. Sci.* **1976**, *73*(4), 1365–1367.

302. Gamberale, G.; Tullberg, B.S. Evidence for a peak-shift in predator generalization among aposematic prey. *Proc. Biol. Sci.* **1996**, *263*(1375), 1329–1334.

303. Cassola, F.; Vigna Taglianti, A. Mimicry in Cicindelini e Graphipterini africani (Coleoptera, Caraboidea). *Biogeographia* **1988**, *14*, 229–233.

304. Bonacci, T.; Zetto Brandmayr, T.; Massolo, A.; Brandmayr, P. Non aggressive behavioural interaction in larvae of the ground beetle species *Chlaenius velutinus* (Coleoptera: Carabidae). *Entomol Gen.* **2005**, 28, 213–218.

305. Greene, A. Biology of five species of Cychrini (Coleoptera: Carabidae) in the steppe region of Southeastern Washington (USA). *Melanderia* **1975**, 19, 1–43.

306. Stork, N.E. Adaptations of arboreal carabids to life in trees. *Acta Physiol. Entomol. Hung.* **1987**, 22, 273–291.

307. Speed, M.P.; Ruxton, G.D.; Mappes, J.; Sherratt, T.N. Why are defensive toxins so variable? An evolutionary perspective. *Biol. Rev.* **2012**, 87, 874–884.

308. Nenadić, M.; Soković, M.; Glamočlija, J.; Ćirić, A.; Perić-Mataruga, V.; Ilijin, L.; Tešević, V.; Vujisić, L.; Todosijević, M.; Vesović, N.; Ćurčić, S. Antimicrobial activity of the pygidial gland secretion of three ground beetle species (Insecta: Coleoptera: Carabidae). *Sci. Nat.* **2016a**, 103, 34.

309. Nenadić, M.; Soković, M.; Glamočlija, J.; Ćirić, A.; Perić-Mataruga, V.; Tešević, V.; Vujisić L.; Todosijević M.; Vesović N.; Ćurčić S. Antimicrobial activity of the pygidial gland secretion of the troglophilic ground beetle *Laemostenus* (Pristonychus) *punctatus* (Dejean, 1828) (Insecta: Coleoptera: Carabidae). *Bull. Entomol. Res*. **2016b**, *106*, 474–480.

310. Nenadić, M.; Soković, M.; Glamočlija, J.; Ćirić, A.; Vesna Perić-Mataruga, V.; Ilijin, L.; Tešević, V.; Todosijević, M.; Ljubodrag Vujisić, L.; Vesović, N.; Ćurčić, S. The pygidial gland secretion of the forest caterpillar hunter, *Calosoma* (*Calosoma*) *sycophanta*: the antimicrobial properties against human pathogens. *Appl. Microbiol. Biotechnol.* **2017b**, *101*, 977–985.

311. Armitage, M.H.; Mullisen, L. Preliminary observations of the pygidial gland of the Bombardier Beetle, *Brachinus* sp. *TJ* **2003**, *17*, 95-102.

312. Holliday, A.E.; Mattingly, T.M.; Holliday, N.J. Defensive secretions of larvae of a carabid beetle. *Physiol. Entomol*. **2015**, *40*(2), 131–137.

313. Schildknecht, H. The defensive chemistry of land and water beetles. *Angew. Chem. Int. Ed.* **1970a**, *9*, 1–9.

314. Kanehisa, K.; Murase, M. Comparative study of the pygidial defensive systems of carabid beetles. *Appl. Entomol. Zool.* **1977**, *12*, 225–235.

315. Schildknecht, H.; Maschwitz, U.; Winkler, H. ber arthropoden-abwehrstoffe, XXXII. Zur evolution der Carabiden-wehrdr sensekrete. *Naturwissenschaften* **1968d**, *55*, 112–117.

316. Scott, P.D.; Hepburn, H.R. Pygidial defensive secretions of some carabid beetles. *Insect Biochem.* **1975**, *5*, 805–811.

317. Schildknecht, H.; Holoubek, K.; Weis, K.H.; Kramer, H. Defensive substances of the arthropods, their isolation and identification. *Angew. Chem. Int. Ed.* **1964**, *3*, 73–82.

318. Eisner, T.; Jones, T.H.; Aneshansley, D.J.; Tschinkel, W.R.; Silberglied, R.E.; Meinwald, J. Chemistry of defensive secretion of bombardier beetles (Brachinini, Metriini, Ozaenini, Paussini). *J. Insect Physiol.* **1977a**, *23*, 1383–1386.

319. Pekar, S. Differential effects of formaldehyde concentration and detergent on the catching efficiency of surface-active arthropods by pitfall traps. *Pedobiologia* **2002**, *46*, 539–547.

320. Will, K.W.; Attygalle, A.B.; Herath, K. New defensive chemical data for ground beetles (Coleoptera: Carabidae): interpretations in a phylogenetic framework. *Biol. J. Linnean Soc.* **2000**, *71*, 459–481.

321. Schildknecht, H.; Holoubeck, K. Die bombardierk fer undihre explosionschemie. V. Mitteilung ber insektenabwehrstoffe. *Angew. Chem.* **1961a**, *73*,1–7

322. Schildknecht, H. Zur chemie des bombardierk fers. *Angew Chem*. **1957**, *69*, 62.

323. Lecic, S.; Curcic, S.; Vujisic, L.; Curcic, B.; Curcic, N.; Nikolic, Z.; Andelkovic, B.; Milosavljevic, S.; Teevic, V.; Makarov, S. Defensive secretions in three ground-beetle species (Insecta: Coleoptera: Carabidae). *Ann. Zool. Fennici* **2014**, *51*, 285–300.

324. McCullough, B.T. Chemical analysis of the defensive scent fluid produced by the ground beetle *Calosoma peregrinator*. *Ann. Entomol. Soc. Am.* **1969a**, *62*, 1498–1499.

325. Moore, B.P.; Wallbank, B.E. Chemical composition of the defensive secretion in carabid beetles and its importance as a taxonomic character. *Proc. R. Entomol. Soc. Lond. B* **1968b**, *37*, 62–72.

326. McCullough, B.T. Quantitative determination of salicylaldehyde in the scent fluid of *Calosoma macrum*, *C. alternans sayi*, *C. affine*, and *C. parvicollis* (Coleoptera: Carabidae). *Ann. Entomol. Soc. Am.* **1966**, *59*, 1018.

327. Casnati, G.; Pavan, M.; Ricca, A. Sulla costituzione del veleno dell'insetto *Calosoma sycophanta* L. (Coleoptera Carabidae). *Ann. Soc. Entomol. Fr.* **1965**, *1*, 705–710.

328. Vesovic, N.; Curcic, S.; Todosijevic, M.; Nenadic, M.; Zhang, W.; Vujisic, L. Pygidial gland secretions of *Carabus* Linnaeus, 1758 (Coleoptera: Carabidae): chemicals released by three species. *Chemoecology* **2020**, *30*, 59–68.

329. Adis, J.; Kramer, E. Formaldehyd-l sung attrahiert *Carabus problematicus* (Coleoptera: Carabidae). *Entomol Ger.* **1975**, *2*, 121.

330. Benn, M.H.; Lencucha, A.; Maxie, S.; Telang, S.A. The pygidial defensive secretion of *Carabus taedatus*. *J. Insect Physiol.* **1973**, *19*, 2173–2176.

331. Schildknecht, H.; Weis, K.H. Ber die chemische abwehr der aaskafer. XIV. Mitteilung ber insektenabwehrstoffe. *Z. Naturforsch.* B **1962a**, *17*, 452–455.

332. Moore, B.P. Chemical defense in carabids and its bearing on phylogeny. *Carabid Beetles* **1979a**, *2*, 193–203.

333. Xu, S.; Errabeli, R.; Will, K.; Arias, E.; Attygalle, A.B. 3-Methyl-1-(methylthio)-2-butene: a component in the foul-smelling defensive secretion of two Ceroglossus species (Coleoptera: Carabidae). *Chemoecology* **2019**, 29,171–178.

334. Eisner, T.; Hurst, J.J; and Meinwald, J. Defense mechanisms of arthropods. XI. The structure, function and phenolic secretions of the glands of a chordeumoid millipede and a carabid beetle. *Psyche* **1963b,** *70*, 94–116.

335. Eisner, T.; Deyrup, M.; Jacobs, R.; Meinwald, J. Necrodols: anti-insectan terpenes from defensive secretion of carrion beetle (*Necrodes surinamensis*). *J. Chem. Ecol.* **1986**, *12*, 1407-1415.

336. Claridge, M.F. Stridulation and defensive behaviour in the ground beetle, *Cychrus caraboides* (L.). *J. Entomol.* **1974**, *49*, 7–15.

337. Aneshansley, D.J.; Jones, T.H.; Alsop, D.; Meinwald, J.; Eisner, T. Thermal concomitants and biochemistry of the explosive discharge mechanism of some little known bombardier beetles. *Experientia* **1983**, *39*, 366–368.

338. Eisner, T.; Attygalle, A. B.; Eisner, M.; Aneshansley, D.J.; Meinwald, J. Chemical defense of a primitive Australian bombardier beetle (Carabidae): *Mystropomus regularis*. *Chemoecology* **1991b**, *2*, 29–34.

339. Balestrazzi, E.; Dazzini, M.L.V.; De Bernardi, M.; Vidari, G.; Vita-Finzi, P.; Mellerio, G. Morphological and chemical studies on the pygidial defence glands of some Carabidae (Coleoptera). *Naturwissenschaften* **1985**, *72*, 482–484.

340. Rossini, C.; Attygalle, A.B.; Gonzlez, A.; Smedley, S.R.; Eisner, M.; Meinwald, J.; Eisner, T. Defensive production of formic acid (80%) by a carabid beetle (*Galerita lecontei*). *Proc. Natl. Acad. Sci. USA*, **1997**, *94*, 6792–6797.

341. Rork, A.M.; Xu, S.; Attygalle, A.; Renner, T. Primary metabolism co-opted for defensive chemical production in the carabid beetle, *Harpalus pensylvanicus*. *J. Chem. Ecol.* **2021**, *47*, 334–349.

342. Schildknecht, H.; Weis, K.H. Die chemische natur des wehrsekretes von *Pseudophonus pubescens* und *Ps. griseus*. VIII. Mitteilung ber insektenabwehrstoffe. *Z. Naturforsch* B **1961c**, *16*, 361–363.

343. Eisner, T.; Meinwald, Y.C.; Alsop, D.W.; Carrel, J.E. Defense mechanisms of arthropods. XXI. Formic acid and n-nonyl acetate in the defensive spray of two species of Helluomorphoides. *Ann. Entomol. Soc. Am.* **1968**, *61*, 610–613.

344. Roach, B.; Dodge, K.R.; Aneshansley, D.J.; Wiemer, D.; Meinwald, J.; Eisner, T. Chemistry of defensive secretions of ozaenine and paussine bombardier beetles (Coleoptera: Carabidae). *Coleopt. Bull.* **1979**, *33*, 17–20.

345. Davidson, B.S.; Eisner, T.;Witz, B.; Meinwald, J. Defensive secretion of the carabid beetle, *Pasimachus subsulcatus*. *J. Chem. Ecol.* **1989**, *15*,1689–1697.

346. Eisner, T.; Ball, G.E.; Roach, B.; Aneschansley, D.J.; Eisner, M.; Blankespoor, C.L.; Meinwald, J. Chemical defense of an ozaenine bombardier beetle from New Guinea. *Psyche* **1989**, *96*, 153–160.

347. Wheeler, J.W.; Chung, R.H.; Oh, S.K.; Benfield, E.F.; Neff, S.E. Defensive secretions of cychrine beetle (Coleoptera: Carabidae). *Ann. Entomol. Soc. Am.* **1970**, *63*, 469–471.

348. McCullough, B.T. Analysis of the defensive scent fluid of *Pasimachus elongatus* (Coleoptera: Carabidae). *Ann. Entomol. Soc. Am.* **1972**, *65*,772.

349. McCullough, B.T. Chemical analysis of the scent fluid of *Pasimachus californicus* and *P. duplicatus* (Coleoptera: Carabidae). *Ann. Entomol. Soc. Am.* **1969c**, *62*, 1492.

350. Schildknecht, H. Koob, K. Zur explosionschemie der bombardierk fer. *Naturwissenschaften* **1969a**, *56*, 328.

351. Schildknecht, H.; Holoubeck, K. Die bombardierk fer und ihre explosionschemie. V. Mitteilung ber insektenabwehrstoffe. *Angew. Chem.* **1961a**, *73*,1–7.

352. Pavan, M. Significato chimico e biologico di alcuni veleni di insetti. *Tip. Artig. Pavia* **1958**, 1-75.

353. Kanehisa, K.; Kawazu, K. Differences in neutral components of the defensive secretion in formic-acid-secreting carabid beetles. *Appl. Entomol. Zoo.l* **1985**, *20*, 299–304.

354. Will, K.W.; Gill, A.S.; Lee, H.; Attygalle, A.B. Quantification and evidence for mechanically metered release of pygidial secretions in formic acid-producing carabid beetles. *J. Insect Sci.* **2010**, *10*, 17.

355. Attygalle, A.B.; Meinwald, J.; Liebherr, J.K.; Eisner, T. Sexual dimorphism in the defensive secretion of a carabid beetle. *Experientia* **1991d**, *47*, 296–299.

356. Casnati, G.; Pavan, M.; Ricca, A. Sulla costituzione del veleno dell'insetto *Calosoma sycophanta* L. (Coleoptera Carabidae). *Ann. Soc. Entomol. Fr.* **1965**, *1*, 705–710.

357. Moore, B.P.; Brown, W.V. Chemical composition of the defensive secretion in Dyschirius Bonelli (Coleoptera: Carabidae: Scaritinae) and its taxonomic significance. *J. Aust. Entomol. Soc.* **1979b**, *18*, 123–125.

358. Sellenschlo, U. Beifnge in borkenk fer-pheromonfallen in Norddeutschland. *Anz. Sch. Dlingskd. Pfl. Umwelt* **1986**, *59*, 152–156.

359. Moore, B.P.; Brown, W.V. Precoccinelline and related alkaloids in the Australian soldier beetle, *Chauliognathus pulchellus* (Coleoptera: Cantharidae). *Insect Biochem.* **1978**, *8*, 393–395.

360. Will, K.W.; Attygalle, A.B.; Herath, K. New defensive chemical data for ground beetles (Coleoptera: Carabidae): interpretations in a phylogenetic framework. *Biol. J. Linnean Soc.* **2000**, *71*, 459–481.

361. Brown, W.V.; Jones, A.J.; Lacey, M.J.; Moore, B.P. The chemistry of buprestins A and B. Bitter principles of jewel beetles (Coleoptera: Buprestidae). *Aust. J. Chem.* **1985**, *38*, 197–206.

362. Brown, W.V.; Lacey, M.J.; Moore, B.P. Dihydromatricariate-based triglycerides, glyceride ethers, and waxes in the Australian soldier beetle, *Chauliognathus lugubris* (Coleoptera: Cantharidae). *J. Chem. Ecol.* **1988**, *14*, 411–423.

363. Timmermans, M.; Randoux, T.; Dalze, D.; Braekman, J.C.; Pasteels, J.M.; Lesages, L. The chemical defence of Doryphorina beetles (Coleoptera: Chrysomelidae). *Biochem Syst. Ecol.* **1992a**, *20*, 343–349.

364. Vencl, F.V.; Morton, T.C. The shield defense of the sumac flea beetle, Blepharida rhois (Chrysomelidae: Alticinae). *Chemoecology* **1998**, *8*, 25–32.

365. Daloze, D.; Braekman, J.C.; Delbrassine, A.; Pasteels, J.M. Polyoxygenated steroid sophorosides from the defense glands of *Chrysolina quadrigemina*. *J. Nat. Prod* .**1991**, *54*

366. Attygalle, A.B.; Meinwald, J.; Eisner, T. Defensive secretion of a carabid beetle, *Helluomorphoides clairvillei*. *J. Chem. Ecol.* **1992**, *18*, 489–498

367. Attygalle, A.B.; Wu, X.; Maddison, D.R.; Will, K.W. Orange/lemon-scented beetles: opposite enantiomers of limonene as major constituents in the defensive secretion of related carabids. *Naturwissenschaften* **2009**, *96*, 1443–1449.

368. Eisner, T.; Swithenbank, C.; and Meinwald, J. Defense mechanisms of arthropods. VIII. Secretion of salicylaldehyde by a carabid beetle. *Ann. Entomo.l Soc. Am.* **1963c**, *56*, 37–41.

369. Lebrun, B.; Braekman, J.C.; Daloze, D.; Pasteels, J.M. 2-dehydrococcinelline, a new defensive alkaloid from the ladybird beetle *Anatis ocellata* (Coccinellidae). *J. Nat. Prod.* **1997**, *60*, 1148–1149.

370. Braekman, J.C.; Charlier, A., Daloze, D.; Heilporn, S.; Pasteels, J.; Plasman, V.; Wang, S. New piperidine alkaloids from two ladybird beetles of the genus *Calvia* (Coccinellidae). *Eur. J. Org. Chem.* **1999**, 1749–1755.

371. 372.Laurent, P.; Braekman, J.C.; Daloze, D. Enantioselective syntheses and absolute configuration of the ladybird defence alkaloids (+)-calvine and (+)-2-epicalvine. *Eur. J. Org. Chem.* **2000**, 2057–2062.

372. Shi, X.; Attygalle, A.B.; Meinwald, J. Synthesis and absolute configuration of two defensive alkaloids from the Mexican bean beetle, *Epilachna varivestis*. *Tetrahedron Lett.* **1997**, *38*, 6479–6482.

373. Dettner, K. *Defenses of Water Insects*. In Aquatic Insects, Del-Claro, K., Guillermo, R. eds. Springer, Cham. **2019**, 191–262.

374. Burger, B.V.; Petersen, W.G.B.; Tribe, G.D. Semiochemicals of the Scarabaeinae. V. Characterization of the defensive secretion of the dung beetle *Oniticellus egregius*. *Z. Naturforsch. C.* **1995b**, *50*, 681–684.

375. Meinwald, J.; Roach, B.; Eisner, T. Defensive steroids from a carrion beetle (Silpha novaboracensis). *J. Chem. Ecol.* **1987**, *13*, 35–38.

376. Schierling, A.; Seifert, K.; Sinterhauf, S.R.; Riec, J.B.; Rupprecht, J.C.; Dettner, K. The multifunctional pygidial gland secretion of the Steninae (Coleoptera: Staphylinidae): ecological significance and evolution. *Chemoecology* **2013**, *23*, 45–57.
